# Photoresponsive Organic Cages—Computationally Inspired Discovery of Azobenzene-Derived Organic Cages

**DOI:** 10.1021/jacs.4c10217

**Published:** 2024-10-22

**Authors:** Michael C. Brand, Hamish G. Trowell, James T. Pegg, Jake L. Greenfield, Magdalena Odaybat, Marc A. Little, Peter R. Haycock, Gokay Avci, Nicola Rankin, Matthew J. Fuchter, Kim E. Jelfs, Andrew I. Cooper, Rebecca L. Greenaway

**Affiliations:** † Department of Chemistry and Materials Innovation Factory, University of Liverpool, 51 Oxford Street, Liverpool L7 3NY, U.K.; ‡ Department of Chemistry, Molecular Sciences Research Hub, Imperial College London, 82 Wood Lane, London W12 0BZ, U.K.; § Universität Würzburg, Institut für Organische Chemie, Würzburg 97074, Germany; ∥ Institute of Chemical Sciences, Heriot-Watt University, Edinburgh EH14 4AS, U.K.; ⊥ Department of Chemistry, Chemistry Research Laboratory, 12 Mansfield Road, Oxford OX1 3TA, U.K.

## Abstract

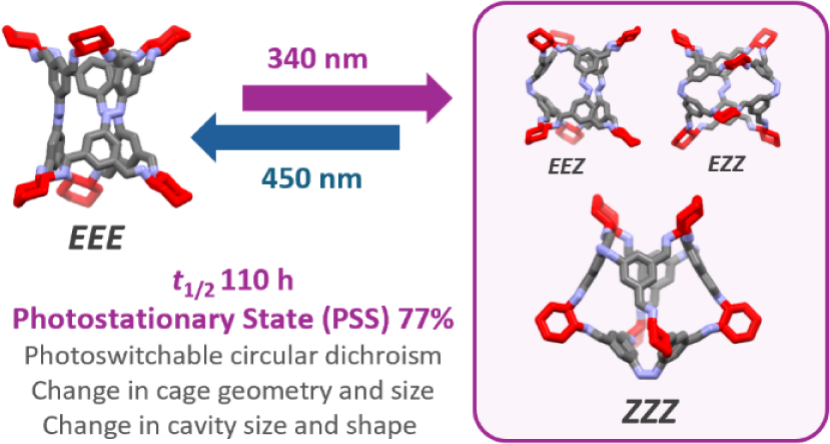

The incorporation of photoresponsive groups into porous materials is attractive as it offers potential advantages in controlling the pore size and selectivity to guest molecules. A combination of computational modeling and experiment resulted in the synthesis of two azobenzene-derived organic cages based on building blocks identified in a computational screen. Both cages incorporate three azobenzene moieties, and are therefore capable of 3-fold isomerization, using either ditopic or tetratopic aldehydes containing diazene functionality. The ditopic aldehyde forms a **Tri**^**2**^**Di**^**3**^ cage via a 6-fold imine condensation and the tritopic aldehyde forms a **Tet**^**3**^**Di**^**6**^ cage via a 12-fold imine condensation. The relative energies and corresponding intrinsic cavities of each isomeric state were computed, and the photoswitching behavior of both cages was studied by UV–Vis and ^1^H NMR spectroscopy, including a detailed kinetic analysis of the thermal isomerization for each of the *EEZ*, *EZZ* and *ZZZ* metastable isomers of the **Tet**^**3**^**Di**^**6**^ cage. Both cages underwent photoisomerization, where a photostationary state of up to 77% of the *cis*-isomer and overall thermal half-life of 110 h was identified for the **Tet**^**3**^**Di**^**6**^ species. Overall, this work demonstrates the potential of computational modeling to inform the design of photoresponsive materials and highlights the contrasting effects on the photoswitching properties of the azobenzene moieties on incorporation into the different cage species.

## 1 Introduction

Porous materials have applications in processes such as petrochemical refining^1^ and the separation of gases^2^ and solvents.^3^ In recent years, multicomponent self-assembled materials have emerged as an important class of porous materials. This includes porous coordination polymers and metal–organic frameworks (MOFs) formed through coordination chemistry, and covalent-organic frameworks (COFs) and porous organic cages (POCs) typically formed using dynamic covalent chemistries, such as imine condensations.^4^ POCs are 3-dimensional discrete organic molecules that contain a permanent internal cavity that is accessible through multiple windows.^5^ POCs can be formed into molecular solids, but lack the extended coordination or covalent bonding networks found in MOFs and COFs. However, POCs can pack together in the solid-state to form interconnected pore networks,^6^ and they have shown potential in the molecular separation of gases,^7^ hydrogen isotopes,^8^ and organic compounds.^9^ Additionally, POCs have been used as molecular sensors,^10^ and due to their discrete nature and solution processability, they have been incorporated into a new generation of porous materials known as porous liquids; that is, liquids that contain permanent intrinsic porosity.^11^

**Figure 1:**
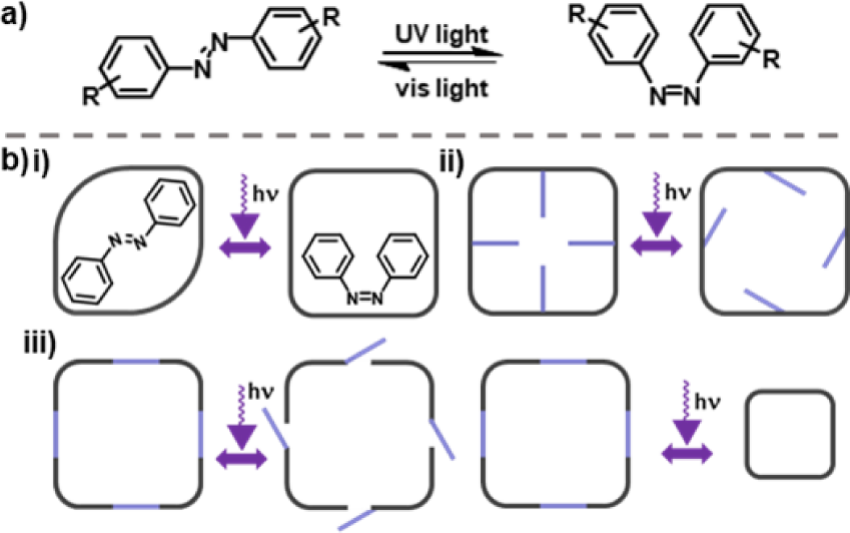
(a) Isomerization of azobenzene: the *E* → *Z* isomerization can be controlled through irradiation of the π–π* transition at a wavelength of ∼320 nm, *Z* → *E* isomerization can be controlled by irradiation of the n−π* transition at a wavelength ∼450 nm, or through heating; (b) Representation of the different methods to integrate a photoresponsive moiety (shown in light purple) into a porous material: (i) loading with a photoactive guest; (ii) decorating with photoresponsive groups; (iii) incorporation of photoresponsive groups into the scaffold itself to either introduce a gating mechanism or induce a change in size and/or shape.

The integration of stimuli-responsive moieties into porous materials such as MOFs and COFs can result in materials that are capable of a structural switch between two or more isomeric states.^12^ To induce a response, the energy input can be by photochemical irradiation,^13^ mechanical stress,^14^ or electrostatic stimulation.^15^ There are several benefits to incorporating stimuli-responsive functional groups in a porous material, including the ability to change the internal pore size allowing for on/off selectivity of gases, or to spontaneously release gas from within the porous material,^16^ or to block the pore windows enabling gas storage through a trapping technique.^17^ Several compounds undergo isomerization upon irradiation with UV–visible light (photoisomerization), including stilbenes,^18^ azobenzenes,^19^ diarylethenes,^20^ spiropyrans,^21^ and imines.^22^ Here, azobenzenes were selected for their reversible *E*–*Z* isomerization (Figure 1a) which can have a dramatic effect on the molecular geometry of the species they are incorporated into, compared to the more subtle bond rearrangements found in diarylethenes.^13b^ Azobenzenes’ resistance to photodegradation makes them suitable for multiple uptake/release photoswitching cycles in porous materials compared to stilbenes^23^ and spiropyrans.^24^ There are several methods by which these photoresponsive moieties can be integrated into a porous material (Figure 1b).^25^ The first method includes addition of a photoresponsive guest–that is, a porous material is loaded with a photoactive guest (Figure 1b(i)) and upon irradiation, a change is imposed on the host material.^26^ Another method is the incorporation of photoresponsive auxiliaries (Figure 1b(ii)). This second method typically has minimal effect in relation to a structural change, but rather, the pore can be filled, or the windows can be blocked or opened, through an *E*–*Z* isomerization.^27^ A third method of integrating a photoresponsive group is to incorporate it directly into the molecular structure itself (Figure 1b(iii)); for example, by the addition of photoresponsive linkers into a MOF.^28^

While the incorporation of photoswitchable moieties into coordination cages^29^ and macrocycles^30^ has been reported, the formation of stimuli-responsive POCs remains underexplored. Previous literature has focused on POCs formed exclusively from ditopic azobenzene precursors, linked either via imine or amine functionalities with examples demonstrating improved photostationary states upon POC formation,^31^ differential reactivity toward imine exchange reactions upon switching,^32^ and *p*-xylene separation through a crystal-to-crystal phase transition.^33^ Oshchepkov et al. studied the ability of the same amine-linked cage for anion coordination and recognition by a larger cucurbit[8]uril host.^34^ It can be envisaged that the introduction of a photoresponsive moiety into a POC could result in a dynamic reversible geometry change, leading to a controllable pore size and shape, which in turn might enable host–guest selectivity or the controlled uptake and release of different molecular guests in the cage.

Here we explore the incorporation of photoresponsive diazene precursors as one of the components of a covalent organic cage. Organic cages incorporating azobenzene functionality were first designed based on synthetically accessible precursors and investigated using computational methods, prior to investigation of synthetically viable cage structures in an experimental screen. Two azobenzene-covalent cages (ACC) were discovered; a novel **Tet**^**3**^**Di**^**6**^ tubular cage (**ACC-1**) consisting of three tetratopic (Tet^3^) aldehydes and six ditopic (Di^6^) amines, and a **Tri**^**2**^**Di**^**3**^ capsule (**ACC-2**) consisting of two tritopic (Tri^2^) amines and three ditopic (Di^3^) aldehydes, and their photoswitching behavior investigated through a series of UV–Vis and ^1^H NMR spectroscopic studies. Each cage is capable of 3-fold isomerization, using either ditopic or tetratopic aldehydes containing diazene functionality. The photoswitching behavior of the two azobenzene moieties on incorporation into the cage structures was also investigated by comparing them to single azobenzene-imine subunits. Finally, with the **Tet**^**3**^**Di**^**6**^ cage exhibiting superior photoswitching properties compared to the **Tri**^**2**^**Di**^**3**^ cage, a detailed kinetic analysis of the thermal isomerization for each of its *EEZ*, *EZZ* and *ZZZ* metastable isomers was conducted.

## 2 Results and Discussion

**Figure 2:**
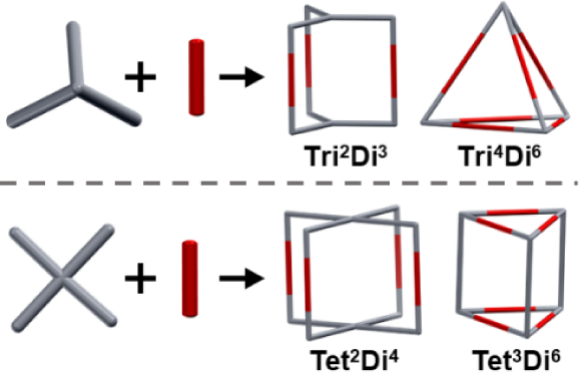
A combination of ditopic (Di), tritopic (Tri), and tetratopic (Tet) precursors and their possible assembled topological outcomes. The building block with the highest level of functionality is shown in gray in each case, the other in red.

Initially a computational screen was carried out with the aim of predicting *a priori* viable POCs that incorporate an azobenzene switch. Two azobenzene precursors were identified from the screen based on existing precursor scaffolds previously used in POC syntheses,^35^ and their synthetic viability assessed based on known routes to azobenzenes, where the azobenzene was incorporated into the scaffold rather than as a pendant group. This resulted in ditopic and tetratopic aldehydes that were assembled computationally in both their *E*- and *Z-*conformations with seven ditopic and three tritopic amines into a range of candidate organic cages. This included three cages in each of the **Tri**^**2**^**Di**^**3**^ and **Tri**^**4**^**Di**^**6**^ topologies for the ditopic aldehydes with tritopic amines, and seven cages in each of the **Tet**^**2**^**Di**^**4**^ and **Tet**^**3**^**Di**^**6**^ topologies for the tetratopic aldehydes with ditopic amines, where the superscripts signify the number of each of the precursors incorporated into the cage (Figure 2).^36^ For the tetratopic aldehyde systems, there are multiple connection possibilities, depending upon the relative arrangement of the tetratopic aldehyde. This means there are 2 positional isomers to consider for each **Tet**^**2**^**Di**^**4**^ topology and 4 for each **Tet**^**3**^**Di**^**6**^ topology. Thus, a total of 48 possible cages were modeled.

After structural model assembly using our supramolecular toolkit (*stk*) software package,^37^ the OPLS3e force field was used to geometry optimize each cage,^38^ and a basic conformer search was carried out using gas-phase molecular dynamics (MD) simulations where 50 conformations were sampled over 200 ps. During this process, the torsion angles across the diazene moiety were constrained to prevent conversion between the *E*- and *Z*-isomers. The lowest-energy candidate cages were screened for shape-persistence and symmetry using *pyWindow*, open-source code for the determination of cavity size, the number of windows and diameter of windows for porous molecules.^39^ The resulting cages were then inspected to identify promising candidates on the basis of being shape-persistent (having an internal cavity that can fit a sphere of diameter greater than 1 Å) and being largely symmetric (i.e., all window diameters within 10% of each other). As a further filter, we visually inspected the structures to prioritize candidate molecules that were not overly strained; for example, by containing out-of-plane imine bonds. Cages where both the fully *trans* (*EEE*) and fully *cis* (*ZZZ*) forms satisfied the above criteria were hypothesized to be potentially photoisomerizable, not considering at this point energy barriers to interconversion. In general, the tetratopic diazene aldehyde was found to be more likely to form a photoisomerizable cage than the ditopic diazene aldehyde. This was also promising because it suggested the potential for a larger structural photoresponse, since the **Tet**^**3**^**Di**^**6**^ topology results in larger molecular cages with more photoisomerizable linkers. Based on this computational screening, both the ditopic and tetratopic aldehyde were investigated experimentally.

A preliminary synthetic investigation was carried out into the cage formation between the tetratopic diazene aldehyde (for synthetic routes to precursors, see SI Section 2) in combination with (1*R*,2*R*)-cyclohexyldiamine (CHDA), using conditions similar to those previously reported for analogous tubular covalent cages (**TCC1–3**).^35a^ In this initial screen, both solvent and concentration were varied, using conditions previously reported in a high-throughput automated synthetic cage screen (Table S1).^35b^ Reactions were conducted in deuterated solvents to allow direct analysis prior to isolation. Species remained in solution under all conditions investigated and ^1^H NMR analysis confirmed that all reactions had gone to completion and formed a single molecular species. In each case, high-resolution mass spectrometry (HRMS) indicated clean formation of a **Tet**^**3**^**Di**^**6**^ cage species. Following this successful cage formation, the remaining 6 ditopic amines from the computational modeling were screened (Table S2), with the aim of incorporating a more flexible diamine into the cage structure, as it was thought that the highly preconfigured CHDA linker could potentially prevent the cage from having enough rotational freedom to photoisomerize. However, many of the ditopic amines screened resulted in precipitates forming over the course of the reaction, suggesting that insoluble polymer was formed–this disrupts the equilibrium of species in solution and reduces the amount of cage that can be formed in the reaction, if any cage was formed. In all of these cases, analysis by ^1^H NMR spectroscopy indicated either the reaction had not gone to completion or no soluble molecular species were present. However, HRMS indicated trace formation of a further three **Tet**^**3**^**Di**^**6**^ cages, alongside a **Tet**^**2**^**Di**^**4**^ cage that formed with 1,3-diaminopropane (Table S2).

Next, the ditopic diazene aldehyde was screened with the same selection of tritopic amines as computationally modeled (Table S3). The same conditions were used as for the previous screen with the tetratopic diazene aldehyde. In all combinations, **Tri**^**2**^**Di**^**3**^ cages were formed as indicated by HRMS. However, only the combination with tris(2-aminoethyl)amine (TREN) formed a **Tri**^**2**^**Di**^**3**^ cage in high conversion as indicated by ^1^H NMR analysis, with the other combinations containing large amounts of insoluble precipitate and residual aldehyde.

**Figure 3:**
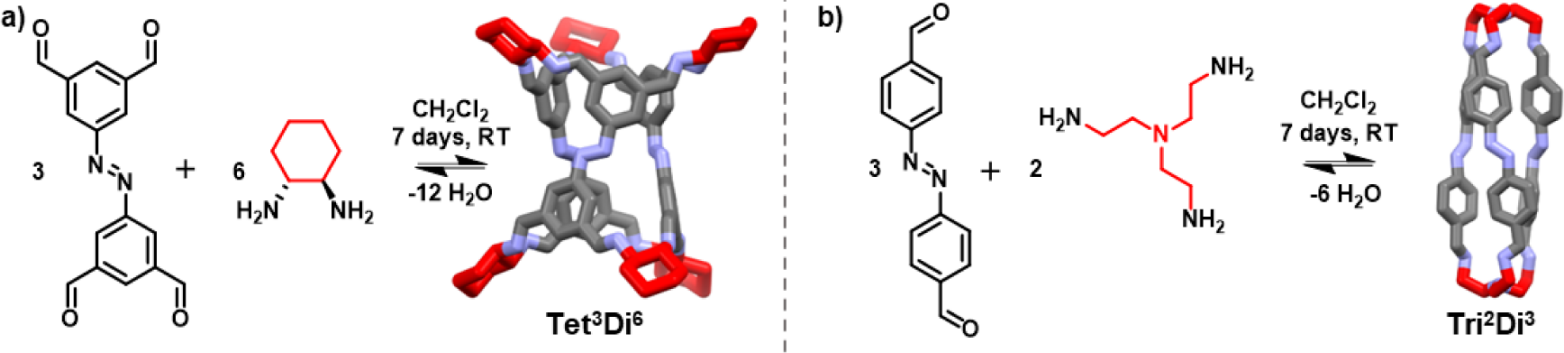
Reaction scheme for the one-pot syntheses and the corresponding crystal structures of: (a) azobenzene-covalent cage 1 (**ACC-1**) formed using 3 equiv of 5,5′-(diazene-1,2-diyl)diisophthalaldehyde and 6 equiv of CHDA in dichloromethane; (b) azobenzene-covalent cage 2 (**ACC-2**) formed using 3 equiv of 4,4′-(diazene-1,2-diyl)dibenzaldehyde and 2 equiv of TREN in dichloromethane. Hydrogens removed for clarity, aliphatic linkers shown in red, azobenzene linkers shown in gray, and nitrogens shown in blue.

Based on these synthetic screens, two azobenzene-covalent cages (**ACC**) were discovered with high conversion: **ACC-1** formed from the tetratopic aldehyde with CHDA in a **Tet**^**3**^**Di**^**6**^ topology, and **ACC-2** formed from the ditopic aldehyde with TREN as a **Tri**^**2**^**Di**^**3**^ capsule (Figure 3). Single crystal structures were grown directly from the reaction solutions by vapor diffusion with ethanol, confirming the cage topologies (SCXRD **ACC-1**Figure S2, **ACC-2**Figure S5). Porosity analysis of these crystal structures using Zeo++ and a He-sized probe (Figure S7), after removal of any solvents and disordered atoms, indicated that the internal cavity size distribution of the fully *trans* (*EEE*) isomers of **ACC-1** and **ACC-2** ranged from 2.7–4.2 Å and 1.9–2.7 Å respectively, with additional extrinsic pores of 4.22 Å also present in the former. However, isolation of the bulk material found that **ACC-1** retained no crystallinity, showing only amorphous character by PXRD (Figure S3), and **ACC-2** produced a crystalline sample that resembled the simulated powder pattern from the SCXRD data (Figure S6).

**Figure 4:**
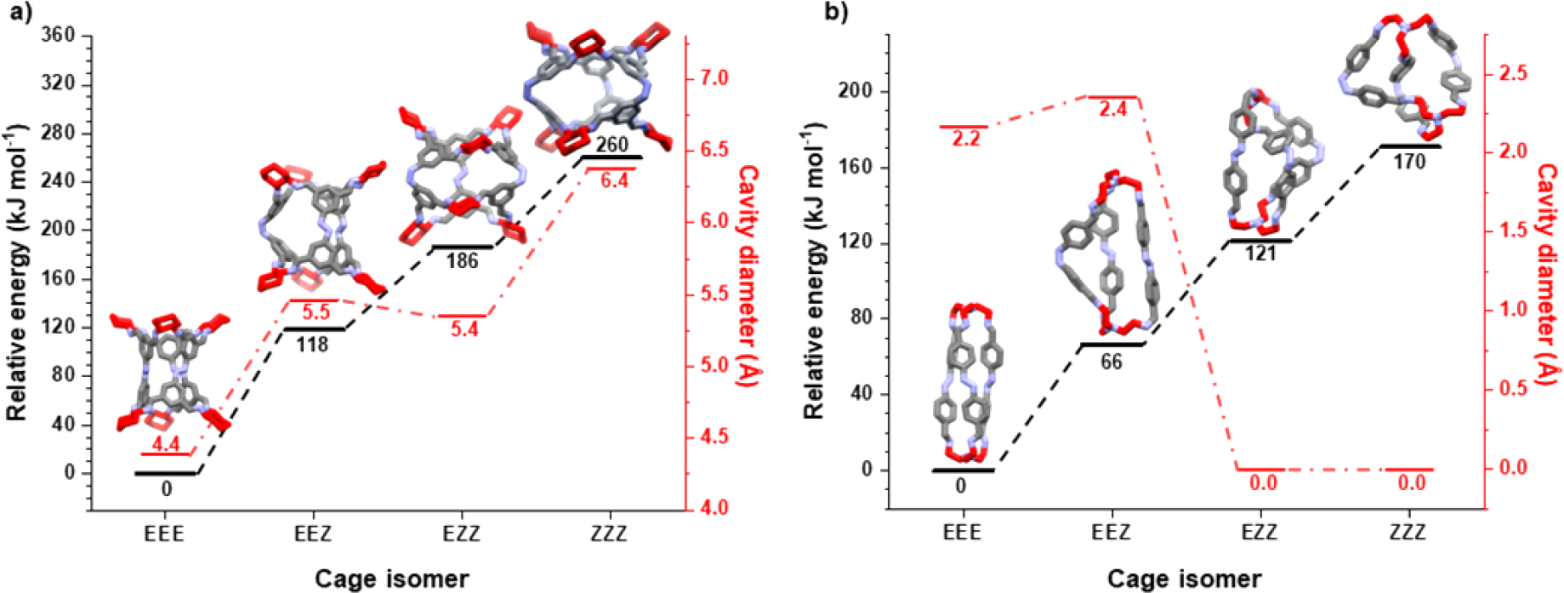
Relative energy (kJ mol^–1^) for each isomeric state, *EEE*, *EEZ*, *EZZ*, *ZZZ*, in black, and the corresponding intrinsic cavity diameters (Å) from DFT simulations at the PBE/TZVP-D3 level in red: (a) azobenzene-organic cage 1 (**ACC-1**); (b) azobenzene-organic cage 2 (**ACC-2**).

We next explored the possible range of accessible photoisomers for these two cages using computational modeling. For each cage containing three diazene moieties, four species are possible: fully *trans* (*EEE*), partially isomerized (*EEZ* and *EZZ*), and fully *cis* (*ZZZ*). Models were assembled for each of these isomers for both **ACC-1** and **ACC-2**, and longer MD conformer searches performed and the relative formation energies and internal cavity diameters compared using DFT calculations at the PBE/TZVP+D3 level (Figure 4).^40^ In both cases, the relative energy of the *ZZZ*-configuration was the highest, and the *EEE*-configuration was the lowest. The intermediate structures follow this trend, with each additional *Z*-isomer resulting in a higher relative energy. By comparison, for a triazomacrocycle reported by Heindl et al.,^30^ the relative energy of the fully *cis*-isomer was approximately 106 kJ mol^–1^ higher than the fully *trans*-isomer, and they were able to achieve 73% isomerization.

The internal cavity diameters of both cages were also calculated from these computational models. **ACC-1** has a larger internal cavity than **ACC-2** in all isomeric states. When in the fully *trans*-configuration (*EEE*), **ACC-1** has an internal spherical cavity diameter of 4.4 Å, which increases to 6.4 Å when in the *ZZZ*-configuration. The change in cavity size is not simply monotonic with isomerization, and there is a small decrease in cavity size between the *EEZ*- and *EZZ-*forms (5.5 to 5.4 Å). By contrast, there is predicted to be a complete loss of the internal cavity for **ACC-2** in its *EZZ*- and *ZZZ-*configurations, although there is an initial increase in spherical cavity diameter from *EEE* to *EEZ* of 2.2 to 2.4 Å. These computationally predicted internal cavity sizes for the fully *trans*-configuration (*EEE*) are broadly in agreement with those extracted from the SCXRDs, with the difference likely due to the SCXRDs being solvated and the computational models being in the gas phase. As well as the size, as can be seen from Figure 4, the shape of the cavity also changes considerably. In principle, both the size and shape could be exploited to allow small gas molecules to be selectively adsorbed and desorbed; unfortunately, it was found that neither of the two cages could adsorb significant quantities of N_2_ (77 K), CO_2_ or CH_4_ (273 K) as the *EEE*-isomer in the bulk amorphous phase (Figures S8, 9), which is likely due to a lack of an interconnected pore network in the amorphous state when compared to the crystal packing observed in the SCXRD for cage solvates, particularly for the **Tet**^**3**^**Di**^**6**^ species (Figure S2). In addition, the solid amorphous materials exhibited negligible solid-state photoswitching with prolonged irradiation (Figures S22, 23), or any major changes in porosity on irradiation at 365 nm of both the solid and of the cages in solution followed by removal of the solvent. However, this lack of photoresponse is not surprising, due to limited conformation freedom in the solid state inhibiting isomerization.^41^

**Figure 5:**
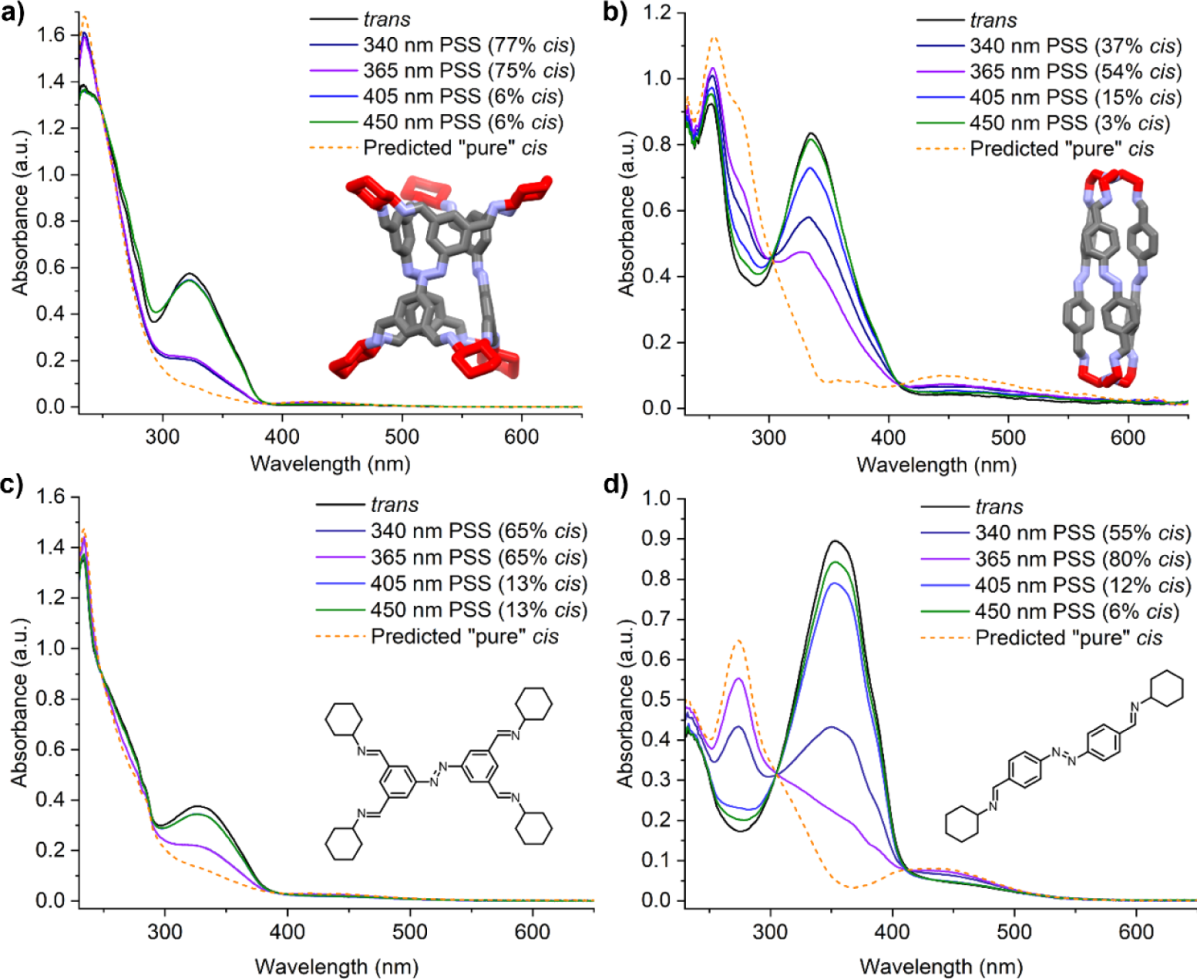
UV–Vis spectra of azobenzene-derived cages, (a) **ACC-1** and (b) **ACC-2**, measured at 25 °C in dry DCE (ca. 30 μM with respect to the azobenzene units), and single azobenzene subunits used as a comparison to their respective cages, (c) **A1** and (d) **A2**, measured at 25 °C in dry DCE (30 μM). The percentage of the cis-isomer present at the PSS of each irradiation wavelengths is shown, and the “pure” fully cis spectrum overlaid (dashed line).

With both cages producing feasibly energetically accessible^30^ calculated relative energies across the different isomer conformations, but with negligible switching observed in the solid state, the photoisomerization behavior of the cages in solution was explored using UV–Vis spectroscopy (Figure 5a,b). In each case, the cages were dissolved in dry dichloroethane (DCE) with concentrations of ca. 30 μM with respect to the azobenzene unit for both cages. Each system was irradiated with light from the initial *EEE* solution (dark state) to a *Z*-rich photostationary state (PSS). **ACC-1** was irradiated with both 340 and 365 nm light, achieving a conversion of 77% and 75% to *ZZZ*-**ACC-1** respectively. Conversion back to the *EEE*-rich solution was also successful using 405 and 450 nm light, which produced 94% of *EEE*-**ACC-1**. Irradiation of **ACC-2** with 365 nm light gave a PSS of only 54% *ZZZ*-**ACC-2**, while irradiation with 450 nm light produced a 97% *EEE*-**ACC-2** solution. The previously reported reduced variant of **ACC-2** also shows higher conversion to *ZZZ* with a reported value of 87%,^31a^ showing how the varied flexibility and electronic effects between imine and amine bonds plays an important role in the photoisomerization of cage molecules. **ACC-1** exhibited the longer thermal half-life of the two cages at 110 ± 10 h in dry DCE at 25 °C as measured by UV–Vis (Figures S10, 11), compared to 6.0 ± 0.1 h for **ACC-2** (Figures S12, 13).

The photoisomerization properties of single azobenzene subunits with imine moieties, **A1** and **A2** (Figure 5c,d), were also characterized to determine the influence of cage formation on both the PSS and thermal half-life in dry DCE (ca. 30 μM) (Figures S14–17). Interestingly, the single azobenzene unit **A1** showed lower conversion for both *E* → *Z* (65% vs 77%) and *Z* → *E* (87% vs 94%) photoswitching over **ACC-1**, while **A2** displayed a higher conversion for *E* → *Z* (80% vs 54%) and *Z* → *E* (94% vs 97%) than **ACC-2**. This suggests that there is a structural effect in the cage which influences the switching. The impact of cage formation on the thermal half-life did not affect the photoswitches similarly. At 25 °C, **A1** displayed a half-life of 220 ± 70 h—approximately double that of **ACC-1** (i.e., cage formation resulted in a less stable *Z*-isomer), while **A2** had a half-life of 4.78 ± 0.01 h, meaning that cage formation improved the overall *Z*-isomer stability. A summary of the photoswitching properties is provided in Table 1, alongside their overall enthalpy (Δ*H*^‡^) and entropy (Δ*S*^‡^) of activation obtained via Eyring plots at elevated temperatures, though caution should be applied in the physical interpretation of the transition state parameters as the *ZZZ* → *EEE* conversion is a result of multiple sequential isomerization processes.

**Table 1:** Summary of Photoswitching Properties for Cages and Azobenzene Subunits in This Study

	Tetratopic		Ditopic	
	A1	ACC-1	A2	ACC-2
Best *E*–*Z* PSS	65% *cis*	77% *cis*	80% *cis*	54% *cis*
Best *Z*–*E* PSS	13% *cis*	6% *cis*	6% *cis*	3% *cis*
*t*_1/2_ (25 °C)/h	220 ± 70	110 ± 10	4.78 ± 0.01	6.0 ± 0.1
Δ*H*^‡^/kJ mol^–1^	102 ± 7	104 ± 2	107 ± 8	83 ± 2
Δ*S*^‡^/J K^–1^ mol^–1^	-20 ± 20	-7 ± 6	30 ± 30	-51 ± 7

Due to its superior photoswitching properties, UV–Vis absorption profiles for the individual photoisomers of **ACC-1** were subsequently obtained by irradiation of a sample dissolved in dry DCE with 365 nm light and subsequent separation by HPLC (Figures S18, 19). Their individual UV–Vis spectra show an expected decrease in the π–π* absorption intensity upon isomerization to *ZZZ*-**ACC-1** (Figures S20, 21). Unexpectedly, the absorption profiles of *EEE*- and *EEZ*-**ACC-1** appear almost identical after normalization to the isosbestic point, with no apparent change in the π–π* intensity.

The impact of photoswitching on the circular dichroism of **ACC-1** was also investigated (Figures S24–26). The corresponding UV–vis absorption spectra before and after 340 nm irradiation were used to extrapolate the CD spectrum of the “pure” *cis* state, and *g*-factor of absorption (*g*_abs_) calculated accordingly. The *g*_abs_ maximum of 3.3 × 10^–3^ at 460 nm corresponds to the symmetry-forbidden n−π* band. Upon isomerization to the PSS, the dissymmetry is reduced (*g*_abs_ = 0.45 × 10^–3^), and extrapolation to the “pure” *cis* displays a sign inversion in the *g*-factor (*g*_abs_ = −1.1 × 10^–3^).

**Figure 6:**
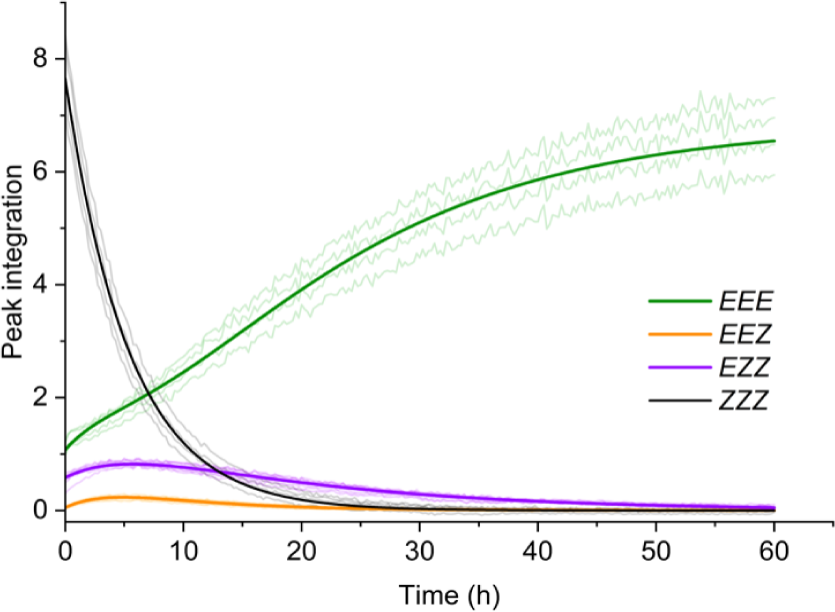
Kinetics of **ACC-1** thermal isomerization followed by ^1^H NMR spectroscopy (35 °C, dry DCE). The peak integrations (faded lines) were fit to sequential first order rates (solid lines).

Both the forward switching of **ACC-1**, and its thermal relaxation, after irradiation with 340 nm light was followed via ^1^H NMR spectroscopy in either *d*_4_-DCE at ambient temperature (Figures S27, 28) or dry DCE with solvent suppression at 35 °C (Figures 6, S29–34), respectively. The former study was used to identify each of the individual *EEE*, *EEZ*, *EZZ*, and *ZZZ* isomers of the cage. Half-lives of the intermediate photoisomers were calculated by fitting data to a stepwise series of first-order reactions, resulting in individual half-lives of: 3.74 ± 0.02 h (*ZZZ* → *EZZ*); 11.83 ± 0.08 h (*EZZ* → *EEZ*); and 3.4 ± 0.1 h (*EEZ* → *EEE*). The thermally induced isomerization of *EZZ* to *EEZ* has the longest half-life, which correlates with the comparatively smaller difference in relative energy of the photoisomers (Figure 4a). Compared to the azobenzene subunit **A1**, all photoisomers have a reduced thermal half-life due to increased cage strain energy with each additional *Z*-isomer. In addition, the individual *EEE* and *ZZZ* photoisomers of **ACC-1** were successfully resolved by diffusion NMR at 24 °C in dry DCE (Figures S35, 36; Tables S5, 6). The solvodynamic radii display a slight reduction from *EEE*-**ACC-1** (4.99 ± 0.15 Å) to *ZZZ*-**ACC-1** (4.28 ± 0.06 Å), in opposition to the increase in cavity size due to a more spherical geometry. A reduction in solvodynamic radii is also observed from *EEE*-**ACC-2** (3.44 ± 0.06 Å) to *ZZZ*-**ACC-2** (2.94 ± 0.11 Å) (Figures S37, 38; Tables S7, 8).

## 3 Conclusion

In conclusion, two photoresponsive organic cages, **ACC-1** and **ACC-2**, incorporating azobenzene functionality have been realized and their photoisomerization properties studied. Computational modeling led to the discovery of plausible photoswitchable cages using synthetically accessible precursors. By screening a range of diamines and triamines, two promising cage candidates were identified, isolated, and characterized. After successfully synthesizing these cages, a series of UV–Vis experiments were utilized to explore their photophysical properties. Both cages were found to be capable of photoisomerization, where **ACC-1** (**Tet**^**3**^**Di^6^** cage) was found to have a PSS of 77% of the *cis*-isomer with 340 nm light and had a thermal half-life of 110 ± 10 h. The second smaller cage, **ACC-2** (**Tri**^**2**^**Di**^**3**^ cage) had a PSS of 54% (365 nm) of the *cis*-isomer and a thermal half-life of 6.0 ± 0.1 h. The individual isomers of **ACC-1** were subsequently separated by HPLC, and their individual UV–Vis spectra obtained accordingly. Analysis of the photoisomers of **ACC-1** by ^1^H NMR spectroscopy enabled determination of their thermal half-lives, indicating a comparatively stable *EZZ*-isomer. The photoisomerizability of these cages was also supported by relative energy calculations, further demonstrating that computational design can be used to tackle this problem. While photoswitching was not observed in the solid-state, the realization of photoresponsive porous organic cages could lead to interesting applications in host–guest binding in solution and porous liquids.^25^

## Supporting Information Available

Computational models of azobenzene-derived organic cages (ZIP)
Experimental details and characterization data of **ACC-1** and **ACC-2**, alongside photophysical measurements (PDF)
