# Supplementary material for: Photoresponsive Organic Cages—Computationally Inspired Discovery of Azobenzene-Derived Organic Cages
Source: J Am Chem Soc. 2024 Oct 22;146(44):30332–9. doi: 10.1021/jacs.4c10217 (PMC11544616; doi:10.1021/jacs.4c10217)
Supplement: Supplementary file 2 — ja4c10217_si_002.pdf [file ja4c10217_si_002.pdf]

Supporting Information

Photoresponsive Organic Cages – Computationally Inspired Discovery of Azobenzene-Derived Organic Cages

Michael C. Brand, Hamish G. Trowell, James T. Pegg, Jake. L. Greenfield, Magdalena Odaybat, Marc A. Little, Peter R. Haycock, Gokay Avci, Nicola Rankin, Matthew J. Fuchter, Kim E. Jelfs, Andrew I. Cooper and Rebecca L. Greenaway*

Table of Contents

1. General Methods 2
2. Synthetic Procedures 8
3. Cage Screening 13
4. Photophysical Properties of Azobenzene-Derived Organic Cages by UV-Vis 25
5. Circular Dichroism of ACC-1 37
6. Photophysical Properties of Azobenzene-Derived Organic Cages by NMR 39
7. Diffusion-Ordered ^{1}H NMR Spectroscopy (DOSY) 48
8. High-Resolution Mass Spectra 52
9. ^{1}H NMR and ^{13}C NMR Spectra 53
10. References 70

1. General Methods

Materials: Chemicals were purchased from Sigma-Aldrich, Fluorochem or TCI UK and used as received. Solvents were reagent or HPLC grade purchased from Fisher Scientific or Sigma-Aldrich. All chemicals and solvents were used as received, unless otherwise specified.

Synthesis: Any reactions requiring anhydrous or inert conditions were performed in oven-dried or flame dried apparatus under an inert atmosphere of dry nitrogen, using anhydrous solvents introduced into the flask using disposable needles and syringes. All reactions were stirred magnetically using Teflon-coated stirring bars. Where heating was required, the reactions were warmed using a stirrer hotplate with heating blocks with the stated temperature being measured externally to the reaction flask with an attached probe. Removal of solvents was done using a rotary evaporator.

TLC and column chromatography: Reactions were monitored by thin layer chromatography (TLC). Spots were visualised either by an ultraviolet light, staining with potassium permanganate or staining with vanillin. Flash column chromatography was performed manually or using a Biotage Isolera with KP-Sil normal phase disposable columns.

NMR spectra: ^{1}H and ^{13}C Nuclear magnetic resonance (NMR) spectra were recorded using a Bruker Avance III 400 (400 MHz) or a Bruker Avance 500 (500 MHz) at ambient probe temperature. NMR data are presented as follows: chemical shift (expressed in ppm on a δ scale), peak multiplicity (s = singlet, d = doublet, t = triplet, q = quartet, m = multiplet), coupling constants (J / Hz) and integration. Chemical shifts are reported referenced to residual solvent where ^{1}H $CDCl_3$ (δ = 7.26 ppm), ^{13}C $CDCl_3$ (δ = 77.16 ppm), and ^{1}H $C_2D_4Cl_2$ (δ = 3.72 ppm).

For measurement of thermal isomerisation kinetics by ^{1}H NMR spectroscopy, the sample was measured at 308.15 K using a Bruker Avance III HD 500 MHz spectrometer equipped with a nitrogen-cooled Prodigy™ cryoprobe, a gradient unit providing a maximum gradient output of 53.5 G/cm and running with TopSpin 3.6.5. A series of ^{1}H experiments with 4 dummy scans, 64 scans and a delay of 1 second (total time 3 minutes 14 seconds) were collected at 15 minute intervals using a spectral width of 9014 Hz (centred on the solvent) and 32768 data points, over a total time of 60 hours. Suppression of the (non-deuterated) solvent (dry DCE) was achieved using the WATERGATE[1] w5 pulse program zggpw5 with smoothed-square shaped (SMSQ10.100) gradients of 1 ms duration and 200 µs post gradient delay. The gradient ratio for the double echo was 34% then 22%. The WATERGATE 90° pulse was 30 µs and delay for the binomial solvent suppression 111 µs (reciprocal of spectral width).

Processing was achieved with matched-filter apodisation, 256 K zero filling, and manually phase corrected in MestreNova 14.3.1. Peaks were integrated with a linear correction to the baseline.

Diffusion NMR: ^{1}H DOSY experiments were conducted at 297 K using a Bruker Avance III HD 500 MHz spectrometer equipped with a nitrogen-cooled cooled Prodigy™ cryoprobe, a gradient unit providing a maximum gradient output of 53.5 G/cm and running with TopSpin 3.6.5. Both experiments were conducted using the Bruker pulse program ledbpgp2s at a frequency of 55.013 MHz.

The spectra for **ACC-1** were collected unlocked with a spectral width of 5498.5 Hz (centred on 4.5 ppm) and 32768 data points giving an acquisition time of 2.98 seconds. A relaxation delay of 6.5 seconds was employed along with a diffusion time (Δ) of 20 ms and a longitudinal eddy current delay (LED) of 5 ms. Bipolar gradient pulses (δ/2) of 2.4 ms and homospoil gradient pulses of 0.6 ms were used. The gradient strengths of the two homospoil pulses were -17.13% and -13.17%. 16 experiments of 80 transients were collected with bipolar gradient strength, initially at 2% (1st experiment), linearly increased to 95% (16th experiment). All gradient pulses were smoothed-square shaped (SMSQ10.100) and after each application a recovery delay of 200 µs used.

The spectra for **ACC-2** were collected locked to the residual solvent peak with a spectral width of 6009.62 Hz (centred on 5 ppm) and 32768 data points giving an acquisition time of 2.73 seconds. A relaxation delay of 8.3 seconds was employed along with a diffusion time (Δ) of 20 ms and a longitudinal eddy current delay (LED) of 5 ms. Bipolar gradient pulses (δ/2) of 1.4 ms and homospoil gradient pulses of 0.6 ms were used. The gradient strengths of the two homospoil pulses were -17.13% and -13.17%. 16 experiments of 32 transients were collected with bipolar gradient strength, initially at 2% (1st experiment), linearly increased to 95% (16th experiment). All gradient pulses were smoothed-square shaped (SMSQ10.100) and after each application a recovery delay of 200 µs used.

The data for both experiments was processed using 32768 data points in the direct dimension applying an exponential function with a line broadening of 1 Hz and 64 data points in the indirect dimension. Further processing was achieved using the Bruker Dynamics Center software (version 2.8.b.4)—error estimation by Monte Carlo simulation was applied with a confidence level of 95%.

Solvodynamic radii were calculated using a variation of the Stokes-Einstein equation:

$$R_S = \frac{k_B T}{6\pi\eta D}$$

Where: R_S is the solvodynamic radius (m)

k_B is the Boltzmann constant (1.38×10^{-23} J K^{-1})

T is the temperature (K)

η is the sample viscosity (which was measured as below)

D is the diffusion coefficient ($m^2\ s^{-1}$)

Viscosity measurements: Viscosity measurements were conducted on a calibrated RheoSense microVISC viscometer (0.01–100 cP) with a minimum of three repeats.

HRMS: Electrospray ionization mass spectrometry (ES-MS) was carried out using an Agilent Technologies 6530B accurate-mass QTOF Dual ESI mass spectrometer (MeOH + 0.1% formic acid, capillary voltage 4000 V, fragmentor 225 V) in positive-ion detection mode for cage samples. For all other compounds the departmental analytical services were used and were run using an Agilent Technologies QTOF 7200 or Agilent Technologies QTOF 6540.

HPLC: High-performance liquid chromatography (HPLC) was performed using an Agilent 1260 Infinity II Hybrid SFC/UHPLC with an Agilent Zorbax 5µm SB-C8 4.6 × 5.0 mm reversed-phase HPLC column. Separation was achieved using 65–95% MeCN/H_2O (5 mM ammonium formate) over a 4 minute gradient with a 1.2 $mL\,min^{-1}$ flow rate. **ACC-1** was dissolved in dry DCE, irradiated with 365 nm light for 60 minutes in an HPLC vial, then separated by HPLC. Smoothing of the resulting UV-Vis spectra was applied using a Savitzky–Golay filter with a window size of 20 points and a polynomial order of 5 in OriginPro 2024.

Melting points: Obtained using Stuart SMP10 digital melting point apparatus and are reported uncorrected.

IR spectra: Infra-red (IR) spectra were recorded on a Bruker Alpha Platinum-ATR with measurements for oils and solids as neat samples.

Single crystal X-ray Diffraction: ACC-1 was crystallised from CH_2Cl_2 and EtOH solvent mixture at room temperature. **ACC-2** was crystallised from CH_2Cl_2 and MeOH solvent mixture at room temperature. Single crystal X-ray data sets were measured using a Rigaku MicroMax-007 HF rotating anode diffractometer (Mo-Kα radiation, λ = 0.71073 Å, Kappa 4-circle goniometer, Rigaku Saturn724+ detector). The solvated single crystals, isolated from the crystallization solvent, were immersed in a protective oil, mounted on a MiTeGen loop, and flash-cooled under a dry nitrogen gas flow. Single crystal X-ray diffraction frames were processed in CrysAlisPro. Structures were solved with SHELXT[2] and refined by full-matrix least-squares on $|F|^2$ by SHELXL,[2] interfaced through the programme OLEX2.[3] All non-H-atoms were refined anisotropically, and H-atoms were fixed in geometrically estimated positions and refined using the riding model. For full refinement details, see **Supplementary**

Table S4. Single crystal X-ray diffraction data for **ACC-1** was refined with a 1 Å resolution, and due to the disorder and limited quality of the X-ray data, it was not possible to locate the solvent molecules in large voids in the crystal structure. Therefore, a solvent mask generated using the SQUEEZE routine in Platon[4,5] was applied during the final refinement cycles **ACC-1**; see the supporting CIF file for complete details. Single crystal X-ray diffraction data for **ACC-2** was refined with a 0.95 Å resolution; see the supporting CIF file for complete details.

PXRD: Powder X-ray diffraction data were collected in transmission mode on samples held on a metal 96-shallow well plate on a Panalytical X'Pert PRO MPD equipped with a high-throughput screening (HTS) XYZ stage, Xray focusing mirror and PIXcel detector, using Ni-filtered Cu Kα radiation. Data were measured over the range of 4–50° in ~0.013° steps over 20 minutes.

Gas Sorption Analysis: Isotherms were collected using a Micromeritics 3Flex volumetric adsorption analyser fitted with a temperature-controlled cryostat unit. Samples were degassed at 90 °C for 24 h under dynamic vacuum (10^{-5} bar) prior to analysis. Samples were then allowed to equilibrate to the analysis temperatures (N_2 at 77 K, CO_2 at 273 K, and CH_4 at 273 K) before analysis was initiated. Upon completion of the analysis, samples were degassed once more and reweighed.

UV-Vis: UV-visible absorption spectra were measured on a Shimadzu UV-2550 UV-vis spectrometer and an Agilent Cary 60 UV-vis spectrophotometer equipped with a Peltier temperature controller.

Photoswitching: Samples were irradiated in solution using a custom-built irradiation setup provided by Sahlmann Photochemical Solutions. Irradiation of 340 nm light was achieved using Seoul CUD4AF1B LEDs (3 × 50 mW), 365 nm light using Nichia NCSU276A LEDs (3 × 800 mW), 405 nm light using Nichia NCSU119C LEDs (3 × 770 mW) and 450 nm using Nichia NCSC219B-V1 LEDs (3 × 900 mW). Samples were irradiated until no further changes in the UV-vis absorption spectra were observed, indicating that a photostationary state was achieved.

UV-Vis absorption spectra of the pure *trans* (dark) state and of samples after irradiation were used to calculate the PSS via the Fischer method.[6] This method assumes that the ratio of isomerization quantum yields between *E* and *Z* isomers ($\Phi_{E\to Z}/\Phi_{Z\to E}$) does not differ at the different irradiation wavelengths—an assumption that has been verified experimentally for aurone and indigoid photoswitches,[7–9] and is used extensively for PSS determination of photoswitches (including azobenzenes).[10–12] Predicted "pure" cis spectra were determined by linear extrapolation based on knowledge of the *trans* spectrum, PSS spectrum, and the ratio of *E*/*Z* isomers at the PSS:

$$Predicted\ pure\ cis = trans\ spectrum + \frac{PSS\ spectrum - trans\ spectrum}{PSS\ ratio}$$

Thermal half-life measurements were measured using UV-vis spectroscopy (Agilent Cary 60 equipped with a Peltier temperature controller). Samples were equilibrated at temperature for 10 minutes, with stirring, and then irradiated with light to achieve the PSS. Time course measurements were then performed recording spectra at set intervals.

Circular dichroism: Circular dichroism (CD) and corresponding UV-Vis absorption measurements of **ACC-1** were conducted using an Applied Photophysics Chirascan V100 spectrophotometer in a quartz cuvette (10 mm × 10 mm).

The PSS from the corresponding UV-Vis absorption spectra was calculated by the Fischer method. The predicted "pure" *cis* ellipticity was calculated by linear extrapolation from knowledge of the *trans* spectrum, PSS spectrum and PSS ratio in a manner analogous to extrapolation of the UV-Vis absorbance spectrum.

The dissymmetry factor (g_{abs}) was calculated from the CD spectra: $g_{abs} = (Elipticity\ [m°])/(Absorbance\ [a.u.] \times 32980)$.

Computational methodology: Initial structures were built using *stk*, the supramolecular toolkit,[13] python software that can automate the assembly and conformer searching for organic cages. For a rapid computational initial screen, starting from the initial structures from *stk*, a molecular dynamics simulation was performed in Macromodel with OPLS3e[14] at 750 K for 200 ps with a timestep of 1 fs with structures sampled every 4 ps and each of these structures was geometry optimised. The relevant torsion of the azo-group was held fixed during the MD simulation to ensure they maintained either the *cis* or *trans* conformation, as needed. Only the lowest energy conformation was used for further structural analysis. For more detailed analysis of the experimentally realised cages, longer MD simulations were carried out for 10 ns, sampled every 1 ps, with each sampled conformer undergoing geometry optimisation to find the lowest energy conformation. To then get more accurate relative energies, density functional theory (DFT) calculations were carried out in CP2K[15] with the PBE functional, [16] TZVP basis set[17] and a Grimme D3 dispersion correction[18] and a plane-wave cutoff of 350 Ry. Overall, this approach has been used for several previous combined experimental and computational studies that have successfully discovered new porous organic imine cages.[19,20] The pore volume and diameter of each cage are calculated with our Python software, *pyWindow*.[21] This analysis assumes a spherical probe and a single pore, whose position is optimized to maximize the sphere fit in the pore.

Structural properties of the experimental cages, pore size diameter, the largest cavity diameter, pore limiting diameter and pore size derivative distributions were calculated using the Zeo++ software.[22]

He-sized probe was used to calculate the porosity. Pore channels were visualised with Mercury software.[23] Any solvents and disordered atoms have been cleaned before Zeo++ calculations.

2. Synthetic Procedures

5-Nitroisophthalaldehyde (1)

Isophthalaldehyde (4.0 g, 30 mmol, 1.0 eq.) was dissolved in H_2SO_4 (98%, 13 mL) and cooled to 0 °C in

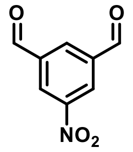

an ice bath. A mixture containing H_2SO_4 (98%, 4 mL) and HNO_3 (65%, 8 mL) was added dropwise over the course of 2 hours. The resulting mixture was then heated to 50 °C for 1 hour. After cooling to room temperature, the mixture was then poured directly onto ice, then the precipitate was filtered and washed with water (300 mL). The resulting solid was left to dry under a continuous air flow to afford the desired product **1** as a colourless solid (4.6 g, 26 mmol, 87%) and was used without further purification.

^{1}H NMR (400 MHz, $CDCl_3$) δ 10.21 (s, 2H), 8.96 (d, *J* = 1.4 Hz, 2H), 8.72 (t, *J* = 1.4 Hz, 1H); **^{13}C NMR** (101 MHz, $CDCl_3$) δ 188.65, 149.63, 138.41, 134.71, 128.70; Data in accordance with literature values.[24]

2,2'-(5-Nitro-1,3-phenylene)bis(1,3-dioxolane) (2)

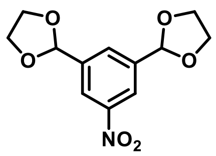

5-Nitroisophthalaldehyde **1** (2.5 g, 14 mmol, 1.0 eq.), ethylene glycol (2.06 mL, 37 mmol, 2.6 eq.) and *p*-toluenesulfonic acid (0.4 g, 2 mmol, 0.14 eq.) were dissolved in toluene (50 mL). The reaction mixture was heated to reflux for 16 hours, fitted with a pre-filled Dean-Stark apparatus. After cooling to room temperature, the toluene was removed *in vacuo.* Water was added to the solid mixture, and the solid collected by filtration under vacuum and washed with additional water (200 mL). The resulting solid was left to dry under continuous air flow to afford the desired product **2** as a colourless solid (3.3 g, 12.9 mmol, 92%) and was used without further purification.

MP 57 - 60 °C; **IR** (ν_{max}/cm^{-1}) 2954, 2892, 1532, 1465, 1344, 1164, 1083, 967, 881, 725, 706, 655; **^{1}H NMR** (400 MHz, $CDCl_3$) δ 8.34 (d, *J* = 1.5 Hz, 2H), 7.91 (t, *J* = 1.4 Hz, 1H), 5.90 (s, 2H), 4.15 – 4.05 (m, 8H); **^{13}C NMR** (101 MHz, $CDCl_3$) δ 148.49, 140.85, 130.84, 122.29, 102.24, 65.63; **HRMS** (CI) calc. for $C_{12}H_{13}NO_6$ $[M+H]^+$ 268.0816, found 268.0820.

(*E*)-1,2-Bis(3,5-di(1,3-dioxolan-2-yl)phenyl)diazene (3)

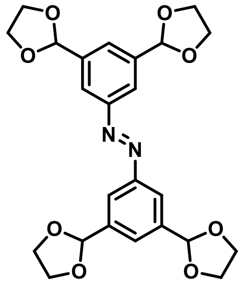

2,2'-(5-Nitro-1,3-phenylene)bis(1,3-dioxolane) **2** (0.99 g, 5.8 mmol, 1.0 eq.), sodium hydroxide (0.9 g, 23.2 mmol, 4.0 eq.), and zinc dust (0.7 g, 11.6 mmol, 2.0 eq.) was dissolved in distilled ethanol (25 mL) and water (12.5 mL). The reaction mixture was heated to 100 °C for 20 hours. The reaction mixture was diluted with water and neutralized with 1 M HCl. The crude mixture was filtered and washed with water. The filtrate was separated with chloroform (3x100 mL), and the organic layers combined. The combined organic layers were dried (Na_2SO_4) and concentrated *in vacuo*. The crude product was purified by column chromatography (gradient petroleum ether/EtOAc 0-50%). This afforded the desired product **3** as an orange/brown solid (0.57 g, 1.2 mmol, 41%).

MP 155 - 157 °C; **IR** (ν_{max}/cm^{-1}) 2886, 1369, 1165, 1007, 939, 882, 701; **^{1}H NMR** (400 MHz, $CDCl_3$) δ 8.06 (d, *J* = 1.6 Hz, 4H), 7.73 (t, *J* = 1.5 Hz, 2H), 5.95 (s, 4H), 4.19 – 4.13 (m, 8H), 4.10 – 4.06 (m, 8H); **^{13}C NMR** (101 MHz, $CDCl_3$) δ 152.77, 139.88, 127.25, 121.88, 103.22, 65.51; **HRMS** (ES+) calc. for $C_{24}H_{26}N_2O_8$ $[M+H]^+$ 471.1762, found 471.1760.

(*E*)-5,5'-(Diazene-1,2-diyl)diisophthalaldehyde (4)

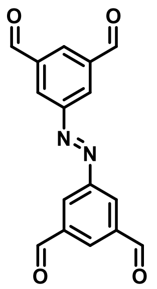

(*E*)-1,2-Bis(3,5-di(1,3-dioxolan-2-yl)phenyl)diazene **3** (0.4 g, 1 mmol, 1.0 eq.) and *p*-toluenesulfonic acid (0.02 g, 0.1 mmol, 0.1 eq.) were dissolved in a 3:1 mix of acetone/water (20 mL). The reaction mixture was heated to 60 °C for 18 hours. After cooling to room temperature, the acetone was removed *in vacuo.* The mixture was filtered and the isolated solid washed with additional water (200 mL). The resulting solid was dried under vacuum to afford the desired product **4** as an orange solid (0.24 g, 0.84 mmol, 81%).

MP 239 - 240 °C; **IR** (ν_{max}/cm^{-1}) 1694, 1597, 1381, 1127, 961, 894, 683, 648, 527; **^{1}H NMR** (400 MHz, $CDCl_3$) δ 10.25 (s, 4H), 8.74 (d, *J* = 1.5 Hz, 4H), 8.58 (t, *J* = 1.5 Hz, 2H); **^{13}C NMR** (101 MHz, $CDCl_3$) δ 190.28, 153.13, 138.36, 132.74, 128.81; **HRMS** (CI) calc. for $C_{16}H_{10}N_2O_4$ $[M+H]^+$ 295.0713, found 295.0720.

(1*E*,1'*E*,1''*E*,1'''*E*)-1,1',1'',1'''-(((*E*)-Diazene-1,2-diyl)bis(benzene-5,1,3-triyl))tetrakis(N-cyclohexylmethanimine) (A1)

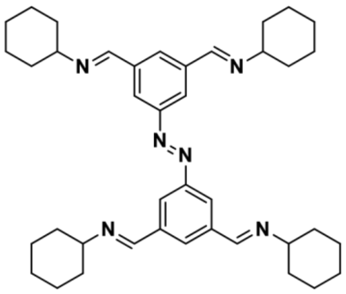

(*E*)-5,5'-(Diazene-1,2-diyl)diisophthalaldehyde **4** (22 mg, 0.08 mmol, 1.0 eq.) and cyclohexylamine (36 µl, 0.32 mmol, 4.0 eq.) were dissolved in DCM (10 mL). To the reaction mixture magnesium sulfate (0.5 g) was added and the reaction mixture was stirred at room temperature for 24 hours. The reaction mixture was then filtered and the DCM was removed *in vacuo*. The solid was triturated three times with hexane (~10 mL) which was then dried in a vacuum oven at 120 °C for 24 hours. Upon completion, the resulting solid was isolated as the desired product **A1** as an dark red solid (25 mg, 0.04 mmol, 53%).

MP 202 – 206 °C; **IR** (ν_{max}/cm^{-1}) 2925.67, 2852, 1642, 1595, 1448, 1345, 1236, 1214, 1154, 1073, 1026, 964, 892, 749, 692, 663, 624, 533, 497, 456; **^{1}H NMR** (400 MHz, $CDCl_3$) δ 8.44 (s, 4H), 8.32 (d, *J* = 1.5 Hz, 4H), 8.23 (s, 2H), 3.26 (m, 4H), 1.89 – 1.57 (m, 28H), 1.42 – 1.24 (m, 12H); **^{13}C NMR** (101 MHz, $CDCl_3$) δ; 157.58, 153.08, 138.28, 129.78, 124.22, 70.14, 34.48, 25.78, 24.91; **HRMS** (CI) calc. for $C_{40}H_{54}N_6$ [M+H]$^+$ 619.4483, found 619.4467.

2-(4-Nitrophenyl)-1,3-dioxolane (5)

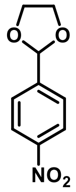

4-Nitrobenzaldehyde (5.2 g, 35 mmol, 1.0 eq.), ethylene glycol (2.06 mL, 37 mmol, 1.05 eq.), and *p*-toluenesulfonic acid (1.0 g, 5 mmol, 0.15 eq.) were dissolved in toluene (50 mL). The reaction mixture was heated at reflux for 16 hours, fitted with a pre-filled Dean-Stark apparatus. After cooling to room temperature, the toluene was removed *in vacuo*. Water was added to the solid mixture, and the solid collected by filtration under vacuum and washed with additional water (200 mL). The resulting solid was left to dry under continuous air flow to afford the desired product **5** as a yellow solid (6.6 g, 33.8 mmol, 96%) which was used without further purification.

^{1}H NMR (400 MHz, $CDCl_3$) δ 8.23 (d, *J* = 8.8 Hz, 2H), 7.65 (d, *J* = 8.5 Hz, 2H), 5.89 (s, 1H), 4.14 – 4.05 (m, 4H); **^{13}C NMR** (101 MHz, $CDCl_3$) δ 148.54, 145.08, 127.55, 123.71, 102.37, 65.61; Data in accordance with literature values.[25]

(*E*)-1,2-Bis(4-(1,3-dioxolan-2-yl)phenyl)diazene (6)

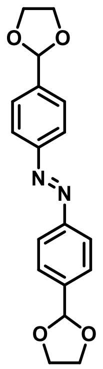

2-(4-Nitrophenyl)-1,3-dioxolane **5** (1.0 g, 5 mmol, 1.0 eq.), sodium hydroxide (0.8 g, 20 mmol, 4.0 eq.), and zinc dust (0.6 g, 10 mmol, 2.0 eq.) were dissolved in distilled ethanol (25 mL) and water (12.5 mL). The reaction mixture was heated to 100 °C for 20 hours. The reaction mixture was diluted with water and neutralized with 1 M HCl. The crude mixture was filtered to remove any insoluble material and washed with water. Ethanol was removed from the filtrate *in vacuo*. Chloroform (100 mL) and water (100 mL) was added to the remaining solution. The filtrate was separated with chloroform (3x100 mL) and the organic layers combined. The combined organic layers were dried (Na_2SO_4) and concentrated *in vacuo*. The crude product was purified by column chromatography (gradient petroleum ether/EtOAc 0-40%) and the orange band collected. This afforded the desired product **6** as an orange solid (0.46 g, 1.4 mmol, 56%).

MP 146 - 148 °C; **IR** (ν_{max}/cm^{-1}) 2895, 2847, 1385, 1218, 1073, 1011, 975, 939, 834, 695, 594, 529; **^{1}H NMR** (400 MHz, $CDCl_3$) δ 7.94 (d, *J* = 8.5 Hz, 4H), 7.63 (d, *J* = 8.3 Hz, 4H), 5.90 (s, 2H), 4.30 – 3.95 (m, 8H); **^{13}C NMR** (101 MHz, $CDCl_3$) δ 153.23, 140.89, 127.42, 123.08, 103.38, 65.53; **HRMS** (ES+) calc. for $C_{18}H_{18}N_2O_4$ [M+H]$^+$ 327.1339, found 327.1347.

(*E*)-4,4'-(Diazene-1,2-diyl)dibenzaldehyde (7)

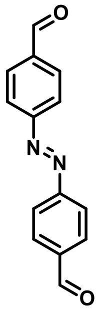

(*E*)-1,2-Bis(4-(1,3-dioxolan-2-yl)phenyl)diazene **6** (0.15 g, 0.47 mmol, 1.0 eq.) and *p*-toluenesulfonic acid (0.008 g, 0.05 mmol, 0.1 eq.) were dissolved in a 3:1 mix of acetone/water (10 mL). The reaction mixture was heated to 50 °C for 18 hours. After cooling to room temperature, the acetone was removed *in vacuo*. The mixture was filtered and the isolated solid washed with additional water (100 mL). The resulting solid was dried under vacuum to afford the desired product **7** as an orange solid (0.11 g, 0.47 mmol, quant.).

MP 234 - 235 °C; **IR** (ν_{max}/cm^{-1}) 1734, 1607, 1476, 1433, 1340, 1225, 881, 798, 656, 560, 539; **^{1}H NMR** (400 MHz, $CDCl_3$) δ 10.13 (s, 2H), 8.10 (d, *J* = 8.7 Hz, 4H), 8.07 (d, *J* = 8.7 Hz, 4H); **^{13}C NMR** (101 MHz, $CDCl_3$) δ 191.49, 155.59, 138.07, 130.75, 123.73; **HRMS** (CI) calc. for $C_{14}H_{10}N_2O_2$ [M+H]$^+$ 239.0815, found 239.0823.

(1*E*,1'*E*)-1,1'-(((*E*)-Diazene-1,2-diyl)bis(4,1-phenylene))bis(N-cyclohexylmethanimine) (A2)

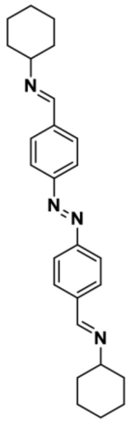

(*E*)-4,4'-(Diazene-1,2-diyl)dibenzaldehyde **7** (20 mg, 0.08 mmol, 1.0 eq.) and cyclohexylamine (18 µl, 0.16 mmol, 2.0 eq.) were dissolved in DCM (10 mL). To the reaction mixture magnesium sulfate (0.5 g) was added and the reaction mixture was stirred at room temperature for 24 hours. The reaction mixture was then filtered and the DCM was removed *in vacuo*. The solid was triturated three times with hexane (~10 mL) which was then dried in a vacuum oven at 120 °C for 24 hours. Upon completion, the resulting solid was isolated as the desired product **A2** as an orange solid (23 mg, 0.06 mmol, 71%).

MP 206 - 211 °C; **IR** (ν_{max}/cm^{-1}) 2923, 2850, 1637, 1593, 1449, 1303, 1294, 1214, 1163, 1148, 1070, 1011, 886, 843, 752, 562, 530, 500; **^{1}H NMR** (400 MHz, $CDCl_3$) δ ^{1}H NMR (400 MHz, $CDCl_3$) δ 8.38 (s, 2H), 7.96 (d, *J* = 8.5 Hz, 4H), 7.88 (d, *J* = 8.5 Hz, 4H), 3.34 – 3.13 (m, 2H), 1.91 – 1.81 (m, 4H), 1.79 – 1.75 (m, 4H), 1.72 – 1.62 (m, 4H), 1.49 – 1.16 (m, 8H); **^{13}C NMR** (101 MHz, $CDCl_3$) δ 157.97, 153.79, 139.10, 129.02, 123.37, 70.36, 34.48, 25.77, 24.92; **HRMS** (CI) calc. for $C_{26}H_{32}N_4$ $[M+H]^+$ 401.2700, found 401.2709.

3. Cage Screening

Supplementary Table 1 Preliminary cage synthesis screen: varying the reaction conditions by changing the concentration and solvent for the reaction of (*E*)-5,5'-(diazene-1,2-diyl)diisophthalaldehyde **4** with (1*R*,2*R*)-diaminocyclohexane, forming azobenzene-based covalent cage (**ACC-1**).

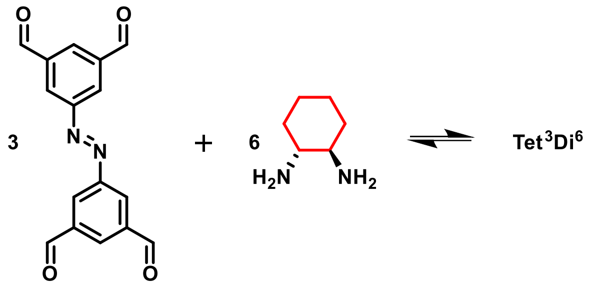

Entry	Concentration (mg/mL)	Solvent	Precipitate	Aldehyde SM in NMR	HRMS
1	0.5	DCM	✗	✗	✓
2	0.25	DCM	✗	✗	✓
3	0.5	$CDCl_3$	✗	✗	✓
4	0.5	$CDCl_3$:CD_3OD	✗	✗	✓
5	0.001	$CDCl_3$:CD_3OD	✗	✗	✓

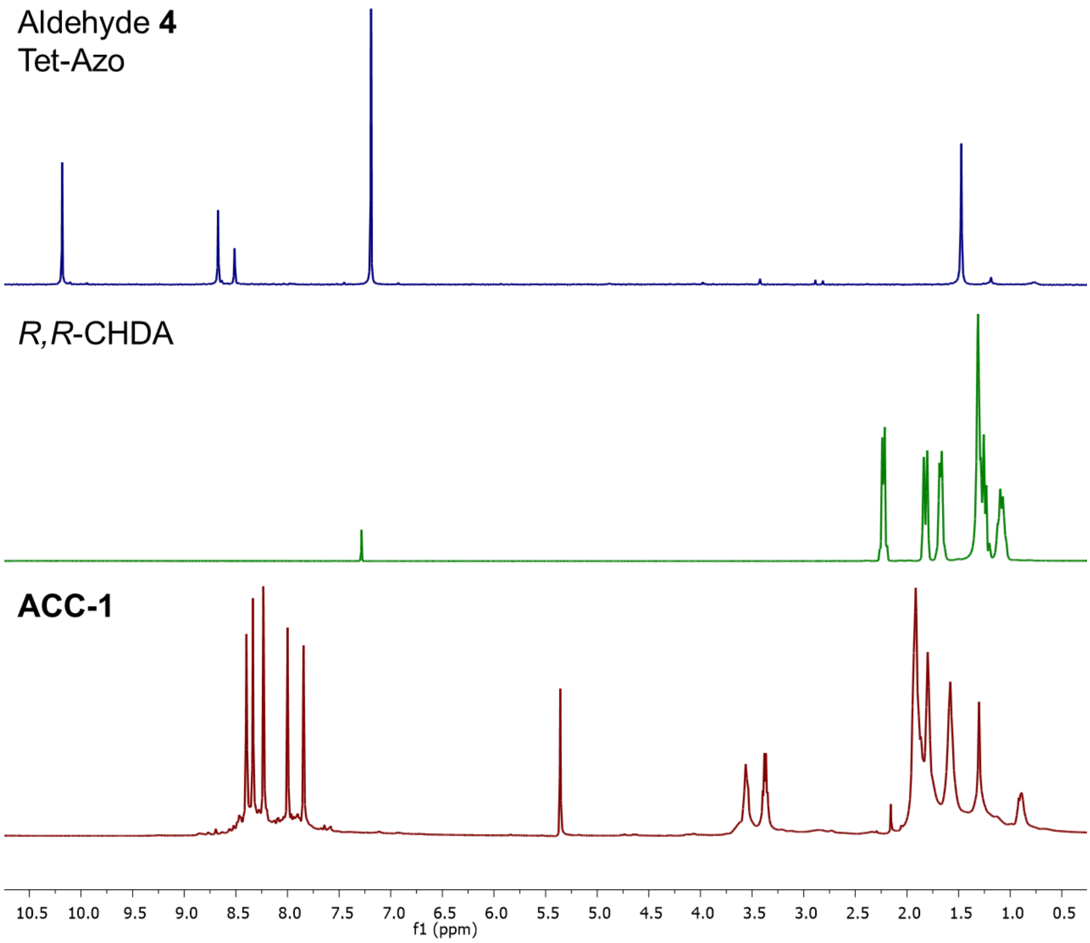

Supplementary Figure 1 Stacked ^{1}H NMR spectra of **ACC-1** and its precursors – tetraaldehyde **4** (top, blue, $CDCl_3$), *R,R*-CHDA (middle, green, $CDCl_3$), and **ACC-1** (bottom, red, CD_2Cl_2).

Supplementary Table 2 A selection of diamines (**DA 1-7**) used in the synthetic screen for cage formation with (*E*)-5,5'-(Diazene-1,2-diyl)diisophthalaldehyde **4** with their corresponding HRMS and size of cage formed. [1]No Trace of $[M+H]^+$ for $\mathbf{Tet^3Di^6}$ or $\mathbf{Tet^2Di^4}$ cage species.

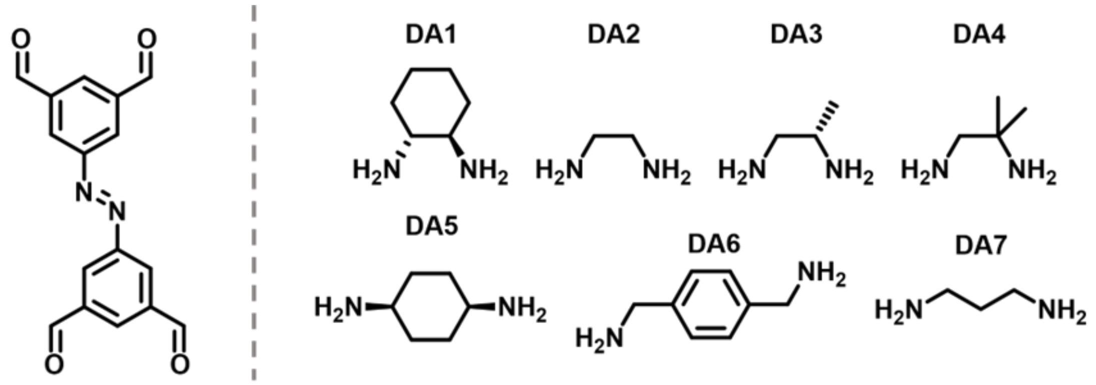

Diamine	Precipitate	Aldehyde SM in NMR	HRMS Calc.	HRMS Found	Cage formed
DA1	×	×	1351.7669	1351.7775	Tet^3Di^6
DA2	✓	×	1027.4852	1027.4839	Tet^3Di^6
DA3	✓	✓	1111.5791	1111.5745	Tet^3Di^6
DA4	×	×	1195.6730	1195.6615	Tet^3Di^6
DA5	✓	×	1519.9547	None[1]	No trace
DA6	✓	×	1453.4382	None[1]	No trace
DA7	✓	×	741.3885	741.3832	Tet^2Di^4

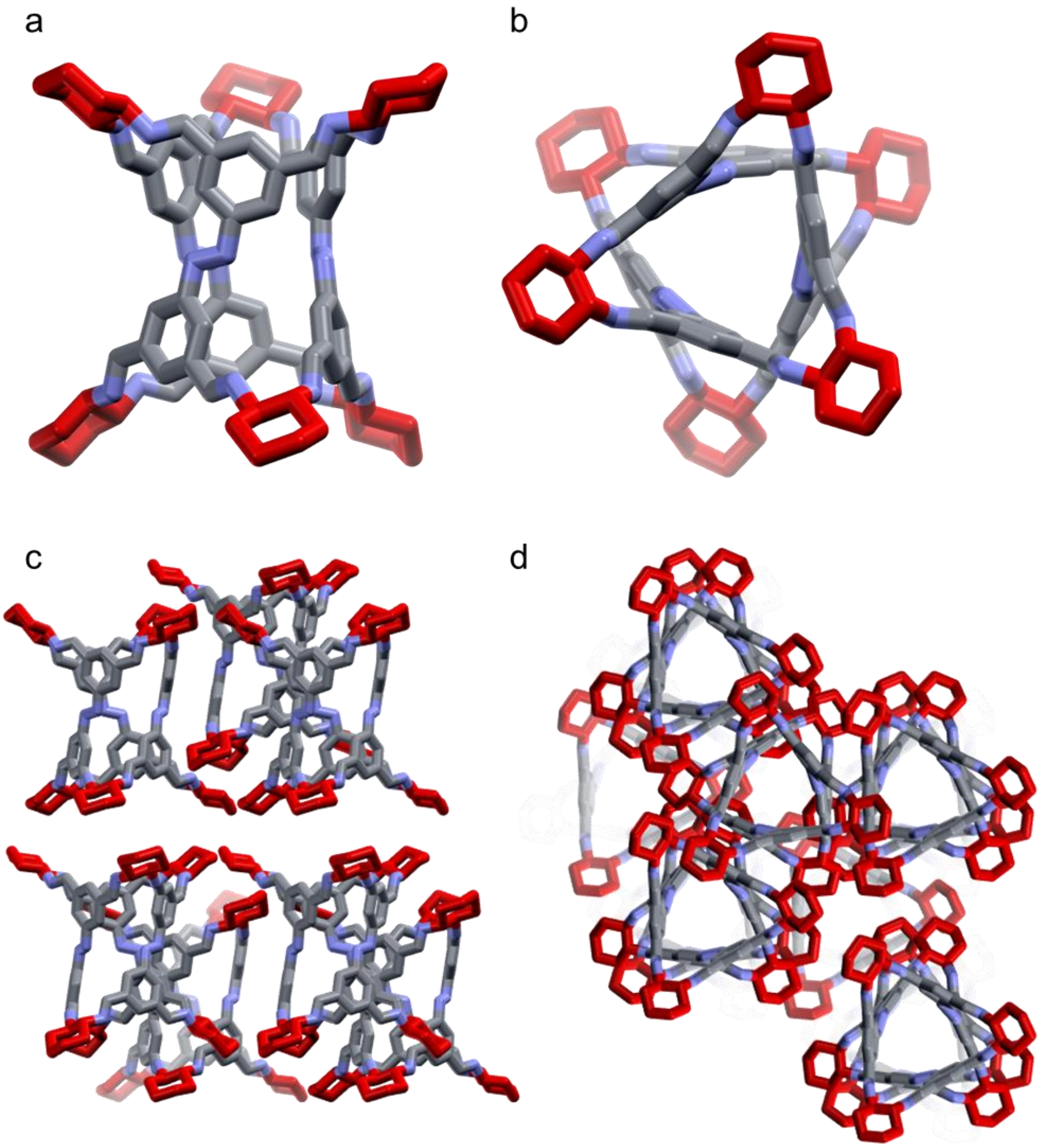

Supplementary Figure 2 Single-crystal structures of azobenzene derived cage **ACC-1**, where CHDA is highlighted in red: a) Side-on view; b) top-down view; c) side-on view of the offset azo-to-azo packing between stacks; d) top-down view of window-to-window stacks. Hydrogen atoms have been removed for clarity.

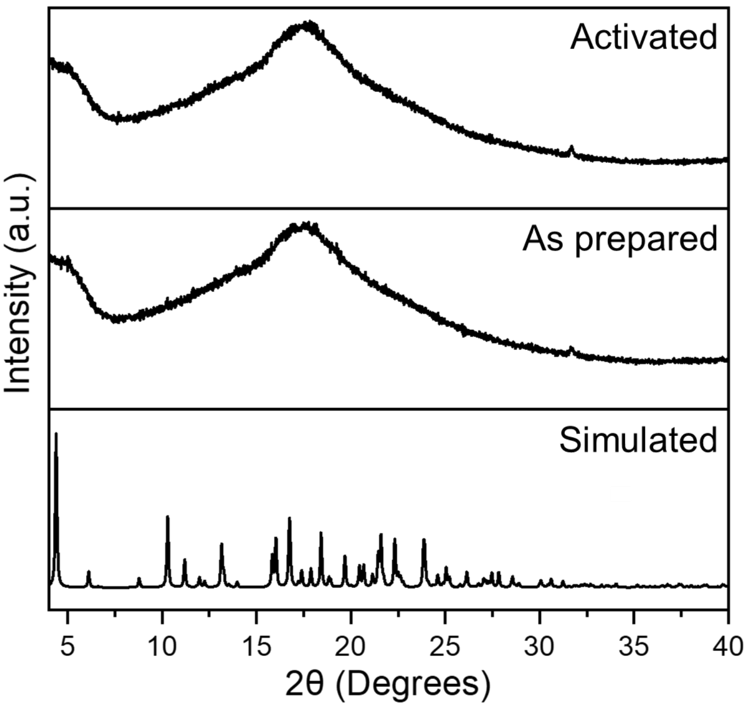

Supplementary Figure 3 PXRD patterns of **ACC-1**, with patterns from the activated sample which was heated in a vacuum oven at 80 °C for 15 hours, top, as prepared from the solvent swap with acetonitrile, middle, and simulated powder pattern from the SCXRD data, bottom.

Supplementary Table 3 Triamines (**TA 1-3**) used in the synthetic screen for cage formation with (*E*)-4,4'-(diazene-1,2-diyl)dibenzaldehyde, **7**, with their corresponding HRMS and size of cage formed.

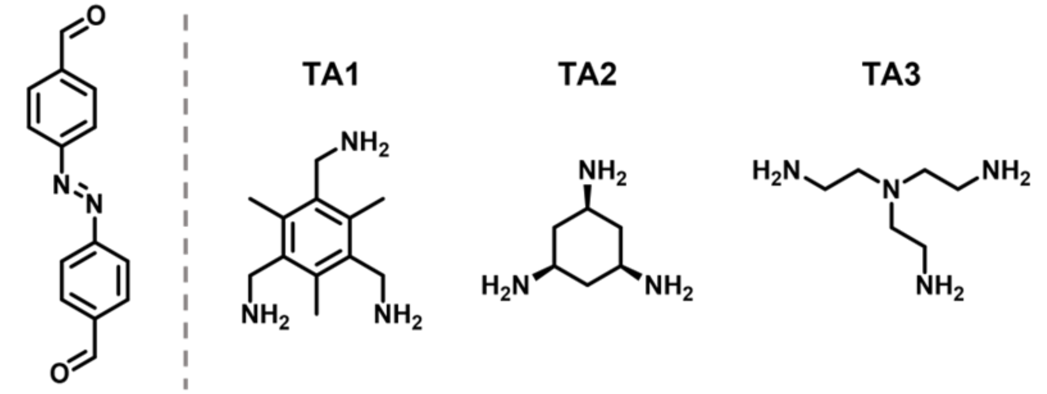

Diamine	Precipitate	Aldehyde SM in NMR	HRMS Calc.	HRMS Found	Cage formed
TA1	✓	×	1021.5137	1021.5194	Tri^2Di^3
TA2	×	✓	865.4198	865.4280	Tri^2Di^3
TA3	×	×	899.4729	899.4787	Tri^2Di^3

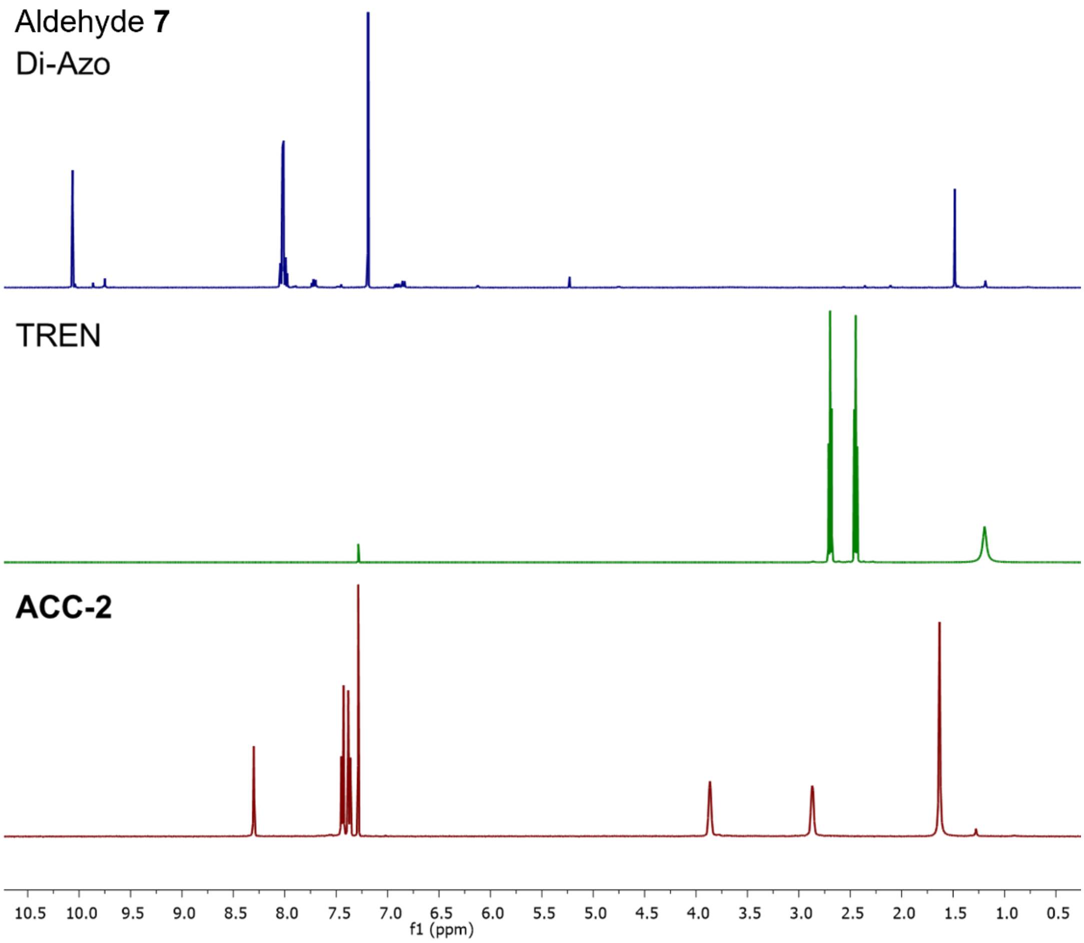

Supplementary Figure 4 Stacked ^{1}H NMR spectra of **ACC-2** and its precursors – dialdehyde **7** (top, blue, $CDCl_3$), tris(2-aminoethyl)amine (TREN) (middle, green, $CDCl_3$), and **ACC-2** (bottom, red, $CDCl_3$).

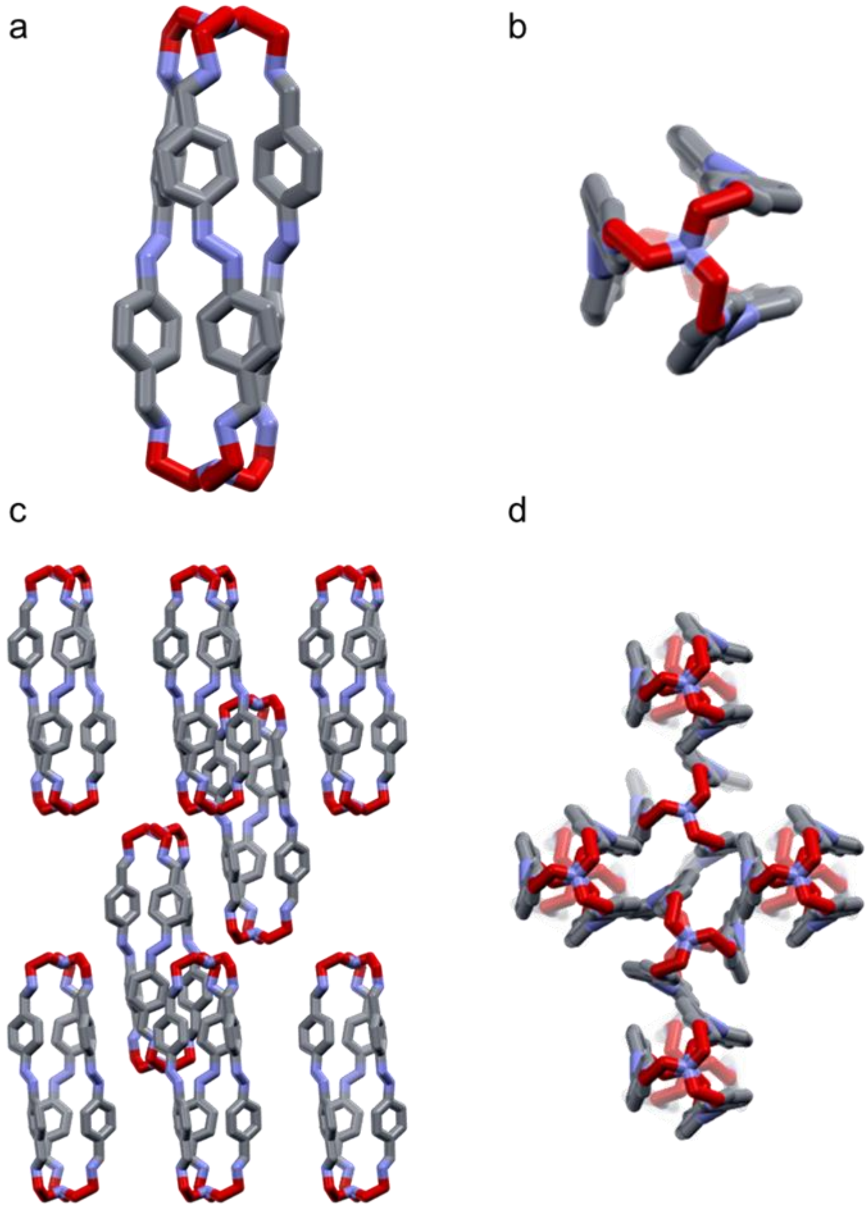

Supplementary Figure 5 Single-crystal structures of azobenzene-derived cage **ACC-2**, where tris(2-aminoethyl)amine (TREN) is highlighted in red: a) Side-on view; b) top-down view; c) side-on view of the staggered window-to-window packing between stacks; d) top-down view of the stacks. Hydrogen atoms have been removed for clarity.

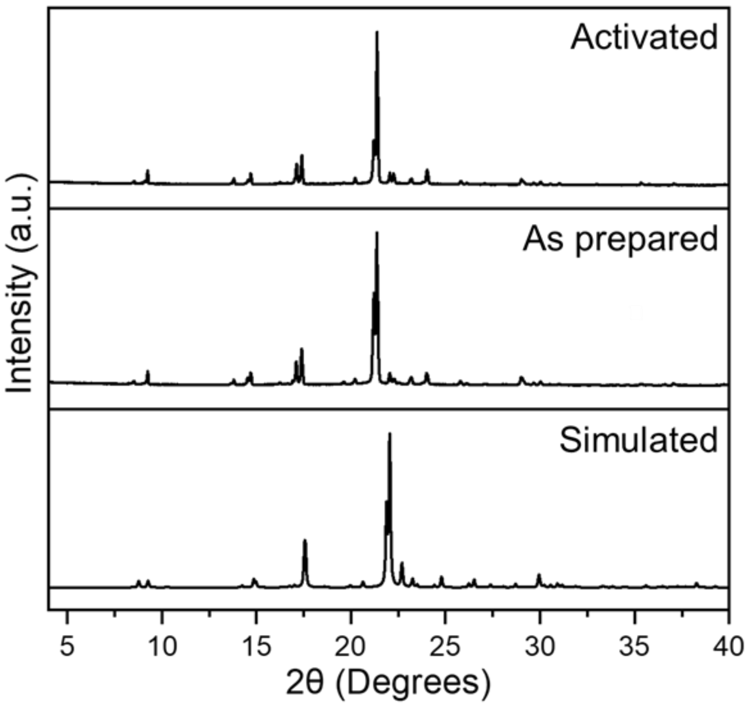

Supplementary Figure 6 PXRD patterns of **ACC-2**, with patterns from the activated sample which was heated in a vacuum oven at 80 °C for 15 hours, top, as prepared from the solvent swap with hexane, middle, and simulated powder pattern from the SCXRD data, bottom

Supplementary Table 4 Single crystal refinement details for **ACC-1** and **ACC-2**. [1]Absolute configuration determined from synthetic details.

Molecule	**ACC-1**	**ACC-2**
λ [Å]	Mo-Kα	Mo-Kα
Collection Temperature	100 K	100 K
Formula	$C_{84}H_{90}N_{18}$, 1.5(CH_2Cl_2), 1.5(C_2H_6O)	$C_{54}H_{54}N_{14}$, CH_2Cl_2
Mr [g mol^{-1}]	1548.22	984.03
Crystal Size [mm]	0.23 × 0.21 × 0.09	0.28 × 0.23 × 0.14
Crystal System	trigonal	trigonal
Space Group	*R*32	$R\bar{3}c$
a [Å]	16.9884(7)	12.4324(11)
c [Å]	59.610(4)	57.227(5)
V [Å^3]	14898.9(15)	7660.2(15)
Z	6	6
D_{calcd} [g cm^{-3}]	1.035	1.275
μ [mm^{-1}]	0.142	0.086
F(000)	4932	3090
2θ range [°]	3.088–41.626	4.042–43.986
Reflections collected	49104	14909
Independent reflections, R_{int}	3478, 0.1531	1052, 0.0330
Obs. Data [$I > 2\sigma(I)$]	3042	818
Data / restraints/ parameters	3478/12/345	1052/154/185
R_1 ($I > 2\sigma(I)$)	7.27%	5.98%
R_1 (all data)	8.58%	7.41%
wR_2 (all data)	17.17%	16.80%
Flack parameter[1]	-0.07(10)	
Goodness-of-fit (or S) on F^2	1.077	1.096
Largest difference peak and hole (or Dr_{max} and Dr_{min}) [e A^{-3}]	0.220 and -0.254	0.305 and -0.197
CCDC	2282356	2282355

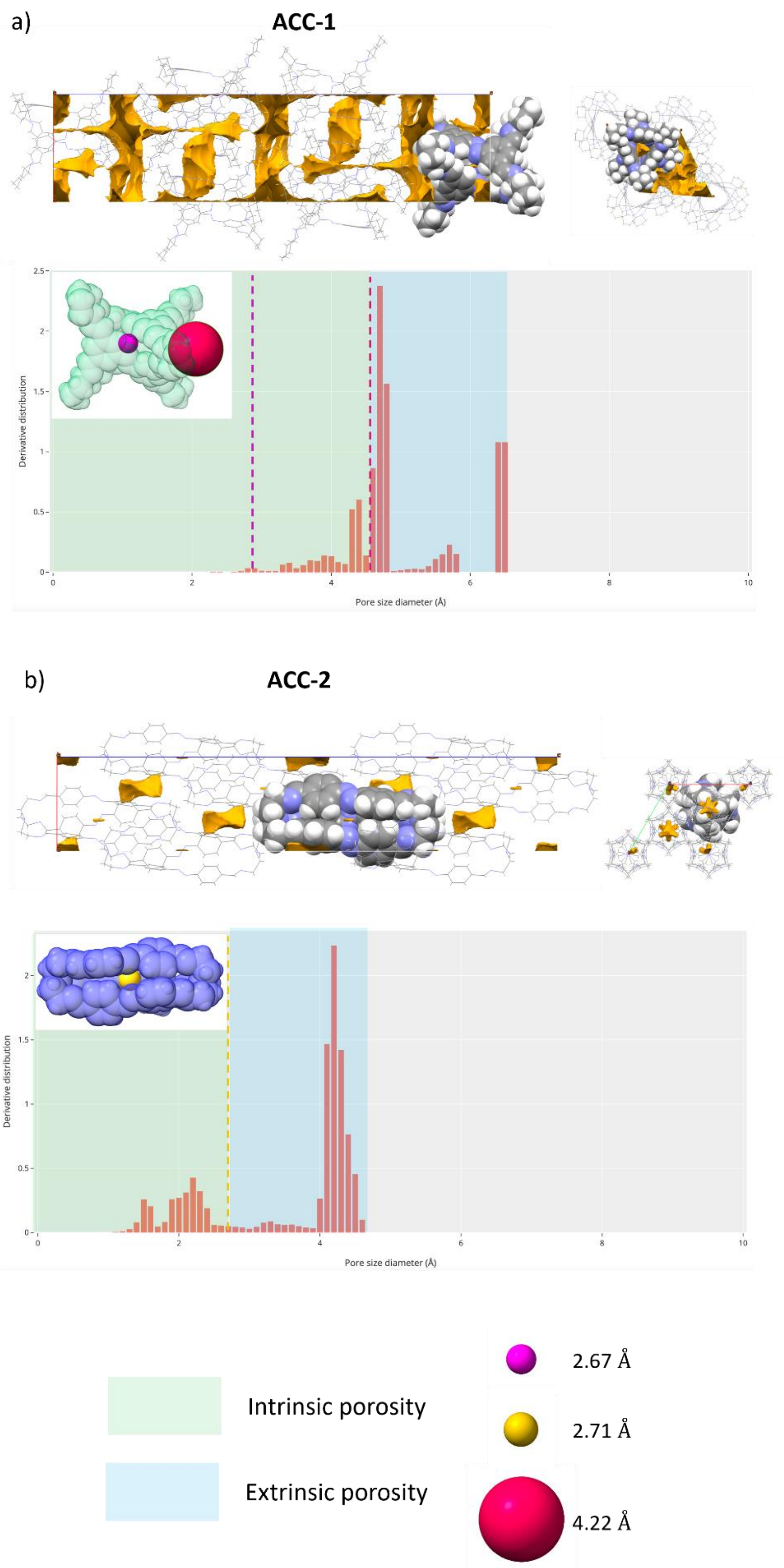

Supplementary Figure 7 Porosity analysis of experimental crystal structures with contact surfaces using a 1.2 Å probe – inside contact surfaces shown in yellow and outside contact surfaces shown in brown. Ball and stick representations have been used except for a single cage molecule where the

atoms are shown in hard sphere representations to highlight pore structures. Simulated pore size distributions with a probe size of 1.2 Å are also shown, with intrinsic pore cavities illustrated as coloured spheres in molecular representations of individual cages at 50% transparency. (a) **ACC-1** shown in lattice direction pointing towards lattice vector b (left) and lattice vector c (right); (b) **ACC-2** shown in lattice direction pointing towards lattice vector b (left) and lattice vector c (right).

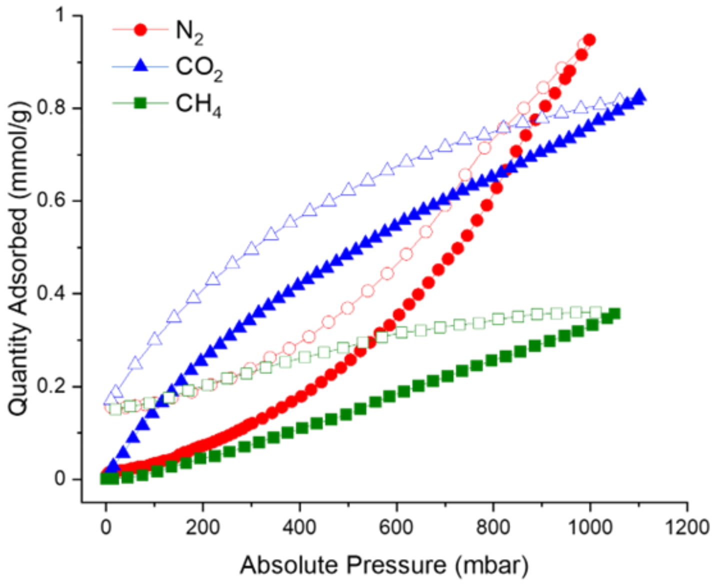

Supplementary Figure 8 Gas sorption isotherms adsorption (filled) and desorption (empty) of **ACC-1** where red circles – N_2 (77 K), blue triangles - CO_2 (273 K), and green squares – CH_4 (273 K).

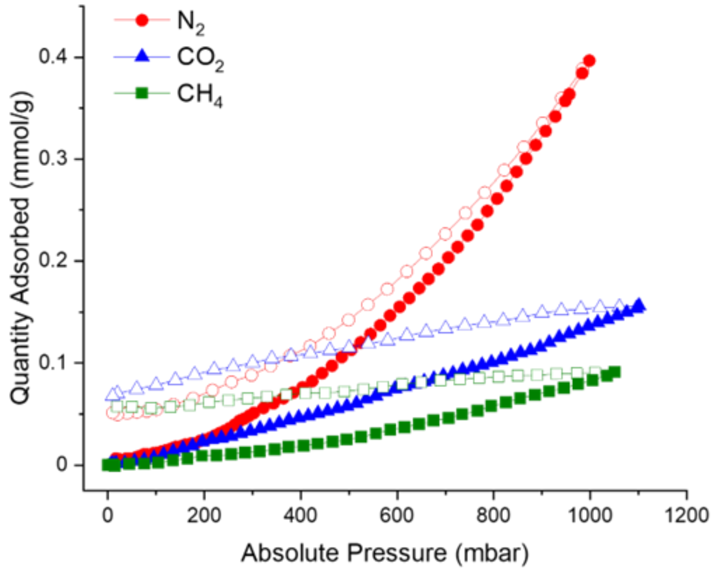

Supplementary Figure 9 Gas sorption isotherms adsorption (filled) and desorption (empty) of **ACC-2** where red circles – N_2 (77 K), blue triangles - CO_2 (273 K), and green squares – CH_4 (273 K).

4. Photophysical Properties of Azobenzene-Derived Organic Cages by UV-Vis

For photoswitches **ACC-1** and **A1**, thermal half-lives at 25 °C were extrapolated from Eyring plots[26] at elevated temperatures. The error in extrapolation was calculated by linear regression analysis. Thermal half-lives of **ACC-2** and **A2** at 25 °C were obtained directly owing to their shorter thermal isomerization times. Eyring plots were generated for all photoswitches, allowing the determination of their activation enthalpy and entropy.

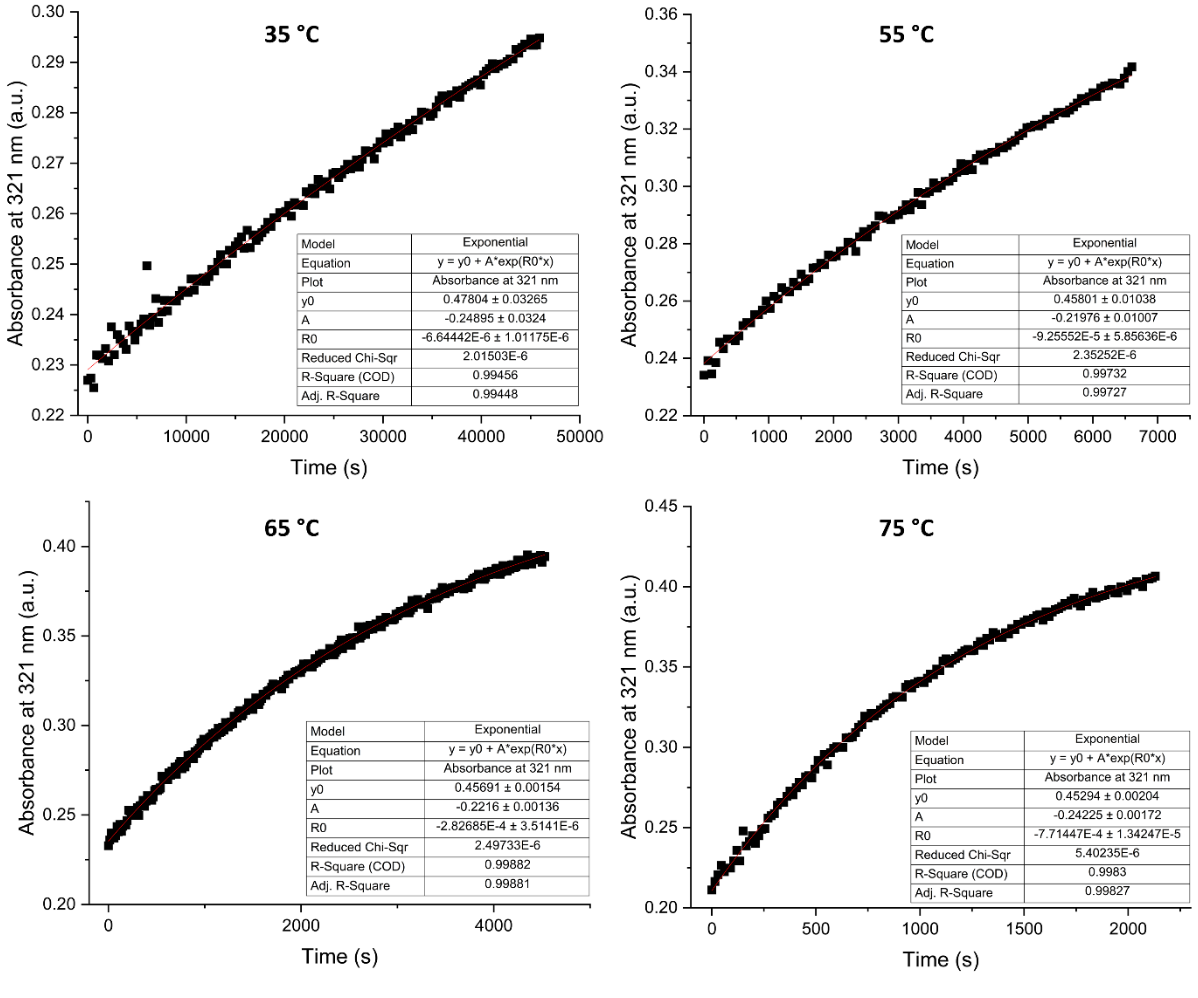

Supplementary Figure 10 Thermal isomerization kinetics of **ACC-1** in dry DCE with a concentration of 10 µM over time for varying temperatures and the corresponding exponential fit line, where**:** at a) 35 °C / 308.15 K, b) 55 °C / 328.15 K, c) 65 °C / 338.15 K, and d) 75 °C / 348.15 K.

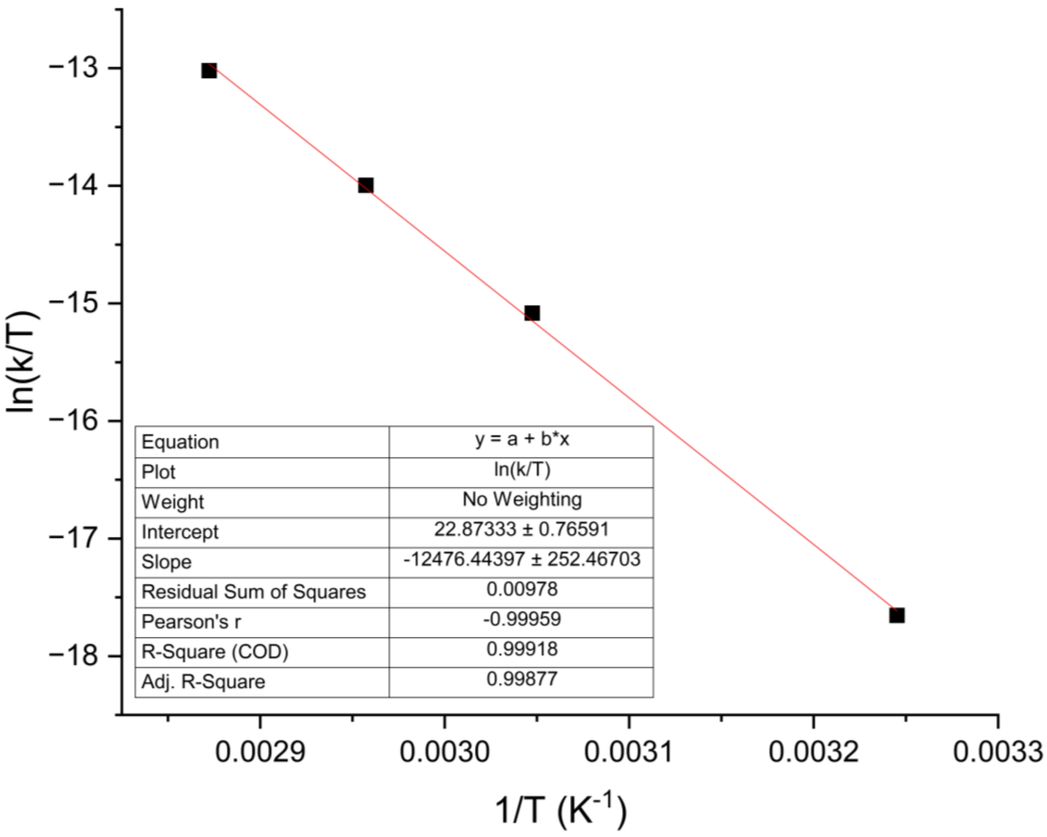

Supplementary Figure 11 Eyring plot of **ACC-1** determined from the thermal isomerisation kinetic experiments. Extrapolation of the best fit line allowed the thermal half-life to be calculated as 110 ± 10 hours. The activation enthalpy ($\Delta H^{\ddagger}$ = 104 ± 2 kJ mol^{-1}) and entropy ($\Delta S^{\ddagger}$ = -7 ± 6 J K^{-1} mol^{-1}) were calculated from the slope and intercept respectively.

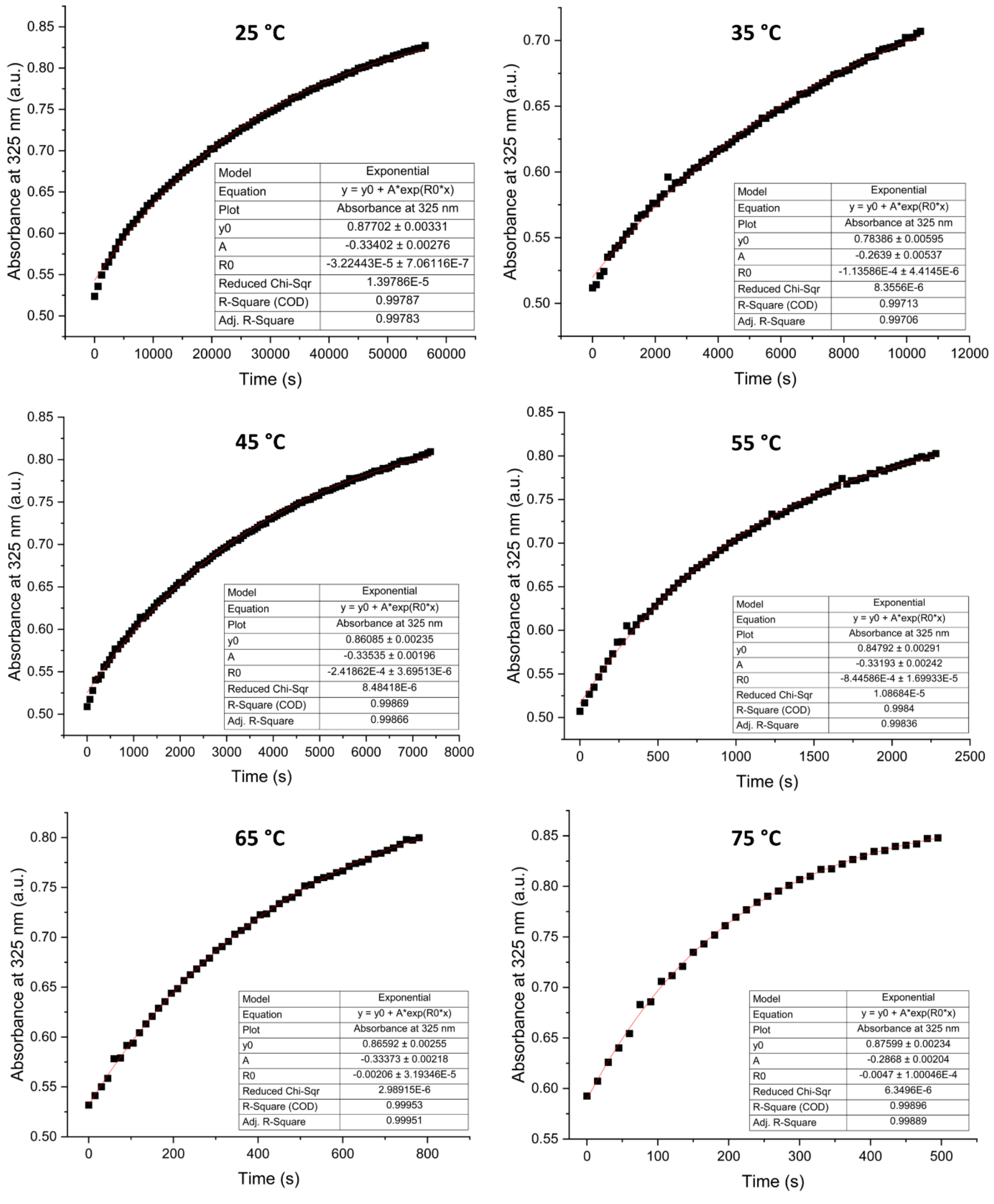

Supplementary Figure 12 Thermal isomerization kinetics of **ACC-2** in dry DCE with a concentration of 10 µM over time for varying temperatures and the corresponding exponential fit line, where**:** at a) 25 °C / 298.15 K, b) 35 °C / 308.15 K, c) 45 °C / 318.15 K, d) 55 °C / 328.15 K, e) 65 °C / 338.15 K, and f) 75 °C / 348.15 K. The thermal half-life of **ACC-2** was taken directly from measurements at 25 °C to give a value of 6.0 ± 0.1 hours.

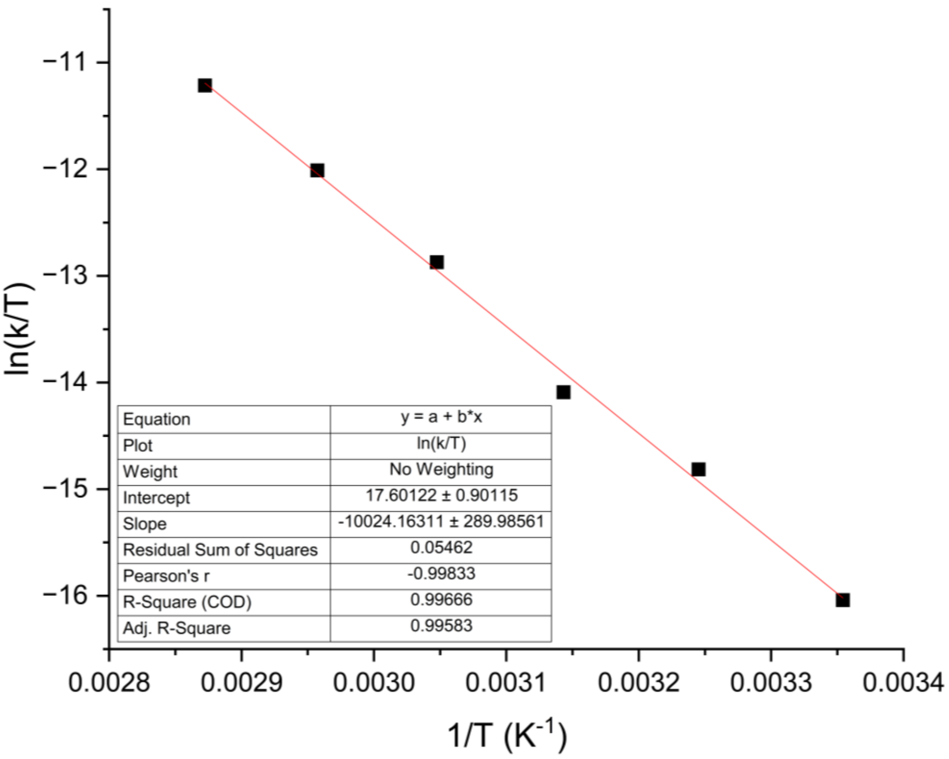

Supplementary Figure 13 Eyring plot of **ACC-2** determined from the thermal isomerisation kinetic experiments. The activation enthalpy ($\Delta H^{\ddagger}$ = 83 ± 2 kJ mol^{-1}) and entropy ($\Delta S^{\ddagger}$ = -51 ± 7 J K^{-1} mol^{-1}) were calculated from the slope and intercept respectively.

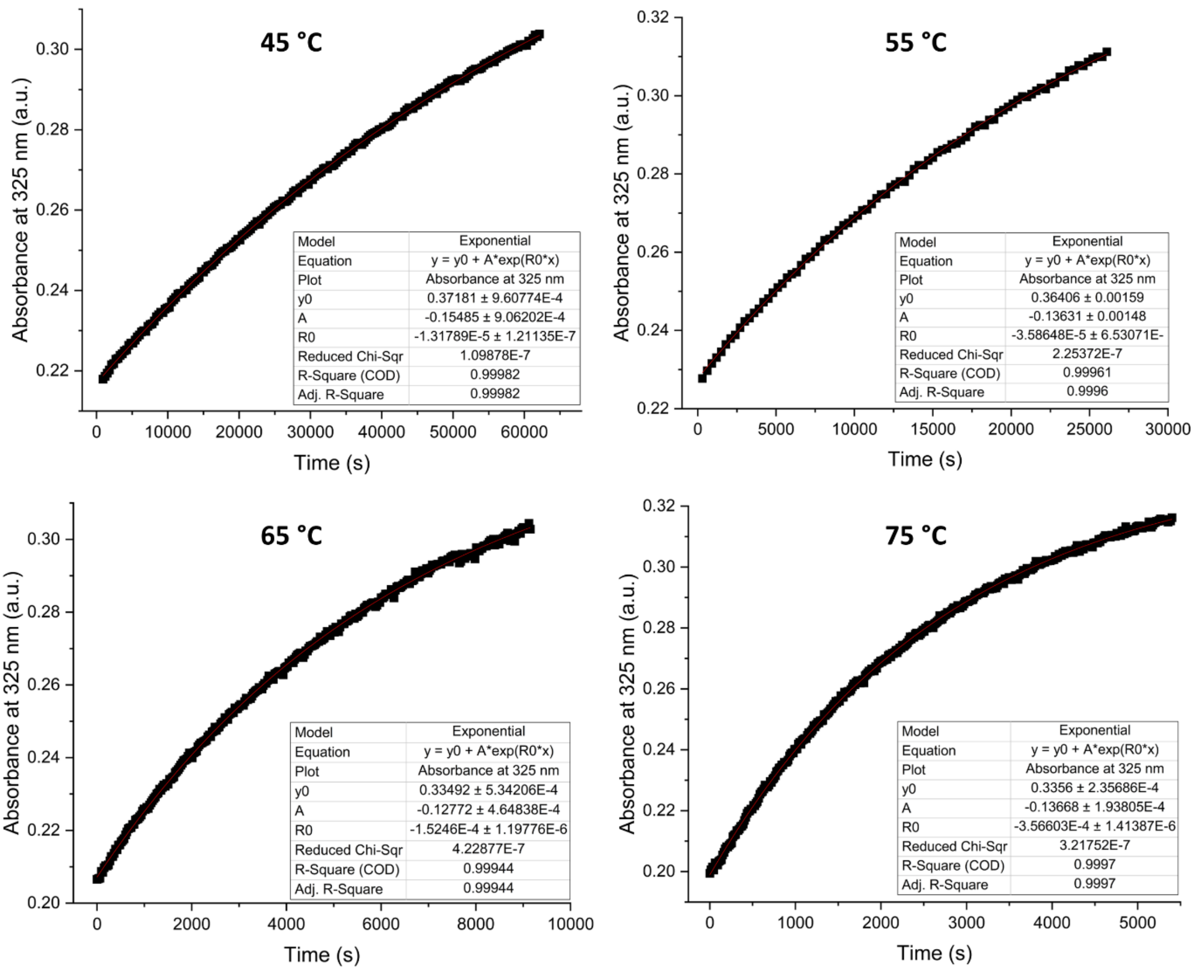

Supplementary Figure 14 Thermal isomerization kinetics of **A1** in dry DCE with a concentration of 30 µM over time for varying temperatures and the corresponding exponential fit line, where**:** at a) 45 °C / 318.15 K, b) 55 °C / 328.15 K, c) 65 °C / 338.15 K, and d) 75 °C / 348.15 K.

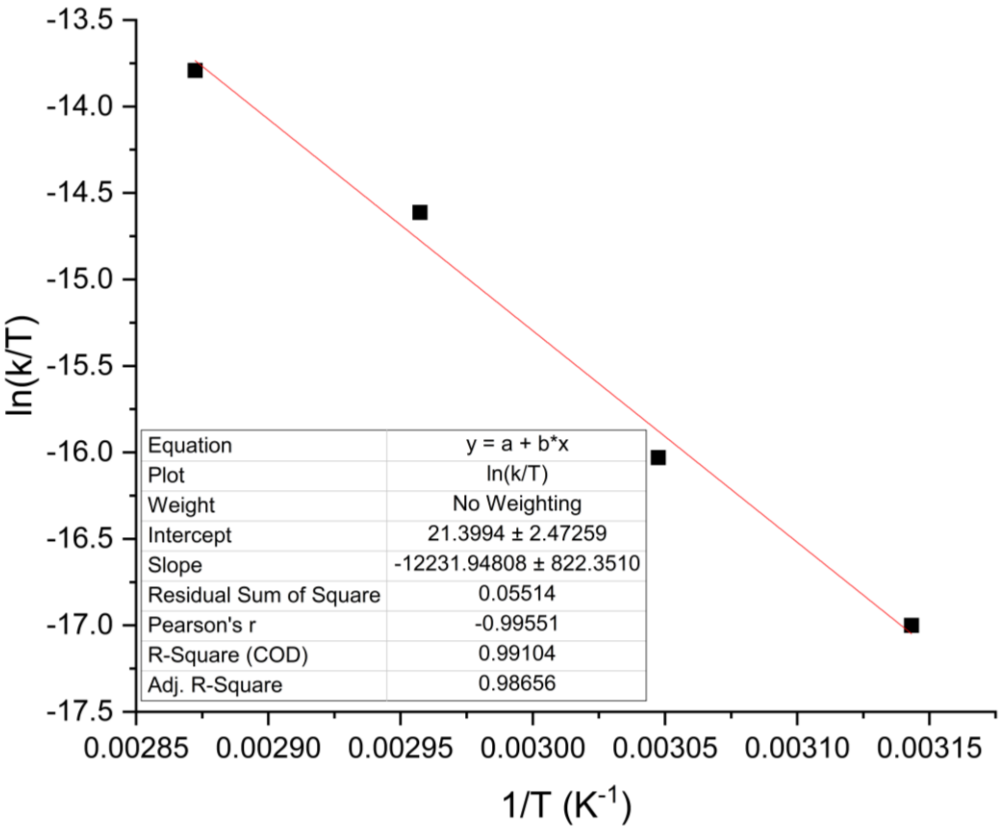

Supplementary Figure 15 Eyring plot of **A1** determined from the thermal isomerisation kinetic experiments. Extrapolation of the best fit line allowed the thermal half-life to be calculated as 220 ± 70 hours. The activation enthalpy ($\Delta H^{\ddagger}$ = 102 ± 7 kJ mol^{-1}) and entropy ($\Delta S^{\ddagger}$ = -20 ± 20 J K^{-1} mol^{-1}) were calculated from the slope and intercept respectively.

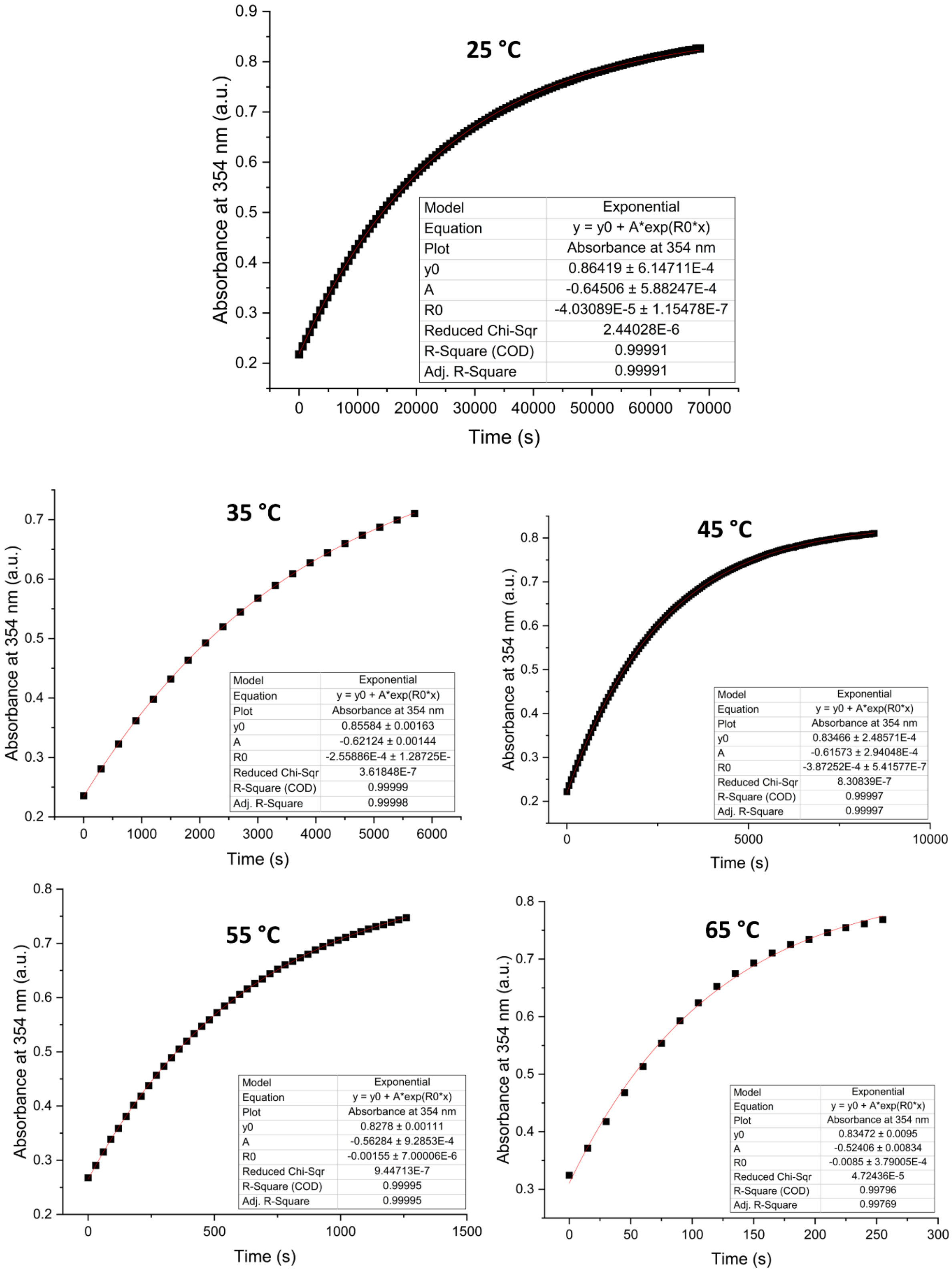

Supplementary Figure 16 Thermal isomerization kinetics of **A2** in dry DCE with a concentration of 30 µM over time for varying temperatures and the corresponding exponential fit line, where: at a) 25 °C / 298.15 K, b) 35 °C / 308.15 K, c) 45 °C / 318.15 K, d) 55 °C / 328.15 K, and e) 65 °C / 338.15 K. The thermal half-life of **A2** was taken directly from measurements at 25 °C to give a value of 4.78 ± 0.01 hours.

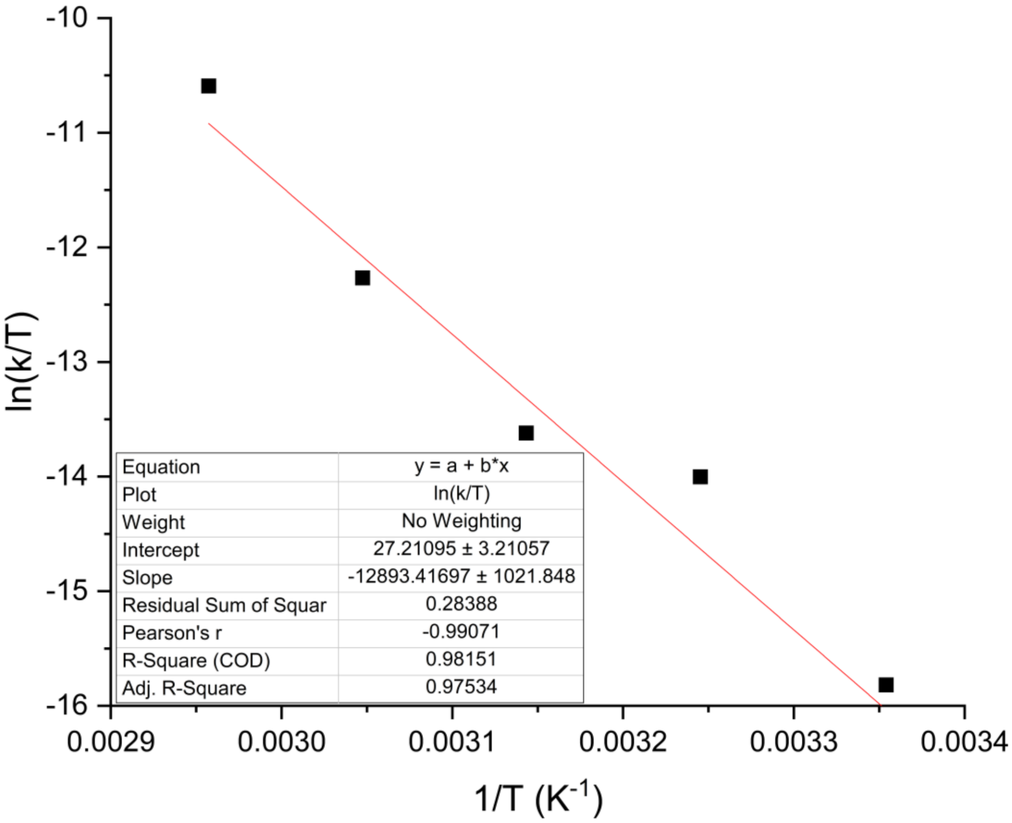

Supplementary Figure 17 Eyring plot of **A2** determined from the thermal isomerisation kinetic experiments. The activation enthalpy ($\Delta H^{\ddagger}$ = 107 ± 8 kJ mol^{-1}) and entropy ($\Delta S^{\ddagger}$ = 30 ± 30 J K^{-1} mol^{-1}) were calculated from the slope and intercept respectively.

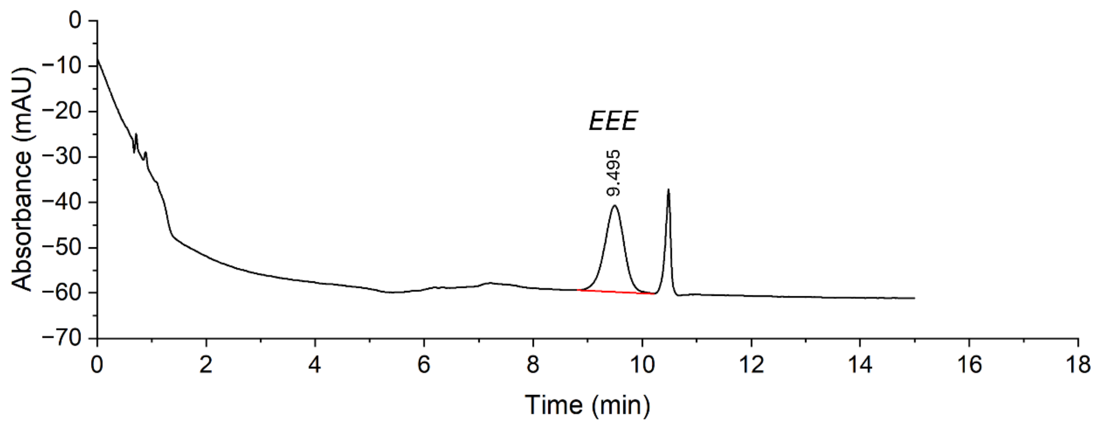

Supplementary Figure 18 HPLC trace at 230 nm of a dark sample of **ACC-1**.

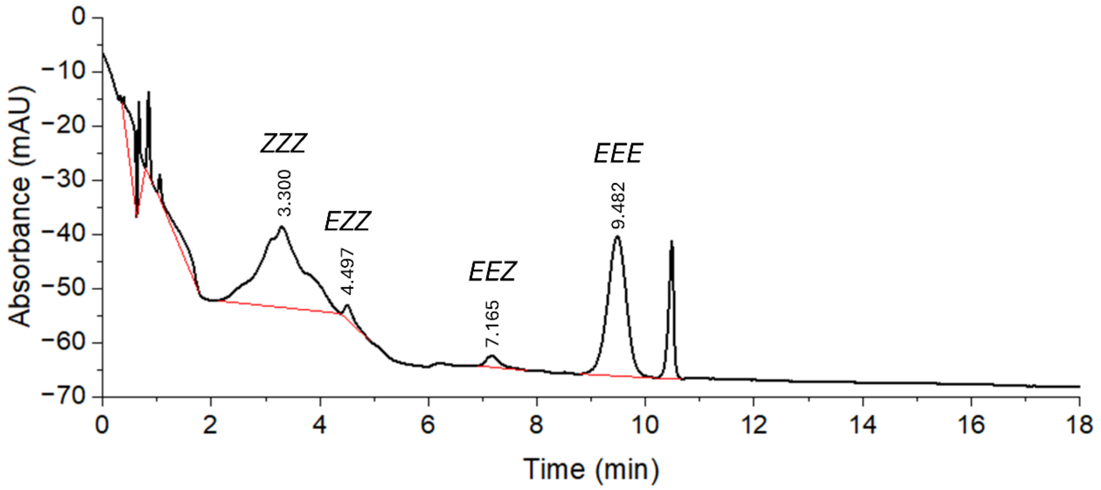

Supplementary Figure 19 HPLC trace at 230 nm of **ACC-1** after irradiation with 365 nm light for 60 min in an HPLC vial.

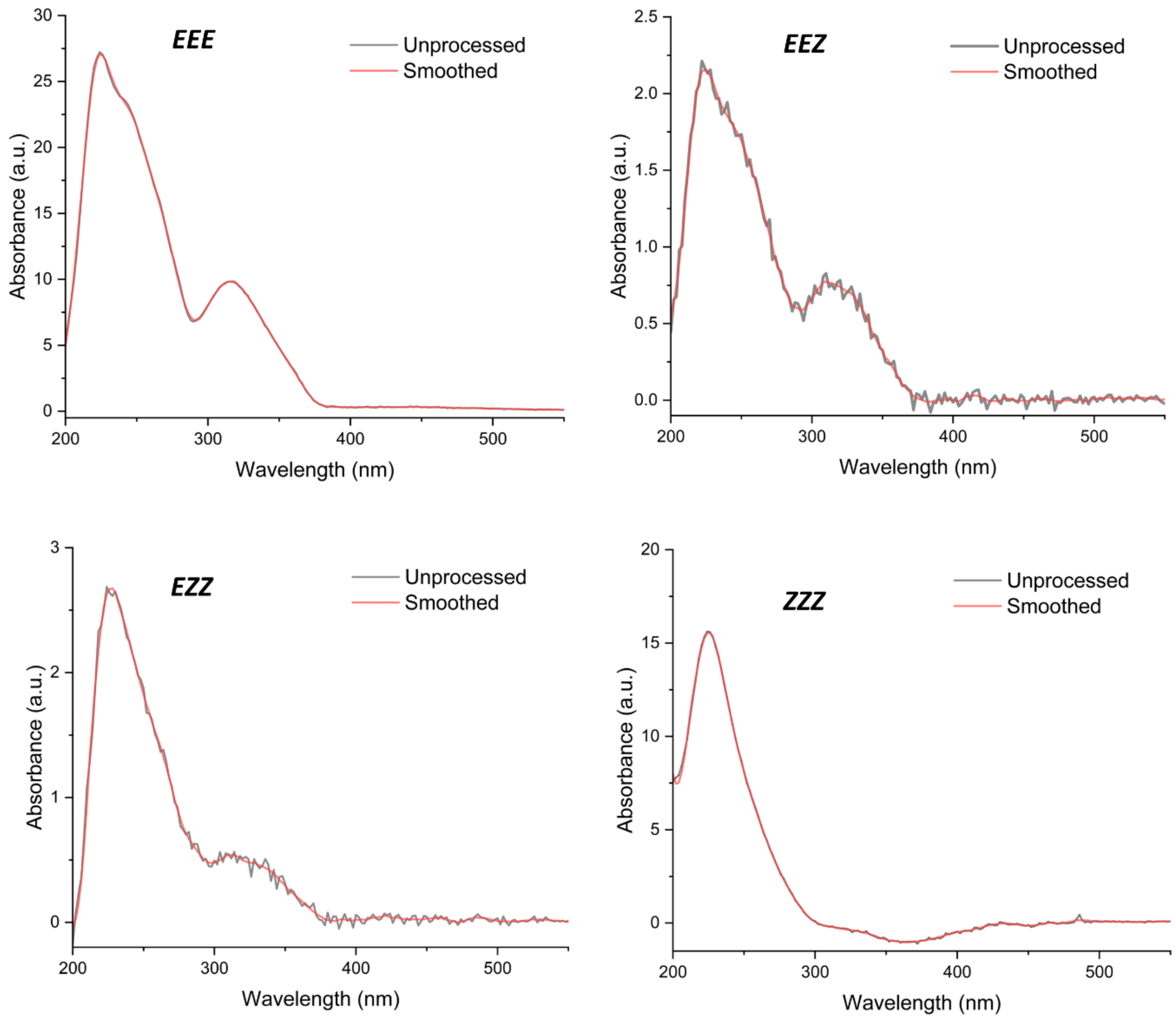

Supplementary Figure 20 UV–Vis spectra of individual photoisomers of **ACC-1** after irradiation with 365 nm light and separation by HPLC. *NB.* The negative absorbance in the spectrum for *ZZZ*-**ACC-1** is due to a baseline correction within the 4 min HPLC solvent gradient required for isomeric separation. This artifact is not present in the spectra for other isomers due to their > 4 min retention times, and subsequent isocratic elution.

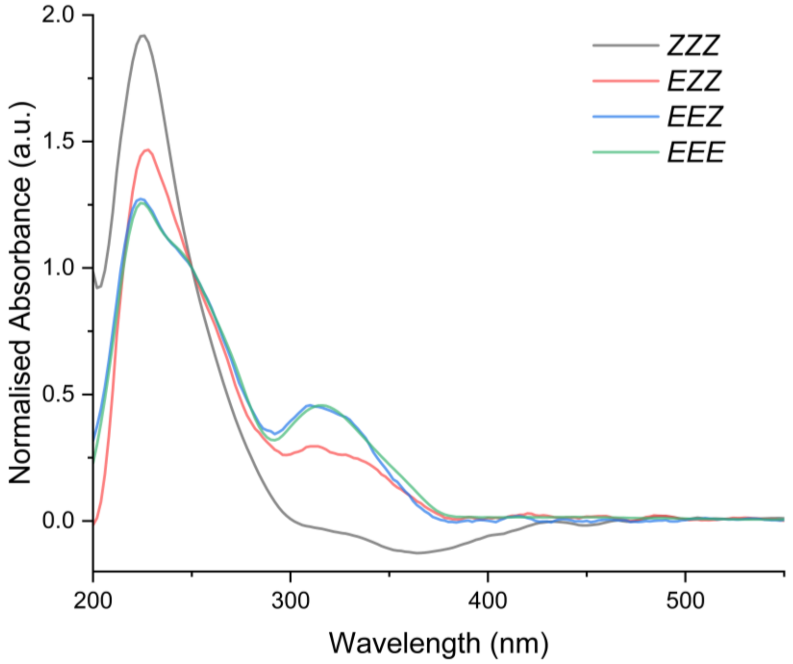

Supplementary Figure 21 Overlayed smoothed UV–Vis spectra of **ACC-1** photoisomers (Fig. S19), normalized to the isosbestic point at 250 nm. *NB.* The negative absorbance in the spectrum for *ZZZ*-**ACC-1** is due to a baseline correction within the 4 min HPLC solvent gradient required for isomeric separation. This artifact is not present in the spectra for other isomers due to their > 4 min retention times, and subsequent isocratic elution.

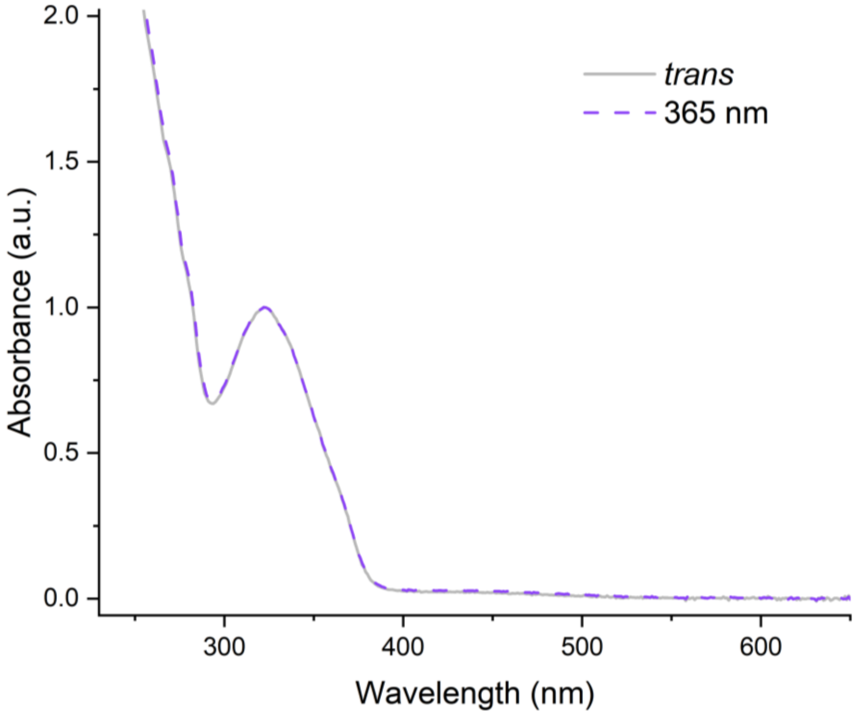

Supplementary Figure 22 UV-Vis absorbance spectra of **ACC-1** (dry 1,2-dichloroethane, 25 °C; normalised to the π–π* band) before and after 365 nm irradiation for 90 min as a finely dispersed powder – sample was irradiated as a solid prior to dissolving in dry 1,2-dichloroethane for subsequent UV-Vis analysis of the solution.

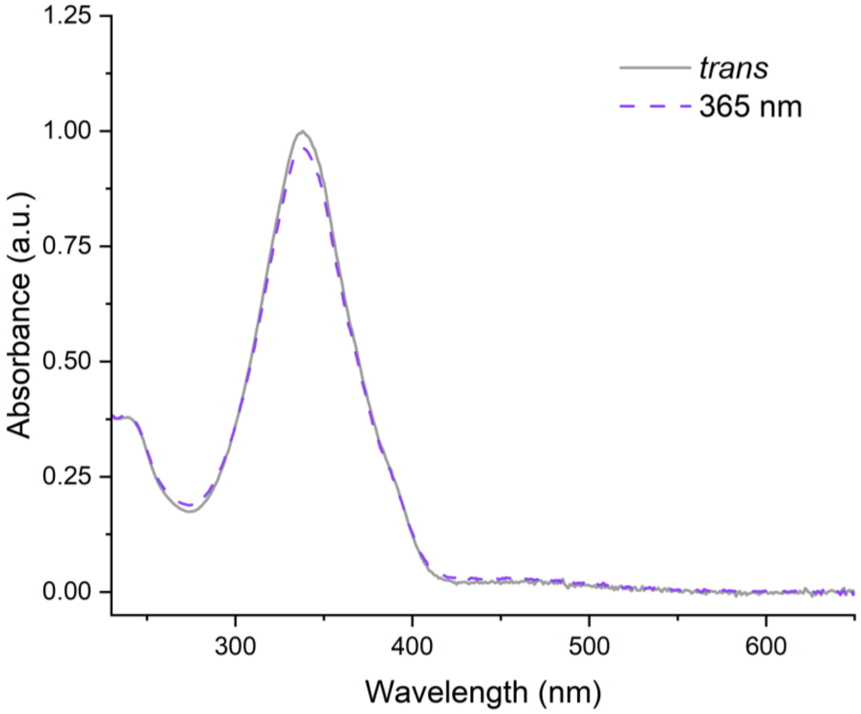

Supplementary Figure 23 UV-Vis absorbance spectra of **ACC-2** (dichloromethane, 25 °C; normalised to the 300 nm isosbestic point) before and after 365 nm irradiation for 90 min as a finely dispersed powder – sample was irradiated as a solid prior to dissolving in dry 1,2-dichloroethane for subsequent UV-Vis analysis of the solution.

5. Circular Dichroism of ACC-1

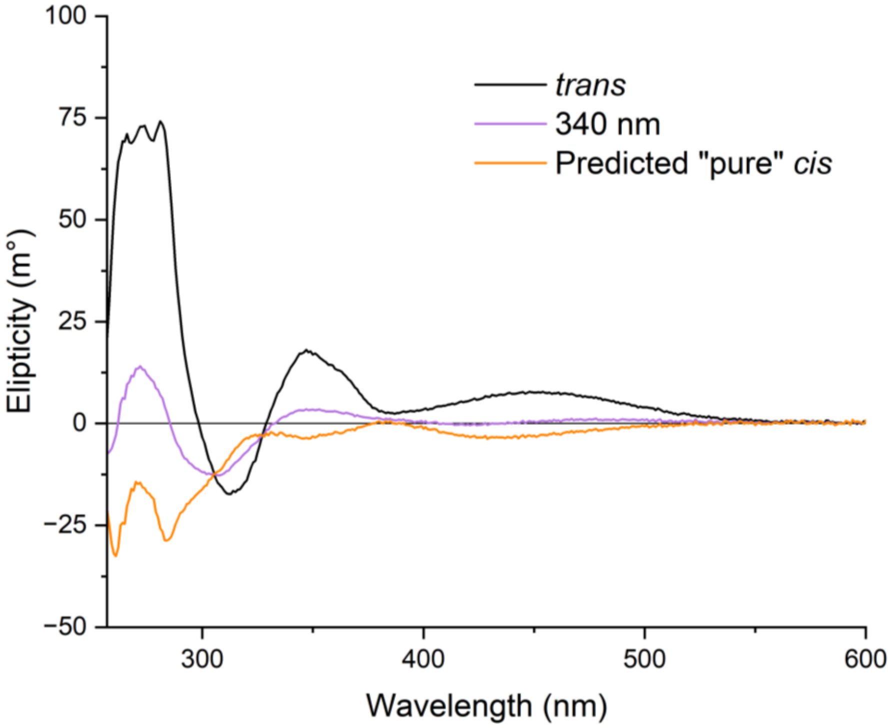

Supplementary Figure 24 CD measurements of **ACC-1** (50 µM, 1,2-dichloroethane, 25 °C) before and after 340 nm irradiation.

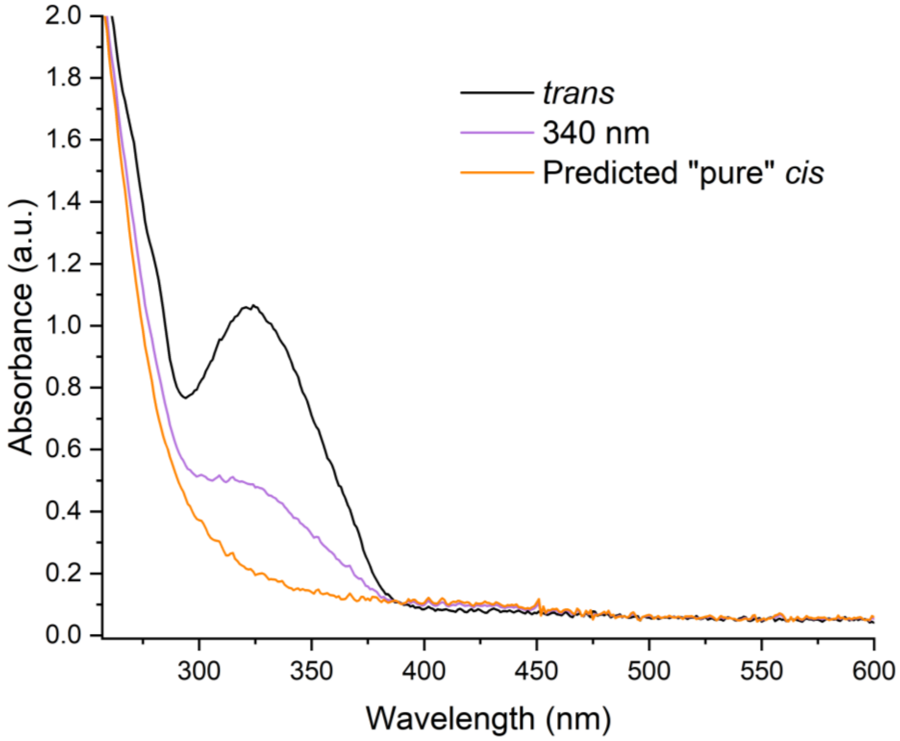

Supplementary Figure 25 Corresponding UV-Vis absorbance spectra from CD measurements (50 µM, 1,2-dichloroethane, 25 °C).

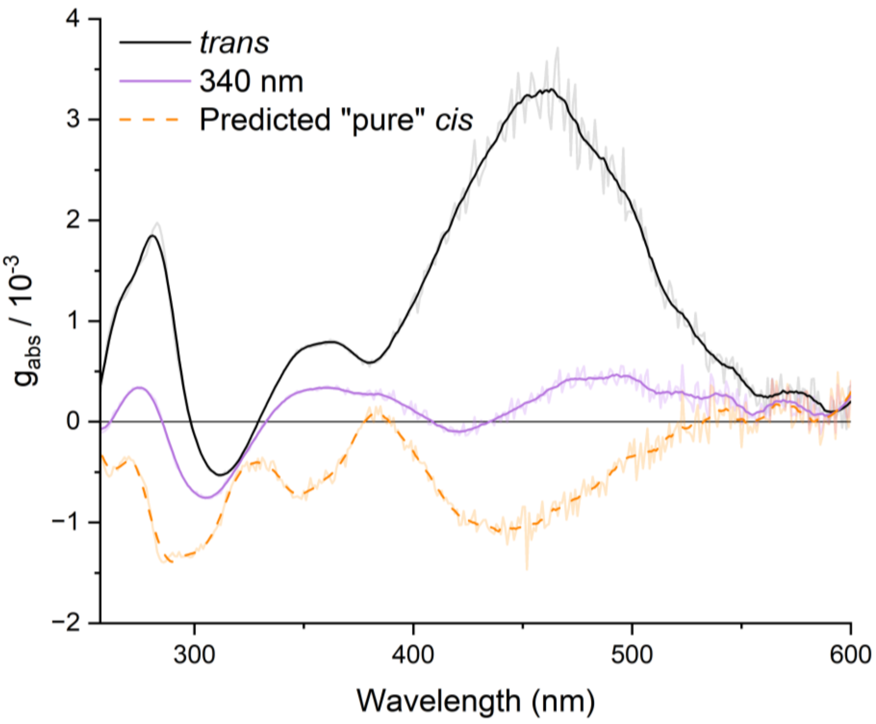

Supplementary Figure 26 Dissymmetry factor (g_{abs}) of **ACC-1** (50 µM, dry 1,2-dichloroethane, 25 °C).

6. Photophysical Properties of Azobenzene-Derived Organic Cages by NMR

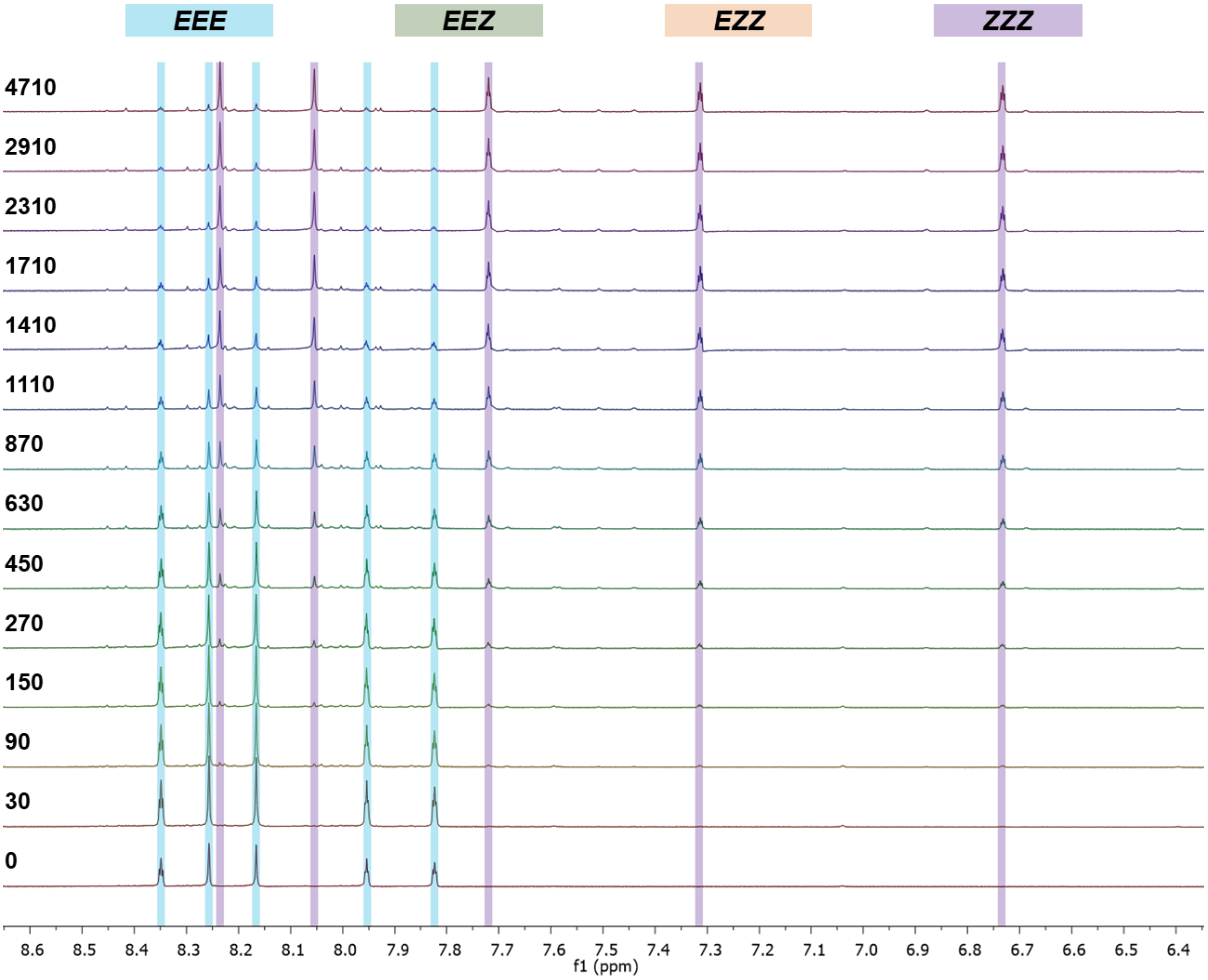

Supplementary Figure 27 Stacked ^1H NMR spectra in 1,2-dichloroethane-d_4 of ambient and irradiated (340 nm) samples of **ACC-1**. Time shown is the cumulative irradiation time (in seconds) with 340 nm light.

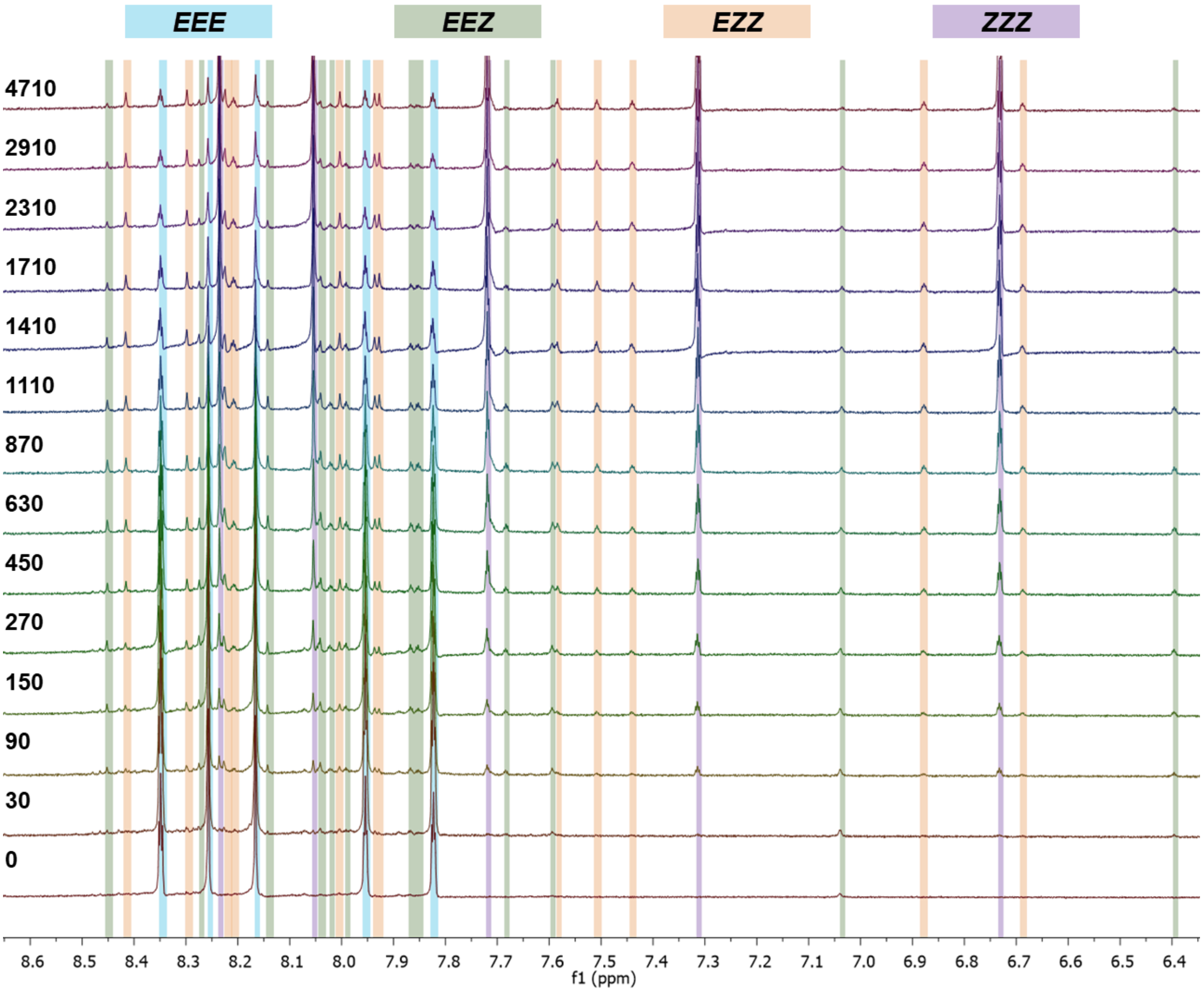

Supplementary Figure 28 Stacked ^{1}H NMR spectra in 1,2-dichloroethane-d_4 of ambient and irradiated samples of **ACC-1** with increased peak height. Time shown is the cumulative irradiation time (in seconds) with 340 nm light.

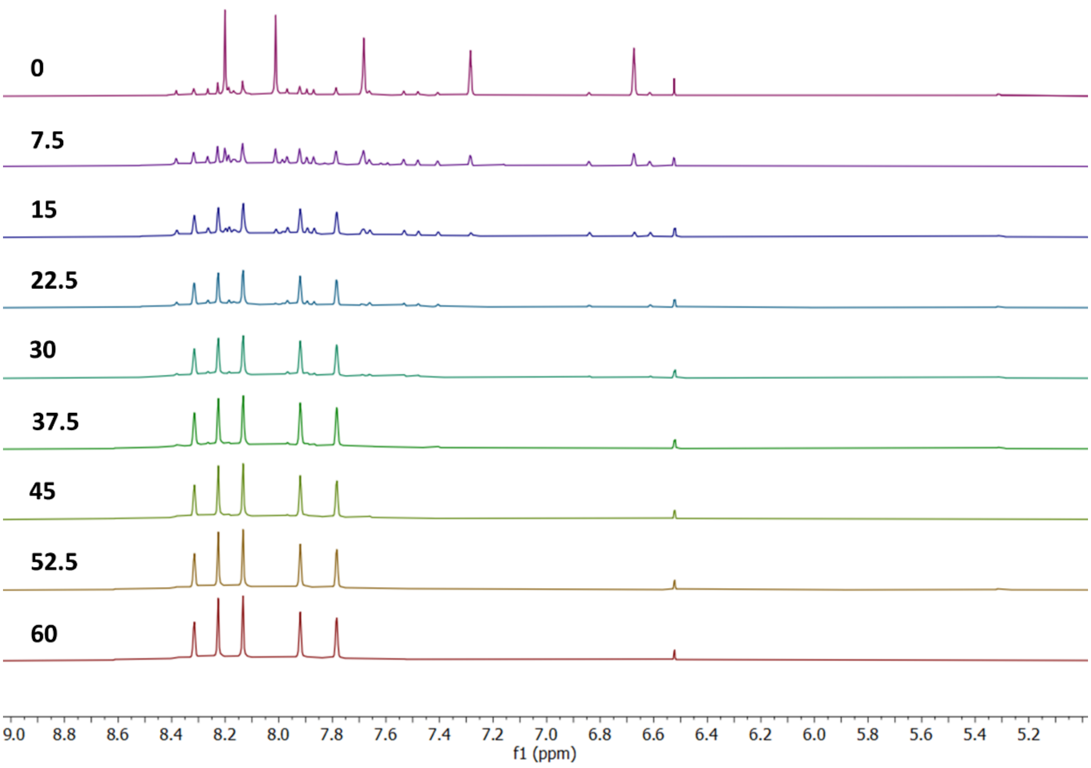

Supplementary Figure 29 Stacked ^{1}H NMR spectra in dry 1,2-dichloroethane of **ACC-1** after irradiation with 340 nm light. Time shown is the cumulative irradiation time (in hours) of the thermal isomerisation.

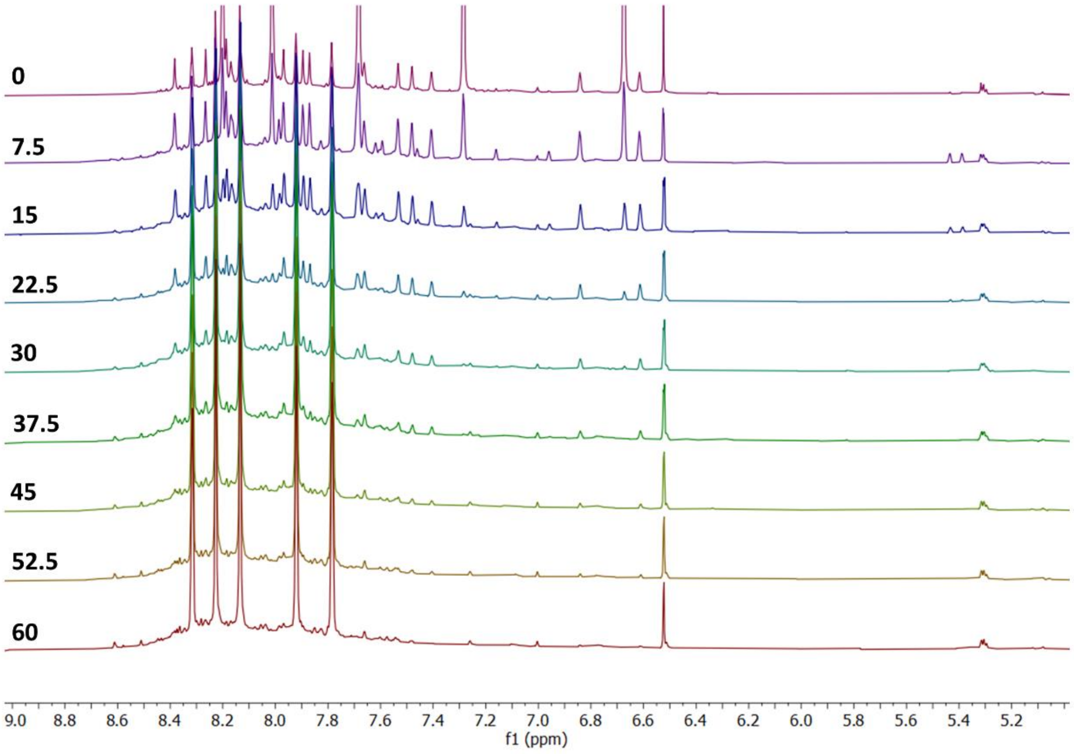

Supplementary Figure 30 Stacked ^{1}H NMR spectra with increased height in dry 1,2-dichloroethane of **ACC-1** after irradiation with 340 nm light. Time shown is the cumulative irradiation time (in hours) of the thermal isomerisation.

Kinetics of thermal isomerization

Kinetic equations can be derived from a series of stepwise isomerizations:

$$ZZZ \overset{k_1}{\rightarrow} EZZ \overset{k_2}{\rightarrow} EEZ \overset{k_3}{\rightarrow} EEE \qquad (eq. 1)$$

The concentration of the *ZZZ*-isomer, [*ZZZ*], follows from a first-order reaction rate:

$$[ZZZ] = [ZZZ]_0 \exp(-k_1 t) \qquad (eq. 2)$$

Equations for subsequent isomer concentrations are derived by substituting the equation for the concentration of the previous isomer in (1) and solving the resulting differential equation, leading to the following rate equations:

$$[EZZ] = \frac{k_1[ZZZ]_0}{k_2 - k_1}\exp(-k_1 t) + \left([EZZ]_0 - \frac{k_1[ZZZ]_0}{k_2 - k_1}\right)\exp(-k_2 t) \qquad (eq. 3)$$

$$\begin{aligned}[EEZ] = {} & \frac{k_1 k_2[ZZZ]_0}{(k_2 - k_1)(k_3 - k_1)}\exp(-k_1 t) + \frac{k_2}{k_3 - k_2}\left([EZZ]_0 - \frac{k_1[ZZZ]_0}{k_2 - k_1}\right)\exp(-k_2 t) \\ & + \left([EEZ]_0 - \frac{k_1 k_2[ZZZ]_0}{(k_2 - k_1)(k_3 - k_1)} - \frac{k_2}{k_3 - k_2}\left([EZZ]_0 - \frac{k_1[ZZZ]_0}{k_2 - k_1}\right)\right)\exp(-k_3 t) \qquad (eq. 4)\end{aligned}$$

$$\begin{aligned}[EEE] = {} & -\frac{k_2 k_3[ZZZ]_0}{(k_2 - k_1)(k_3 - k_1)}\exp(-k_1 t) - \frac{k_3}{k_3 - k_2}\left([EZZ]_0 - \frac{k_1[ZZZ]_0}{k_2 - k_1}\right)\exp(-k_2 t) \\ & - \left([EEZ]_0 - \frac{k_1 k_2[ZZZ]_0}{(k_2 - k_1)(k_3 - k_1)} - \frac{k_2}{k_3 - k_2}\left([EZZ]_0 - \frac{k_1[ZZZ]_0}{k_2 - k_1}\right)\right)\exp(-k_3 t) + [EEE]_0 \\ & + \frac{k_2 k_3[ZZZ]_0}{(k_2 - k_1)(k_3 - k_1)} + \frac{k_3}{k_3 - k_2}\left([EZZ]_0 - \frac{k_1[ZZZ]_0}{k_2 - k_1}\right) \\ & + \left([EEZ]_0 - \frac{k_1 k_2[ZZZ]_0}{(k_2 - k_1)(k_3 - k_1)} - \frac{k_2}{k_3 - k_2}\left([EZZ]_0 - \frac{k_1[ZZZ]_0}{k_2 - k_1}\right)\right) \qquad (eq. 5)\end{aligned}$$

The derivations for (eq. 3–5) are provided below.

Integrals from ^{1}H NMR spectroscopy were fitted according to these kinetic equations sequentially, using the resulting kinetic rate constant from the prior equations to avoid overfitting.

Derivation of (eq. 3)

From (eq. 1), the rate of change of [*EZZ*] is given by:

$$\frac{d[EZZ]}{dt} = k_1[ZZZ] - k_2[EZZ]$$

Substituting the rate equation for [*ZZZ*] (eq. 2), we obtain:

$$\frac{d[EZZ]}{dt} + k_2[EZZ] = k_1[ZZZ]_0 \exp\,(-k_1 t)$$

By use of the integrating factor $\exp(k_2 t)$, and noting that:

$$\frac{d}{dt}(\exp(k_2 t)\,[EZZ]) = \exp(k_2 t)\left(\frac{d[EZZ]}{dt} + k_2[EZZ]\right)$$

the differential equation becomes separable:

$$\frac{d}{dt}(\exp(k_2 t)\,[EZZ]) = k_1[ZZZ]_0 \exp\big((k_2 - k_1)t\big)$$

Solving this, we obtain:

$$[EZZ] = \frac{k_1[ZZZ]_0}{k_2 - k_1}\exp(-k_1 t) + A\exp(-k_2 t)$$

Setting $[EZZ] = [EZZ]_0$ at time $t = 0$,

$$A = [EZZ]_0 - \frac{k_1[ZZZ]_0}{k_2 - k_1}$$

Hence the rate equation for [*EZZ*] is:

$$[EZZ] = \frac{k_1[ZZZ]_0}{k_2 - k_1}\exp(-k_1 t) + \left([EZZ]_0 - \frac{k_1[ZZZ]_0}{k_2 - k_1}\right)\exp(-k_2 t) \qquad (eq.3)$$

Derivation of (eq. 4)

A similar derivation is required for (eq. 4):

$$\frac{d[EEZ]}{dt} = k_2[EZZ] - k_3[EEZ]$$

Substituting (eq. 3):

$$\frac{d[EEZ]}{dt} + k_3[EEZ] = \frac{k_1k_2[ZZZ]_0}{k_2 - k_1}\exp(-k_1t) + k_2\left([EZZ]_0 - \frac{k_1[ZZZ]_0}{k_2 - k_1}\right)\exp(-k_2t)$$

Use of the integrating factor $\exp(k_3t)$, noting that:

$$\frac{d}{dt}(\exp(k_3t)\,[EEZ]) = \exp(k_3t)\left(\frac{d[EEZ]}{dt} + k_3[EEZ]\right)$$

yields a separable differential equation:

$$\frac{d}{dt}(\exp(k_3t)\,[EEZ]) =$$

$$= \frac{k_1k_2[ZZZ]_0}{k_2 - k_1}\exp((k_3 - k_1)t) + k_2\left([EZZ]_0 - \frac{k_1[ZZZ]_0}{k_2 - k_1}\right)\exp((k_3 - k_2)t)$$

The solution to which is:

$$[EEZ] = \frac{k_1k_2[ZZZ]_0}{(k_2 - k_1)(k_3 - k_1)}\exp(-k_1t) + \frac{k_2}{k_3 - k_2}\left([EZZ]_0 - \frac{k_1[ZZZ]_0}{k_2 - k_1}\right)\exp(-k_2t)$$
$$+ B\exp(-k_3t)$$

And finally, setting $[EEZ] = [EEZ]_0$ at time $t = 0$,

$$B = [EEZ]_0 - \frac{k_1k_2[ZZZ]_0}{(k_2 - k_1)(k_3 - k_1)} - \frac{k_2}{k_3 - k_2}\left([EZZ]_0 - \frac{k_1[ZZZ]_0}{k_2 - k_1}\right)$$

Hence the rate equation for *[EEZ]* is:

$$[EEZ] = \frac{k_1k_2[ZZZ]_0}{(k_2 - k_1)(k_3 - k_1)}\exp(-k_1t) + \frac{k_2}{k_3 - k_2}\left([EZZ]_0 - \frac{k_1[ZZZ]_0}{k_2 - k_1}\right)\exp(-k_2t)$$
$$+\left([EEZ]_0 - \frac{k_1k_2[ZZZ]_0}{(k_2 - k_1)(k_3 - k_1)} - \frac{k_2}{k_3 - k_2}\left([EZZ]_0 - \frac{k_1[ZZZ]_0}{k_2 - k_1}\right)\right)\exp(-k_3t) \qquad (eq.4)$$

Derivation of (eq. 5)

The derivation of (eq. 5) only requires solving the differential equation directly:

$$\frac{d[EEE]}{dt} = k_3[EEZ]$$

This results in:

$$[EEE] = -\frac{k_2k_3[ZZZ]_0}{(k_2-k_1)(k_3-k_1)}\exp(-k_1t) - \frac{k_3}{k_3-k_2}\left([EZZ]_0 - \frac{k_1[ZZZ]_0}{k_2-k_1}\right)\exp(-k_2t)$$
$$-\left([EEZ]_0 - \frac{k_1k_2[ZZZ]_0}{(k_2-k_1)(k_3-k_1)} - \frac{k_2}{k_3-k_2}\left([EZZ]_0 - \frac{k_1[ZZZ]_0}{k_2-k_1}\right)\right)\exp(-k_3t) + C$$

Setting $[EEE] = [EEE]_0$ at time $t = 0$,

$$C = [EEE]_0 + \frac{k_2k_3[ZZZ]_0}{(k_2-k_1)(k_3-k_1)} + \frac{k_3}{k_3-k_2}\left([EZZ]_0 - \frac{k_1[ZZZ]_0}{k_2-k_1}\right)$$
$$+\left([EEZ]_0 - \frac{k_1k_2[ZZZ]_0}{(k_2-k_1)(k_3-k_1)} - \frac{k_2}{k_3-k_2}\left([EZZ]_0 - \frac{k_1[ZZZ]_0}{k_2-k_1}\right)\right)$$

Hence the rate equation for [*EEE]* is:

$$[EEE] = -\frac{k_2k_3[ZZZ]_0}{(k_2-k_1)(k_3-k_1)}\exp(-k_1t) - \frac{k_3}{k_3-k_2}\left([EZZ]_0 - \frac{k_1[ZZZ]_0}{k_2-k_1}\right)\exp(-k_2t)$$
$$-\left([EEZ]_0 - \frac{k_1k_2[ZZZ]_0}{(k_2-k_1)(k_3-k_1)} - \frac{k_2}{k_3-k_2}\left([EZZ]_0 - \frac{k_1[ZZZ]_0}{k_2-k_1}\right)\right)\exp(-k_3t) + [EEE]_0$$
$$+\frac{k_2k_3[ZZZ]_0}{(k_2-k_1)(k_3-k_1)} + \frac{k_3}{k_3-k_2}\left([EZZ]_0 - \frac{k_1[ZZZ]_0}{k_2-k_1}\right)$$
$$+\left([EEZ]_0 - \frac{k_1k_2[ZZZ]_0}{(k_2-k_1)(k_3-k_1)} - \frac{k_2}{k_3-k_2}\left([EZZ]_0 - \frac{k_1[ZZZ]_0}{k_2-k_1}\right)\right) \qquad (eq.5)$$

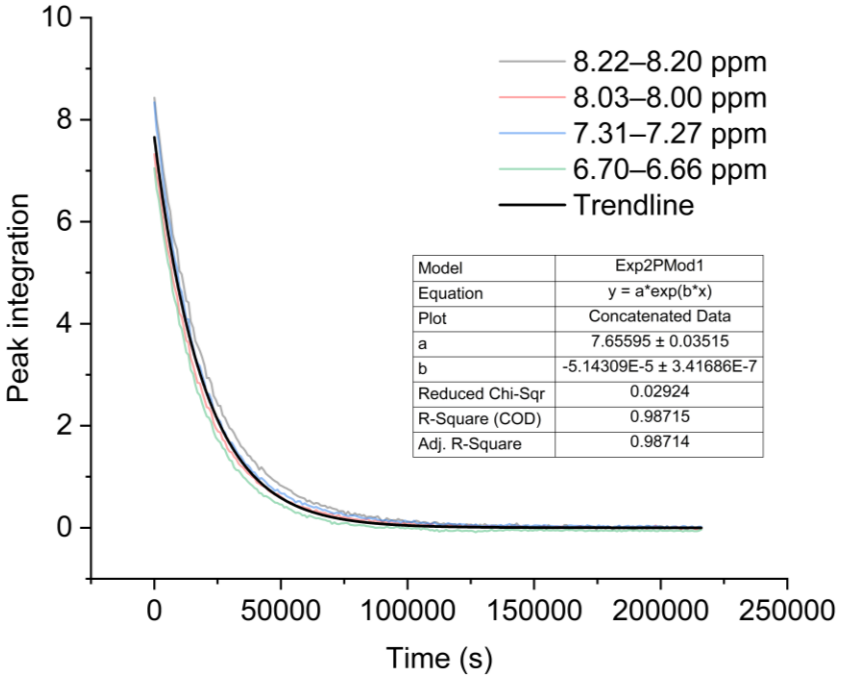

Supplementary Figure 31 ^{1}H NMR integrations of *ZZZ*-**ACC-1**, fitted according to equation (2). A rate constant of k_1 = -5.14 ± 0.03 × 10^{-5} s^{-1} was determined, with an associated thermal half-life of 3.74 ± 0.02 hours.

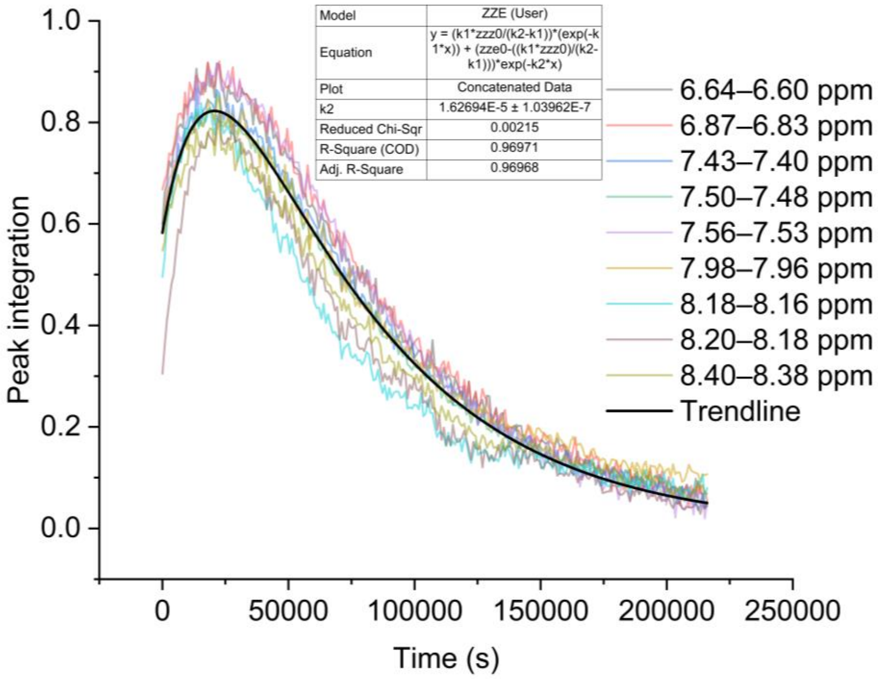

Supplementary Figure 32 ^{1}H NMR integrations of *EZZ*-**ACC-1**, fitted according to equation (3). A rate constant of k_2 = -1.63 ± 0.01 × 10^{-5} s^{-1} was determined, with an associated thermal half-life of 11.83 ± 0.08 hours.

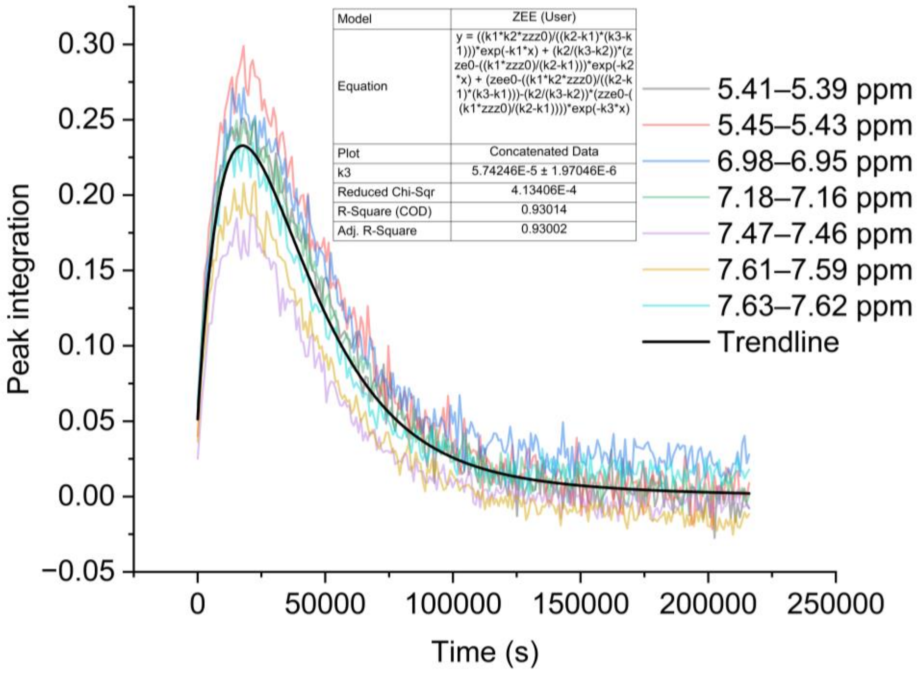

Supplementary Figure 33 ^{1}H NMR integrations of *EEZ*-**ACC-1**, fitted according to equation (4). A rate constant of k_3 = -5.7 ± 0.2 × 10^{-5} s^{-1} was determined, with an associated thermal half-life of 3.4 ± 0.1 hours.

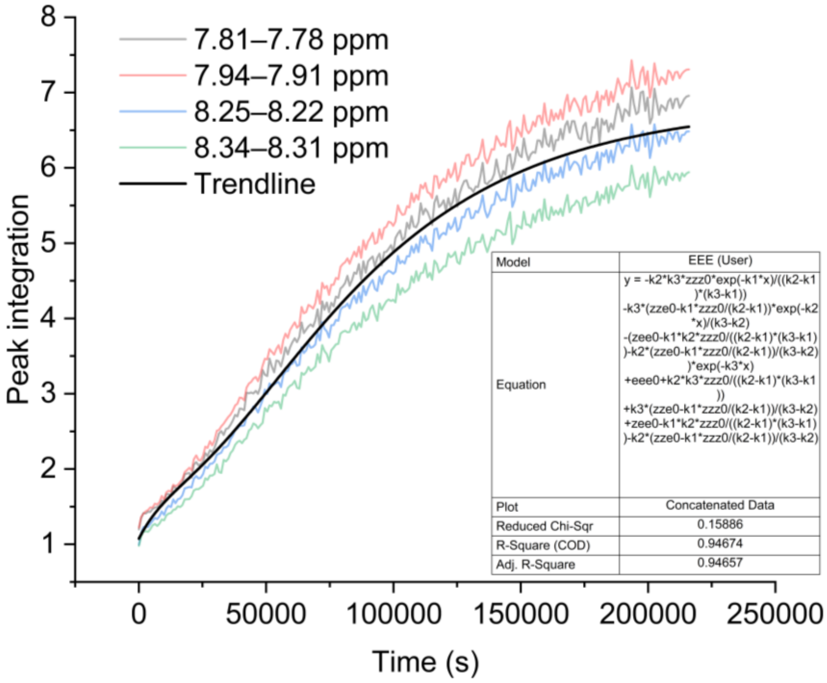

Supplementary Figure 34 ^{1}H NMR integrations of *EEE*-**ACC-1**, fitted according to equation (5) using the rate constants derived above.

7. Diffusion-Ordered ^{1}H NMR Spectroscopy (DOSY)

A ^{1}H DOSY experiment was conducted on a sample of **ACC-1** in anhydrous 1,2-dichloroethane after irradiation with 340 nm light in a quartz NMR tube (Figure S35–S36).

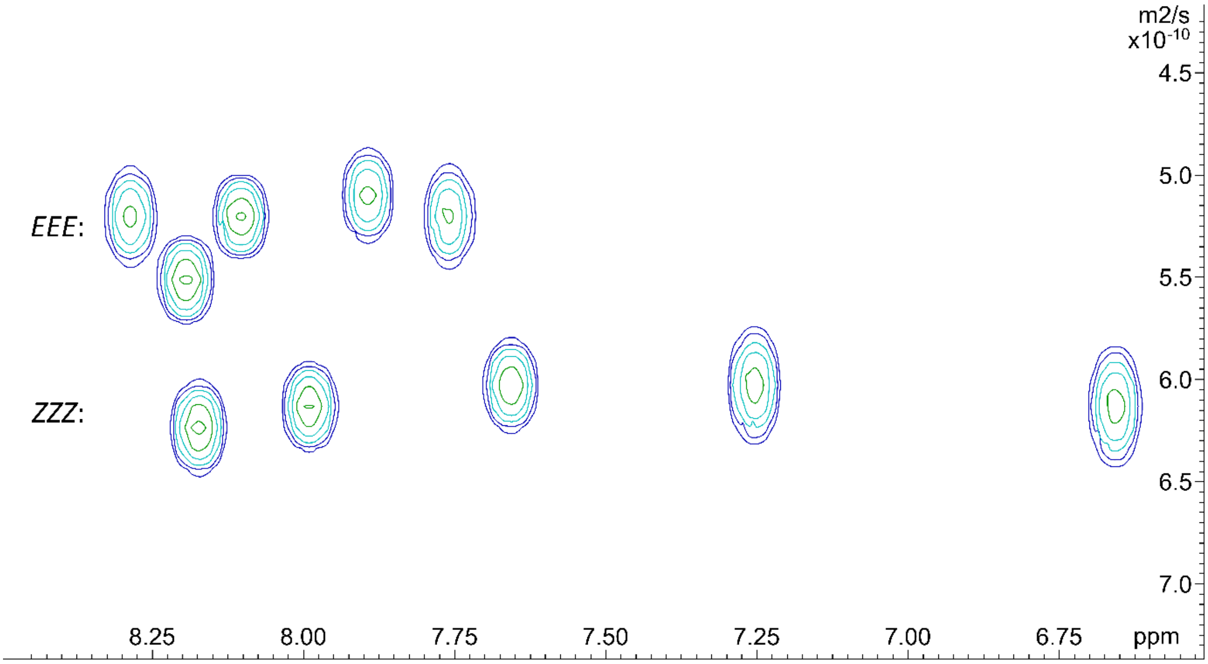

Supplementary Figure 35 ^{1}H DOSY spectrum of **ACC-1** after irradiation with 340 nm.

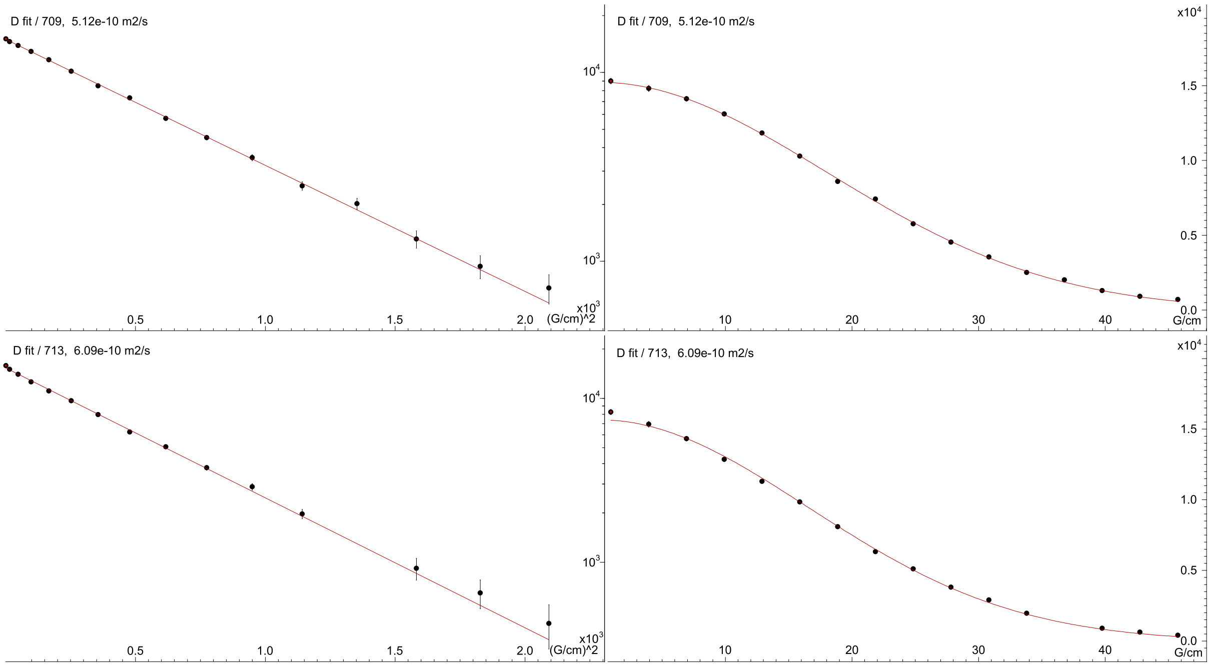

Supplementary Figure 36 Representative fits of ^{1}H DOSY data for *EEE*-**ACC-1** (top) and *ZZZ*-**ACC-1** (bottom).

Supplementary Table 5 Summary of ^1H DOSY data of **ACC-1** after 340 nm irradiation.

ACC-1 isomer	Chemical shift / ppm	Diffusion coefficient, D / $m^2 s^{-1}$	Error / $m^2 s^{-1}$
EEE	8.28	5.15×10^{-10}	0.12×10^{-10}
EEE	8.19	5.52×10^{-10}	0.10×10^{-10}
ZZZ	8.17	6.23×10^{-10}	0.10×10^{-10}
EEE	8.10	5.17×10^{-10}	0.09×10^{-10}
ZZZ	7.99	6.15×10^{-10}	0.10×10^{-10}
EEE	7.89	5.12×10^{-10}	0.16×10^{-10}
EEE	7.76	5.19×10^{-10}	0.13×10^{-10}
ZZZ	7.65	6.05×10^{-10}	0.11×10^{-10}
ZZZ	7.25	6.00×10^{-10}	0.13×10^{-10}
ZZZ	6.66	6.09×10^{-10}	0.14×10^{-10}

Viscosity measurements were conducted on the NMR sample, with an average value of $\eta = 0.861 \pm 0.003$ mPa s (room temperature, 294.0 K). The viscosity was corrected to the same temperature as the ^1H DOSY experiment (297 K) based on a linear regression (gradient = -0.00931 mPa s K^{-1}) between literature data of 1,2-dichloroethane viscosities at 293.15 K (0.8385 mPa s) and 313.15 K (0.6523 mPa s),[27] giving a final value of $\eta = 0.833 \pm 0.003$ mPa s. Solvodynamic radii were calculated based on the Stokes–Einstein–Sutherland equation (assuming spherical particle diffusion): $R_S = (k_B T)/6\pi\eta D$ (Table S6).

Supplementary Table 6 Average diffusion coefficients of ^1H DOSY peaks of *EEE*- and *ZZZ*-**ACC-1**, and corresponding solvodynamic radii, showing a difference in size (p = 0.0001).

ACC-1 isomer	Diffusion coefficient, D / $m^2 s^{-1}$	Solvodynamic radius, R_S / Å
EEE	$(5.23 \pm 0.16) \times 10^{-10}$	4.99 ± 0.15
ZZZ	$(6.10 \pm 0.09) \times 10^{-10}$	4.28 ± 0.06

A ^{1}H DOSY experiment was conducted on a sample of **ACC-2** in anhydrous dichloromethane-d_2 after irradiation with 365 nm light (Figure S37–S38).

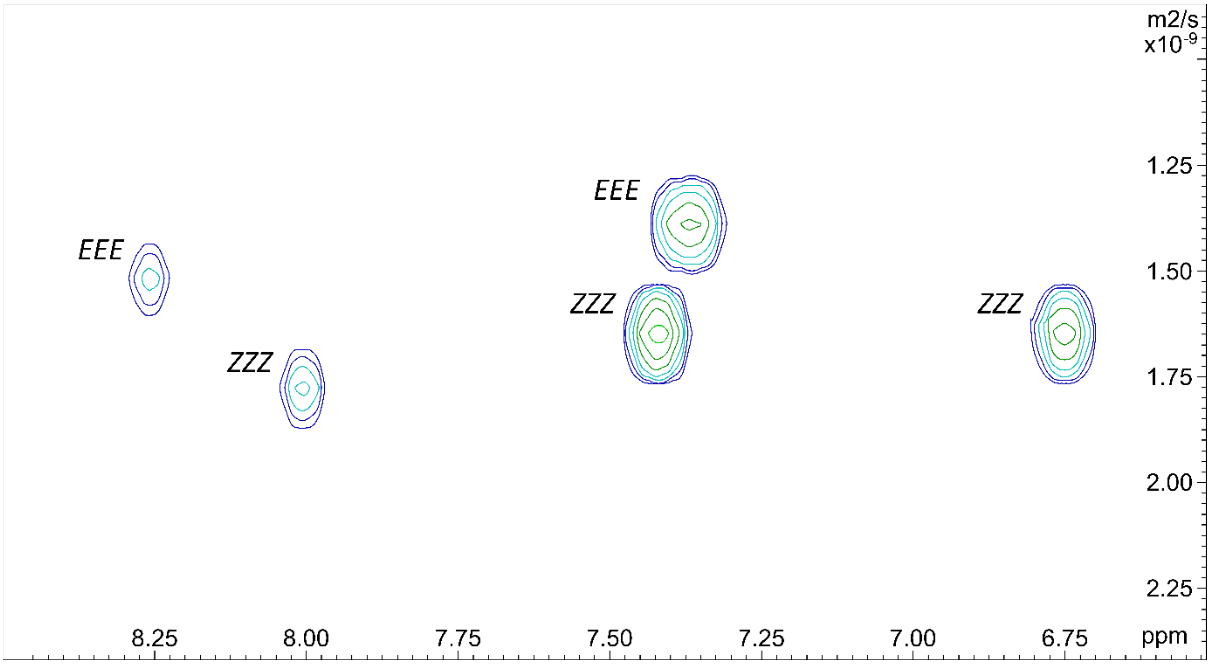

Supplementary Figure 37 ^{1}H DOSY spectrum of **ACC-2** after irradiation with 365 nm.

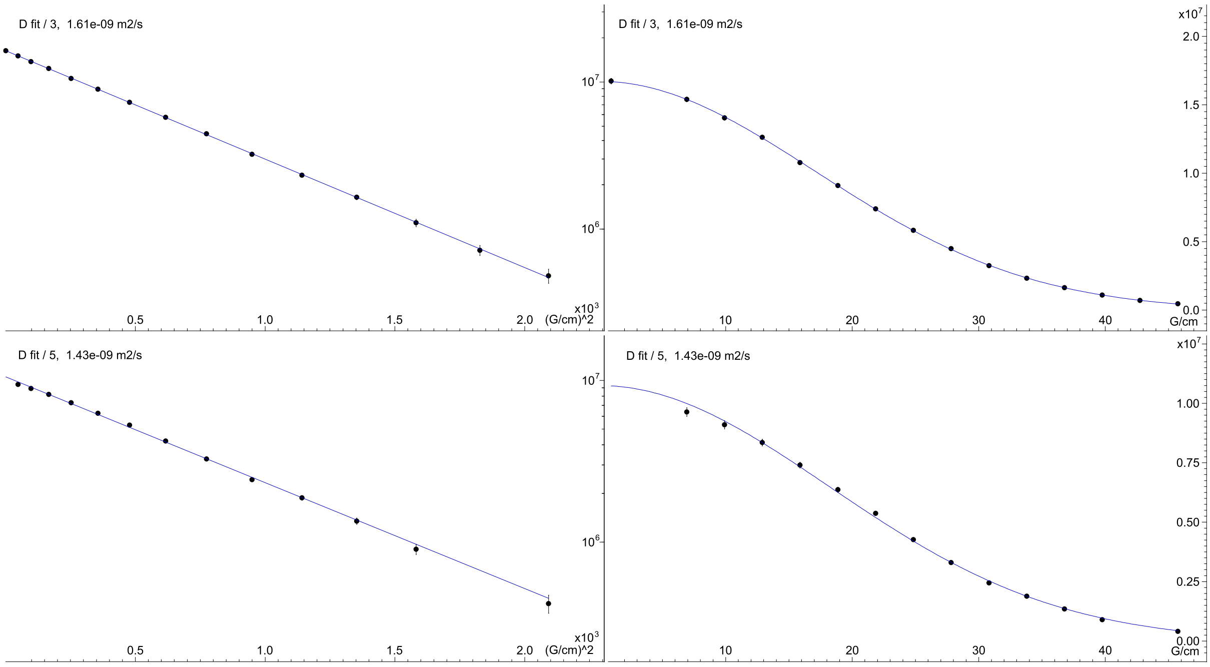

Supplementary Figure 38 Representative fits of ^{1}H DOSY data for *EEE*-**ACC-2** (top) and *ZZZ*-**ACC-2** (bottom).

Supplementary Table 7 Summary of ^{1}H DOSY data of **ACC-2** after 365 nm irradiation.

**ACC-2 isomer**	**Chemical shift / ppm**	**Diffusion coefficient, D / $m^2\ s^{-1}$**	**Error / $m^2\ s^{-1}$**
EEE	8.25	1.46×10^{-9}	0.07×10^{-9}
ZZZ	8.00	1.76×10^{-9}	0.05×10^{-9}
ZZZ	7.43	1.61×10^{-9}	0.02×10^{-9}
EEE + ZZZ *Unresolved in ^{1}H*	7.41	1.61×10^{-9}	0.02×10^{-9}
EEE	7.39	1.43×10^{-9}	0.03×10^{-9}
EEE	7.36	1.40×10^{-9}	0.04×10^{-9}
EEE	7.34	1.41×10^{-9}	0.06×10^{-9}
ZZZ	6.76	1.64×10^{-9}	0.03×10^{-9}
ZZZ	6.74	1.67×10^{-9}	0.03×10^{-9}

Viscosity measurements were conducted on the NMR sample, with an average value of $\eta = 0.449 \pm 0.002$ mPa s (room temperature, 295.1 K). The viscosity was corrected to the same temperature as the ^{1}H DOSY experiment (297 K) based on a linear regression (gradient = -0.0048 mPa s K^{-1}) between literature data of dichloromethane viscosities at 293.15 K (0.437 mPa s)[28] and 298.15 K (0.413 mPa s),[29] giving a final value of $\eta = 0.444 \pm 0.002$ mPa s.

Supplementary Table 8 Average diffusion coefficients of ^{1}H DOSY peaks of *EEE*- and *ZZZ*-**ACC-2**, and corresponding solvodynamic radii, showing a difference in size ($p = 0.0007$).

**ACC-2** isomer	Diffusion coefficient, D / $m^2\ s^{-1}$	Solvodynamic radius, R_S / Å
EEE	$(1.43 \pm 0.02) \times 10^{-9}$	3.44 ± 0.06
ZZZ	$(1.67 \pm 0.06) \times 10^{-9}$	2.94 ± 0.11

8. High-Resolution Mass Spectra

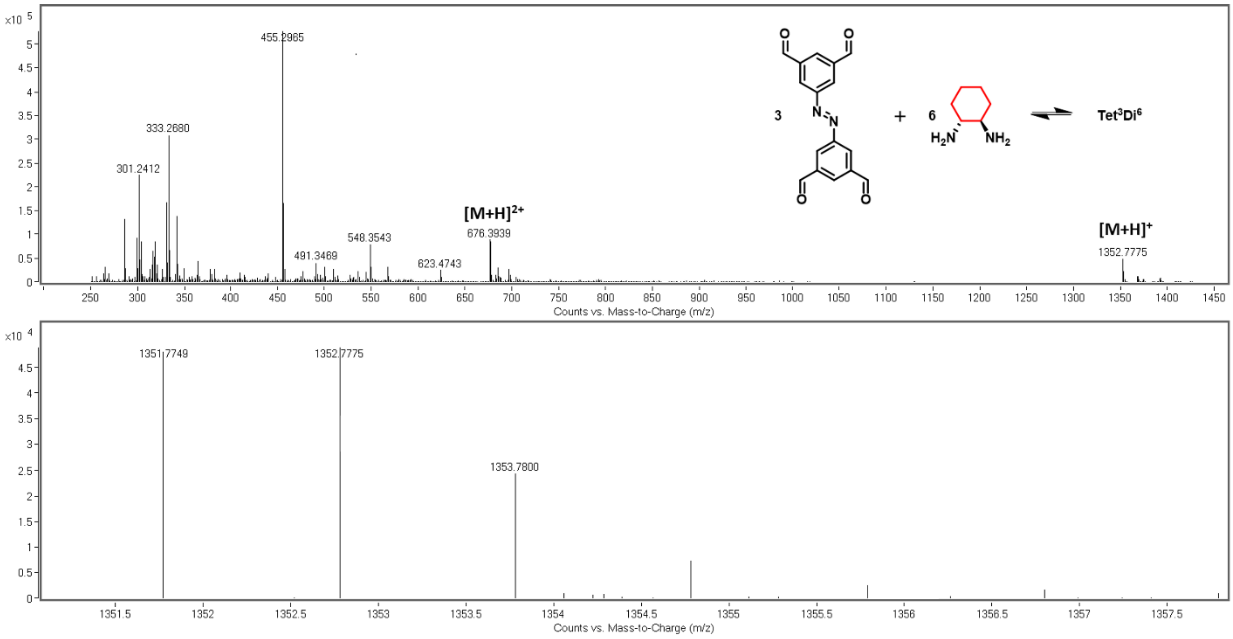

Supplementary Figure 39 HRMS spectra for the reaction of (*E*)-5,5'-(diazene-1,2-diyl)diisophthalaldehyde **4** with (1*R*,2*R*)-cyclohexanediamine after stirring at RT for 3 days. Indicating clean formation of the **Tet^{3}Di^6** cage $C_{84}H_{90}N_{18}$

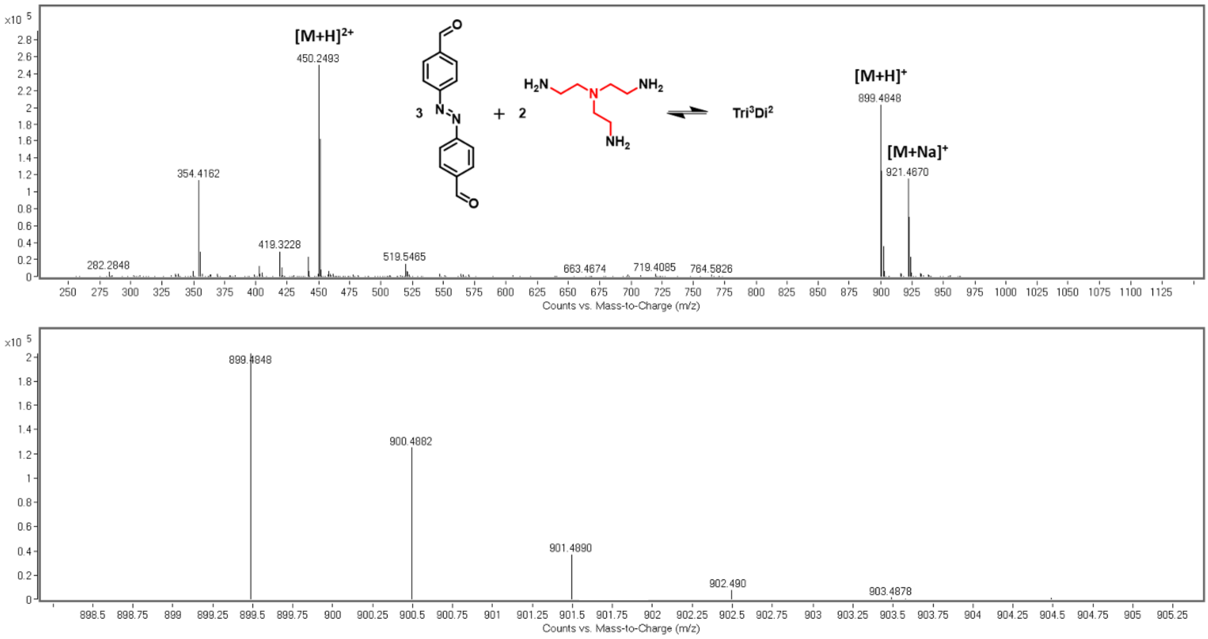

Supplementary Figure 40 HRMS spectra for the reaction of (*E*)-4,4'-(Diazene-1,2-diyl)dibenzaldehyde **8** with tris(2-aminoethyl)amine after stirring at RT for 3 days. Indicating clean formation of the **Tri^{2}Di^3** cage $C_{54}H_{54}N_{14}$

9. ^{1}H NMR and ^{13}C NMR Spectra

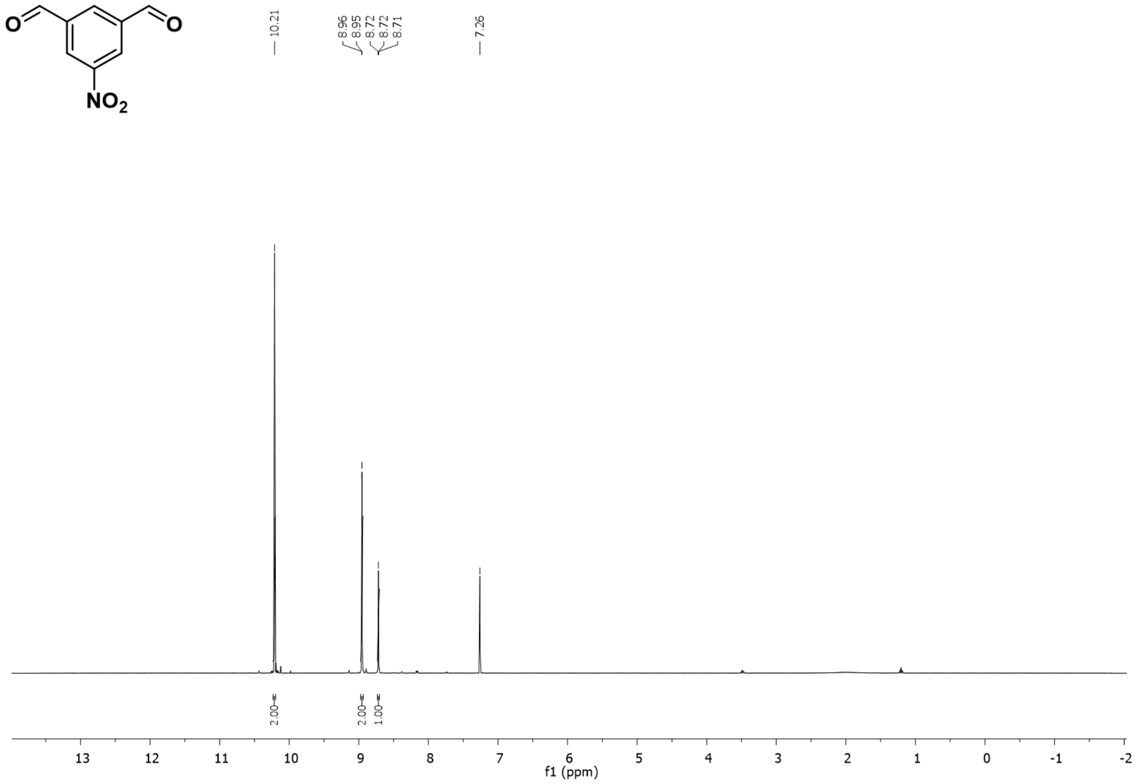

Supplementary Figure 41 ^{1}H NMR of **1** in $CDCl_3$

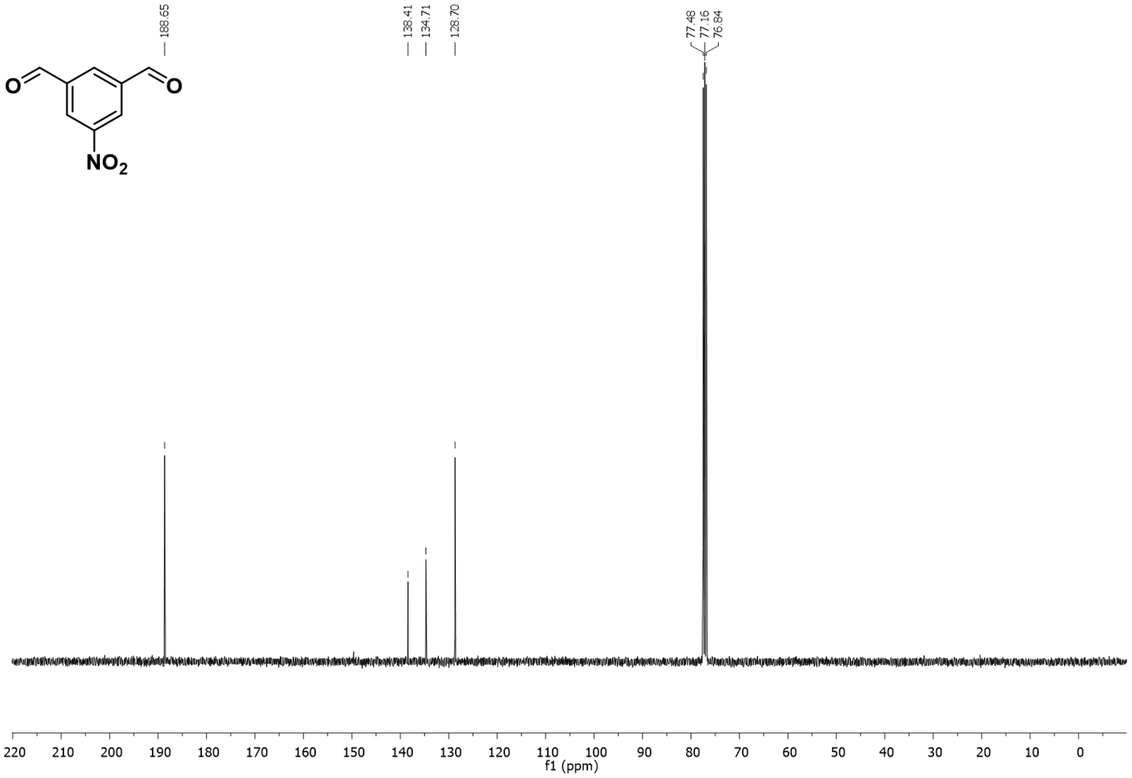

Supplementary Figure 42 ^{13}C NMR of **1** in $CDCl_3$

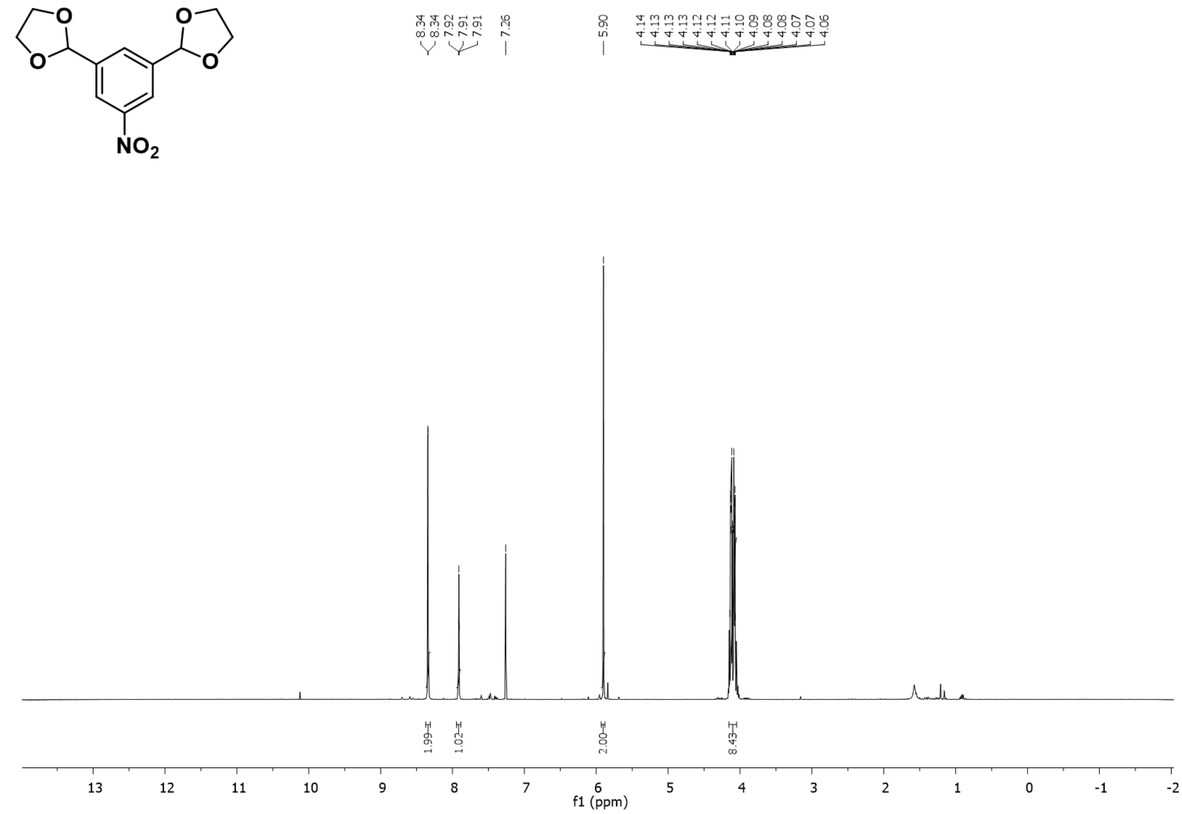

Supplementary Figure 43 ^{1}H NMR of **2** in $CDCl_3$

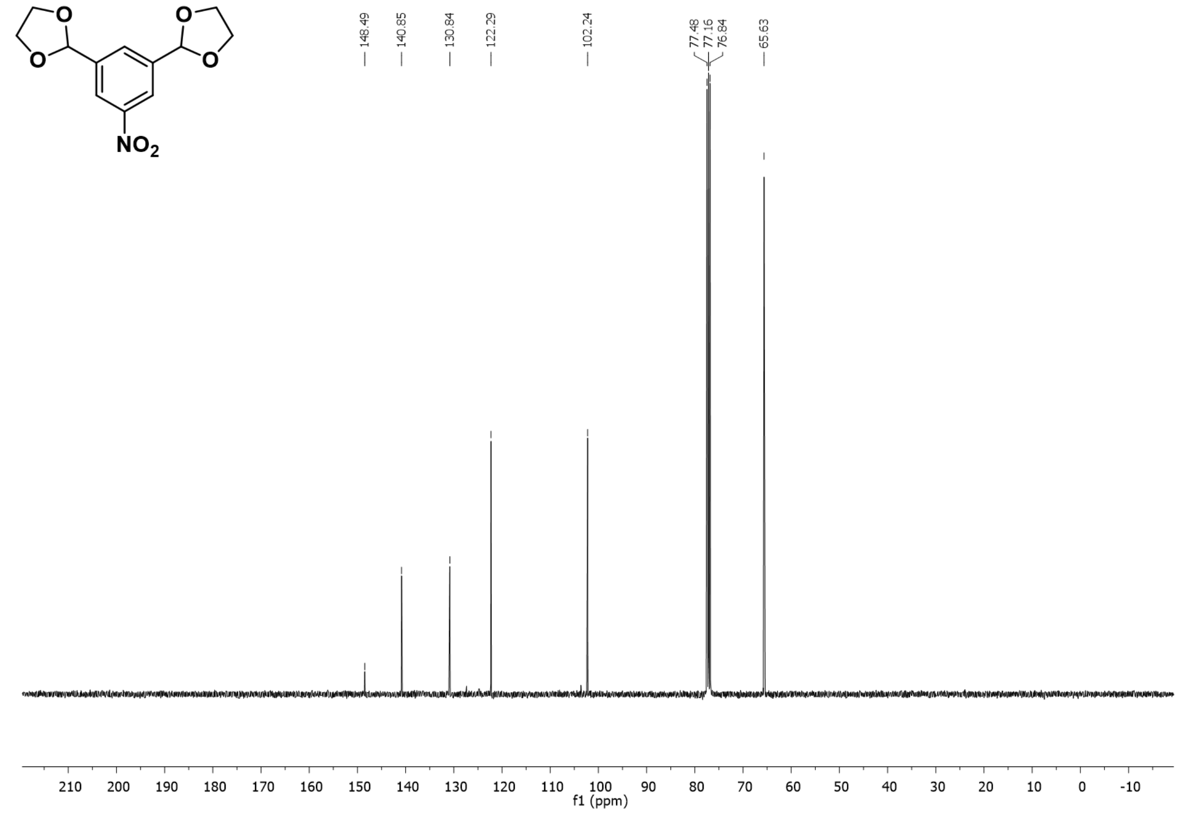

Supplementary Figure 44 ^{13}C NMR of **2** in $CDCl_3$

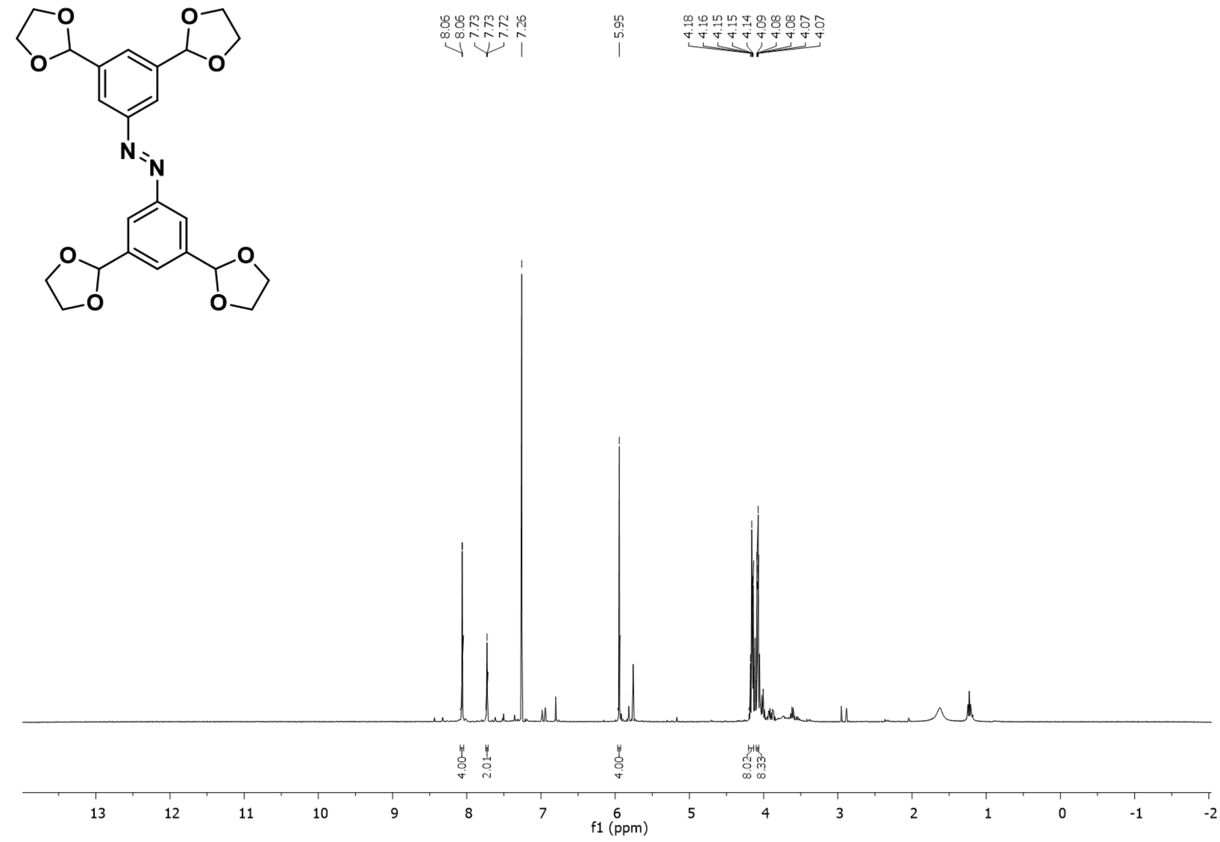

Supplementary Figure 45 ^{1}H NMR of **3** in $CDCl_3$

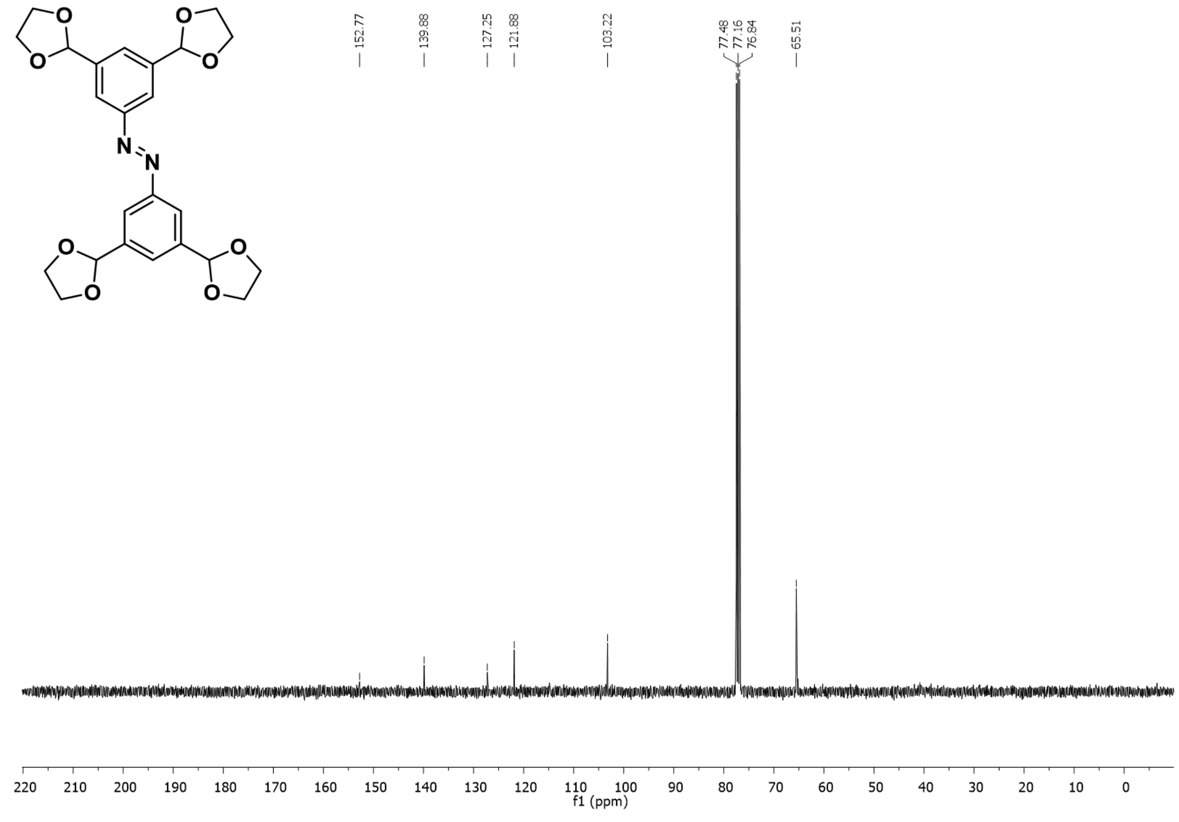

Supplementary Figure 46 ^{13}C NMR of **3** in $CDCl_3$

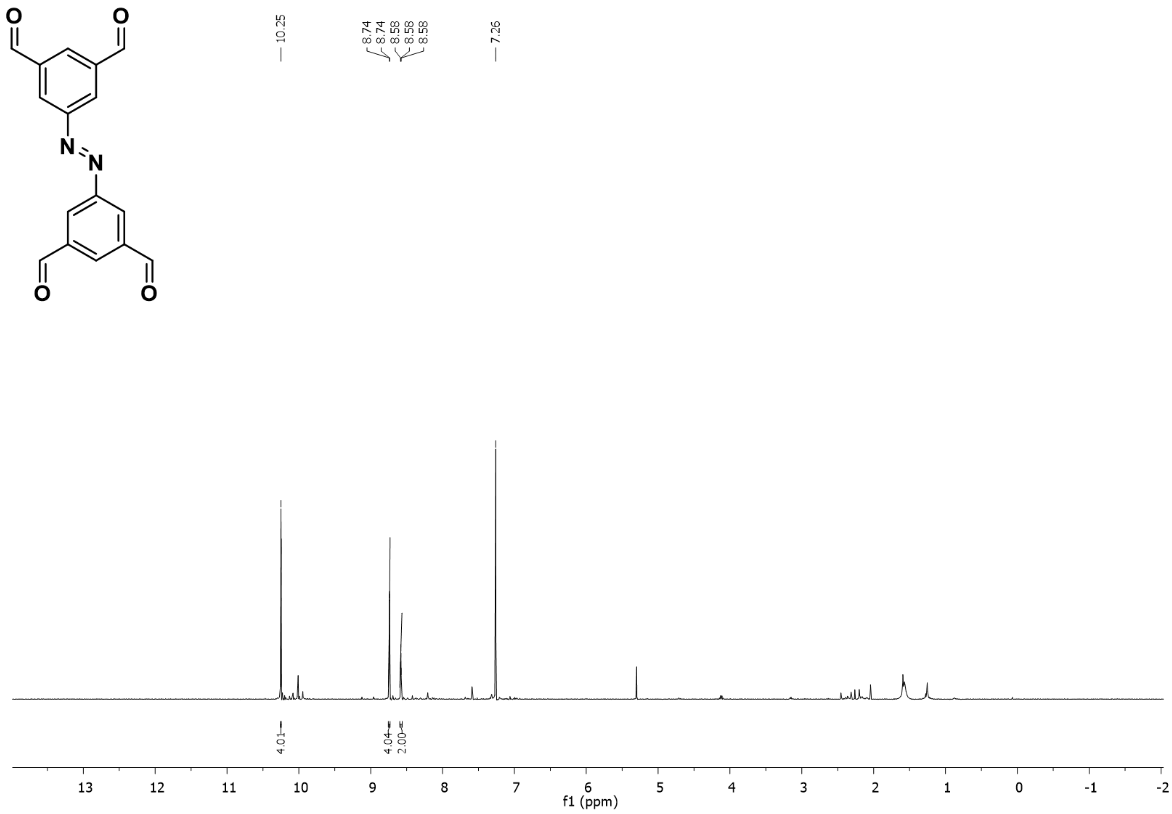

Supplementary Figure 47 ^{1}H NMR of **4** in $CDCl_3$

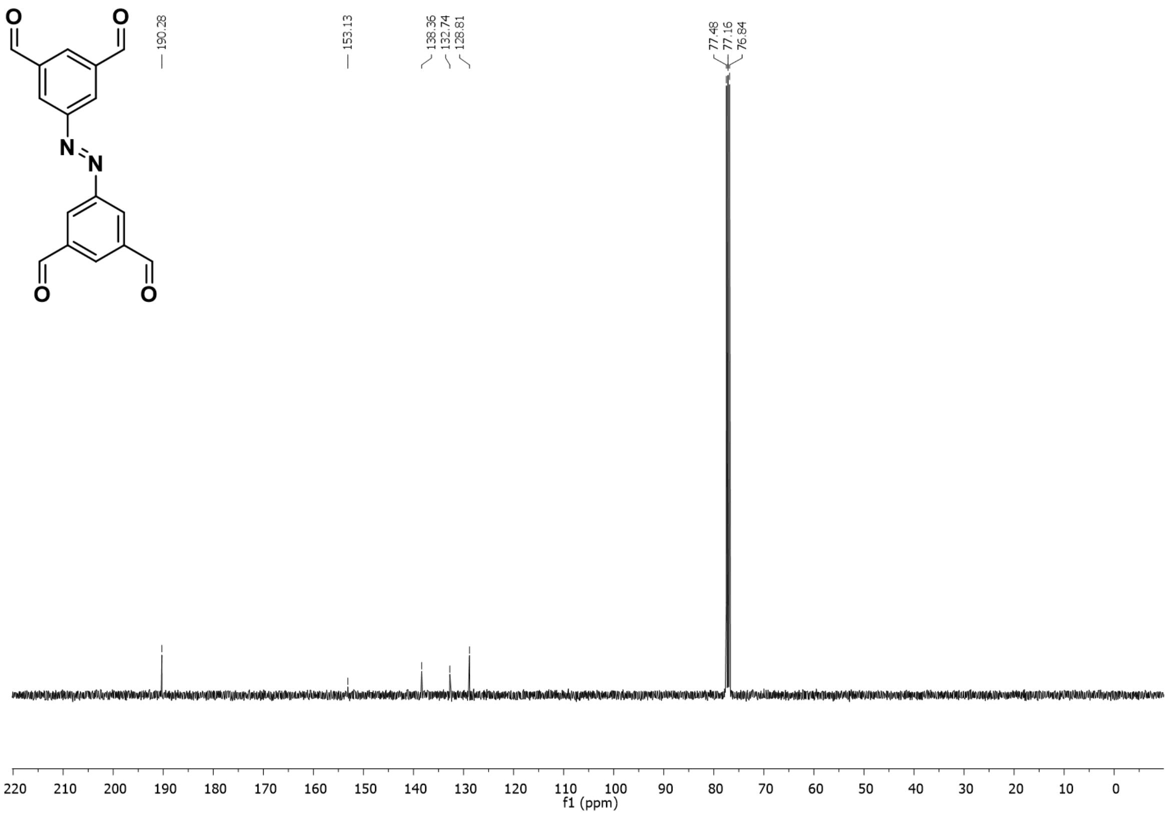

Supplementary Figure 48 ^{13}C NMR of **4** in $CDCl_3$

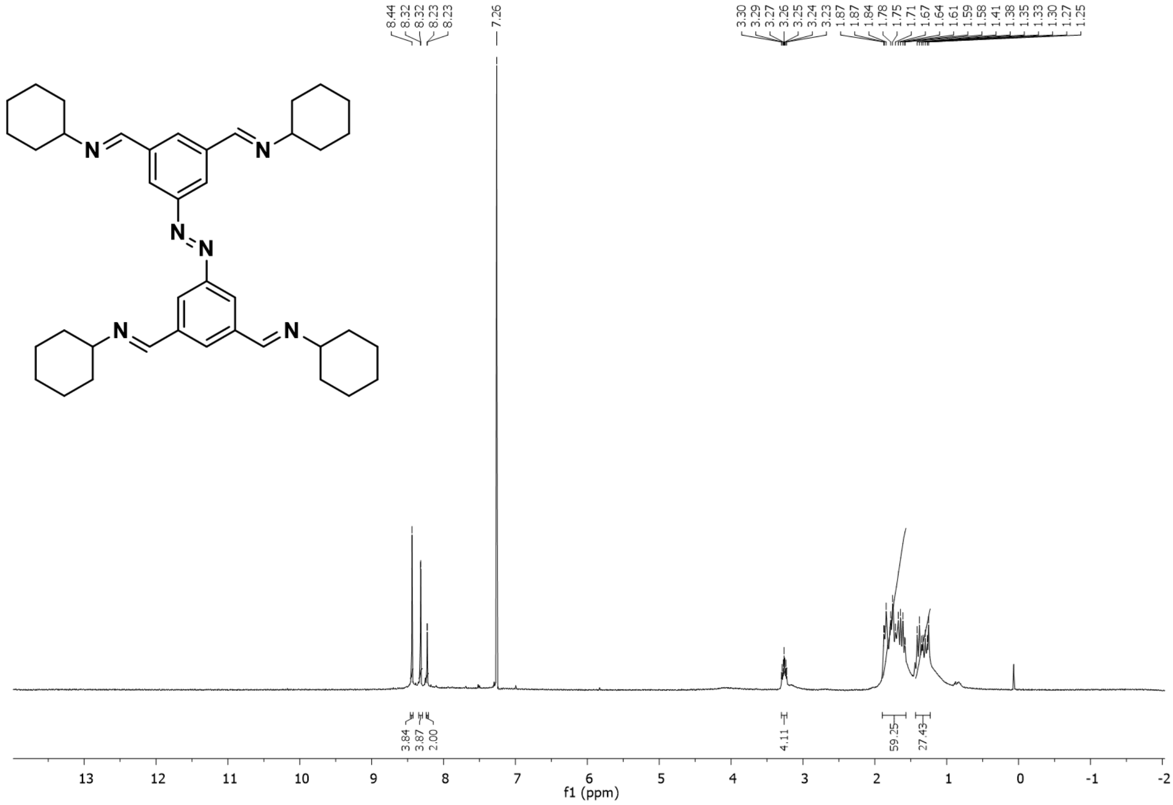

Supplementary Figure 49 ^{13}C NMR of **A1** in $CDCl_3$

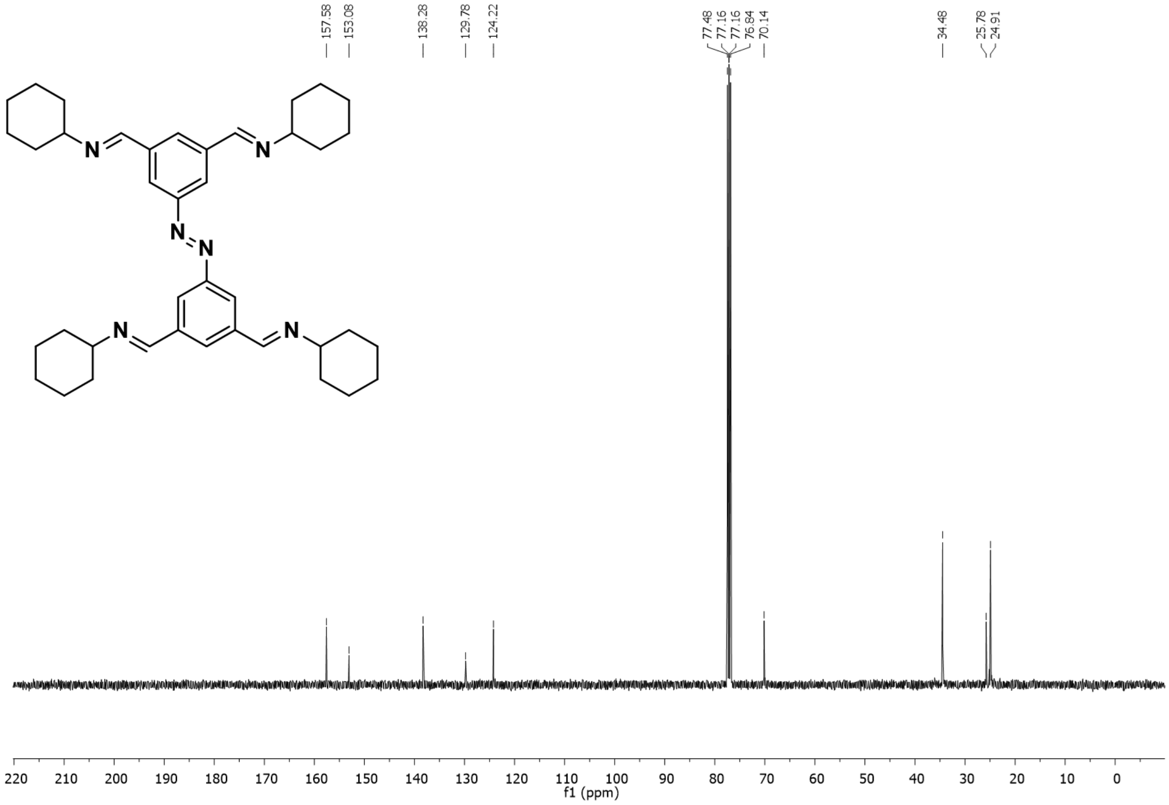

Supplementary Figure 50 ^{13}C NMR of **A1** in $CDCl_3$

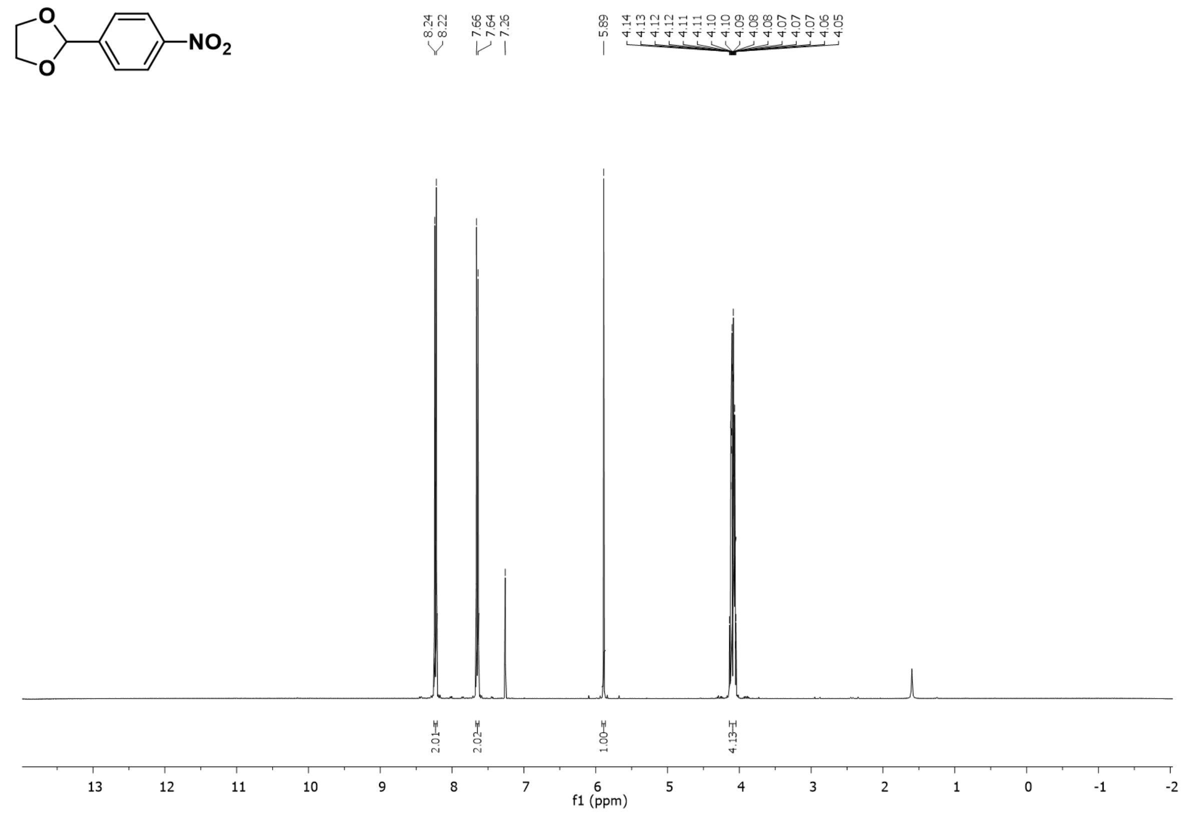

Supplementary Figure 51 ^{1}H NMR of **5** in $CDCl_3$

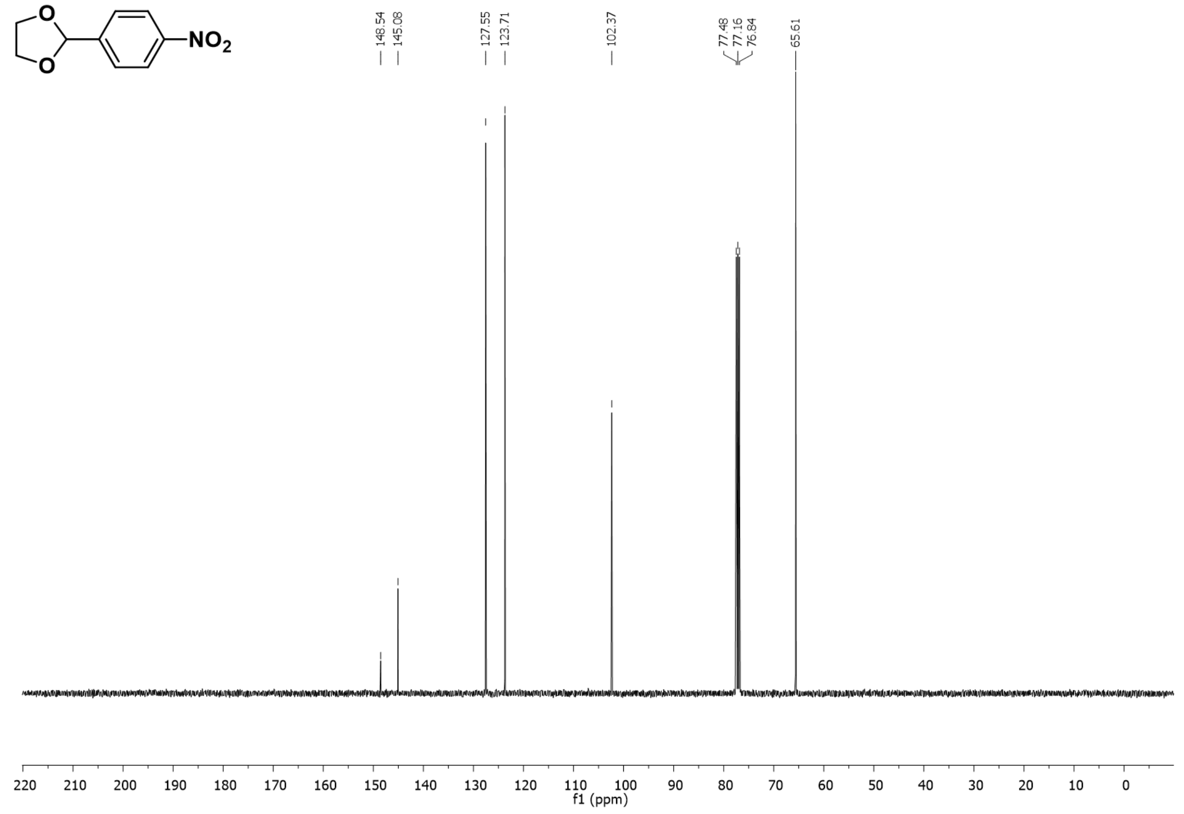

Supplementary Figure 52 ^{13}C NMR of **5** in $CDCl_3$

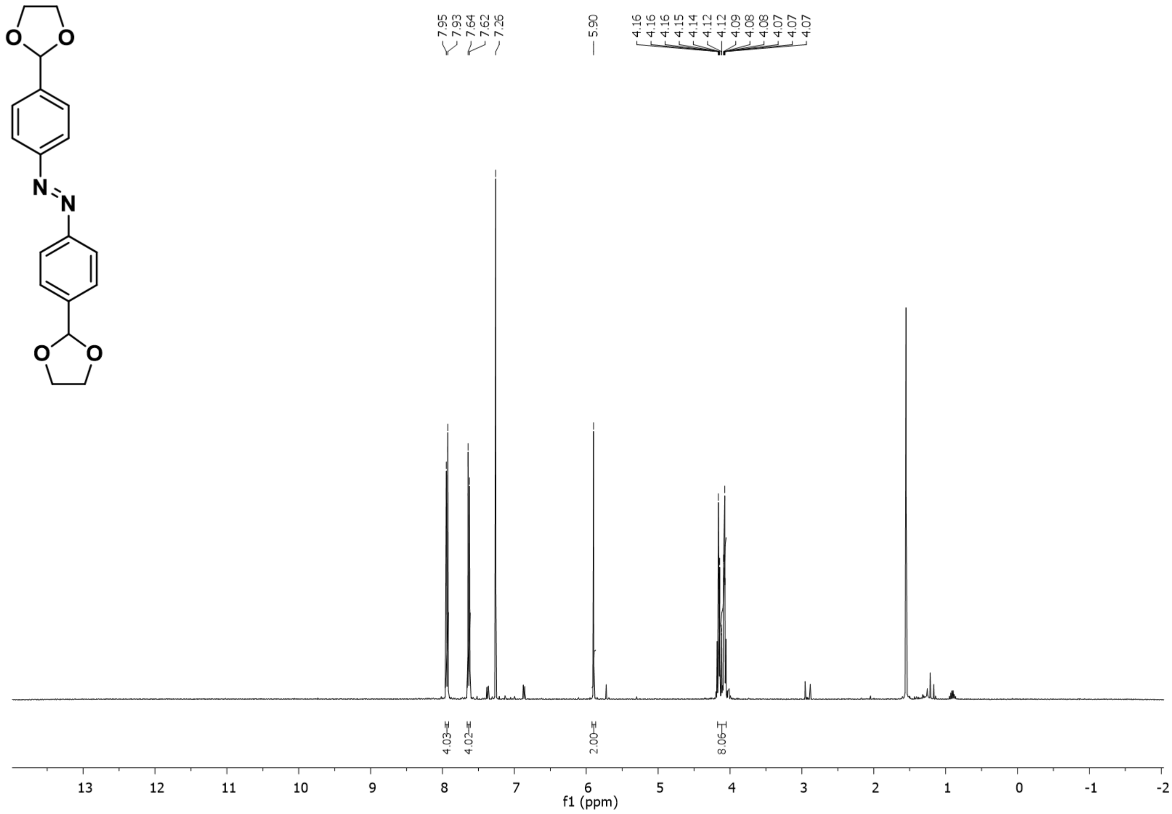

Supplementary Figure 53 ^{1}H NMR of **6** in $CDCl_3$

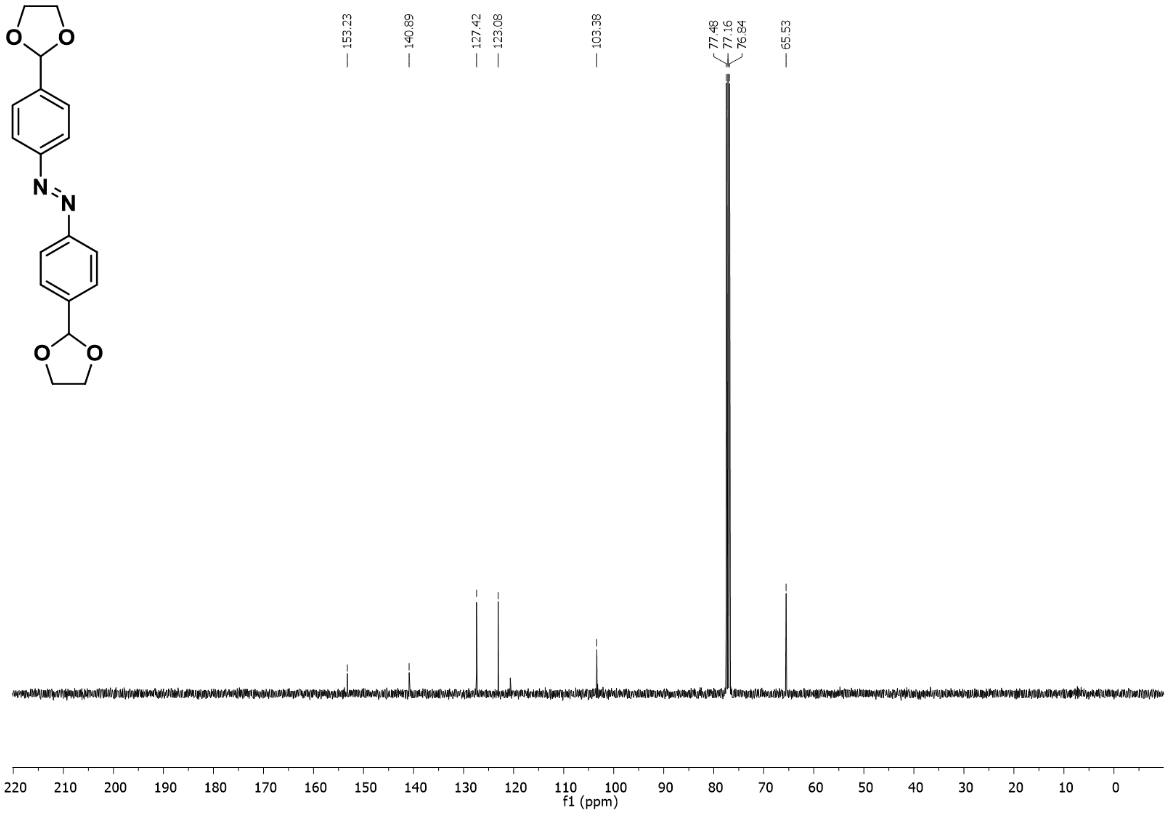

Supplementary Figure 54 ^{13}C NMR of **6** in $CDCl_3$

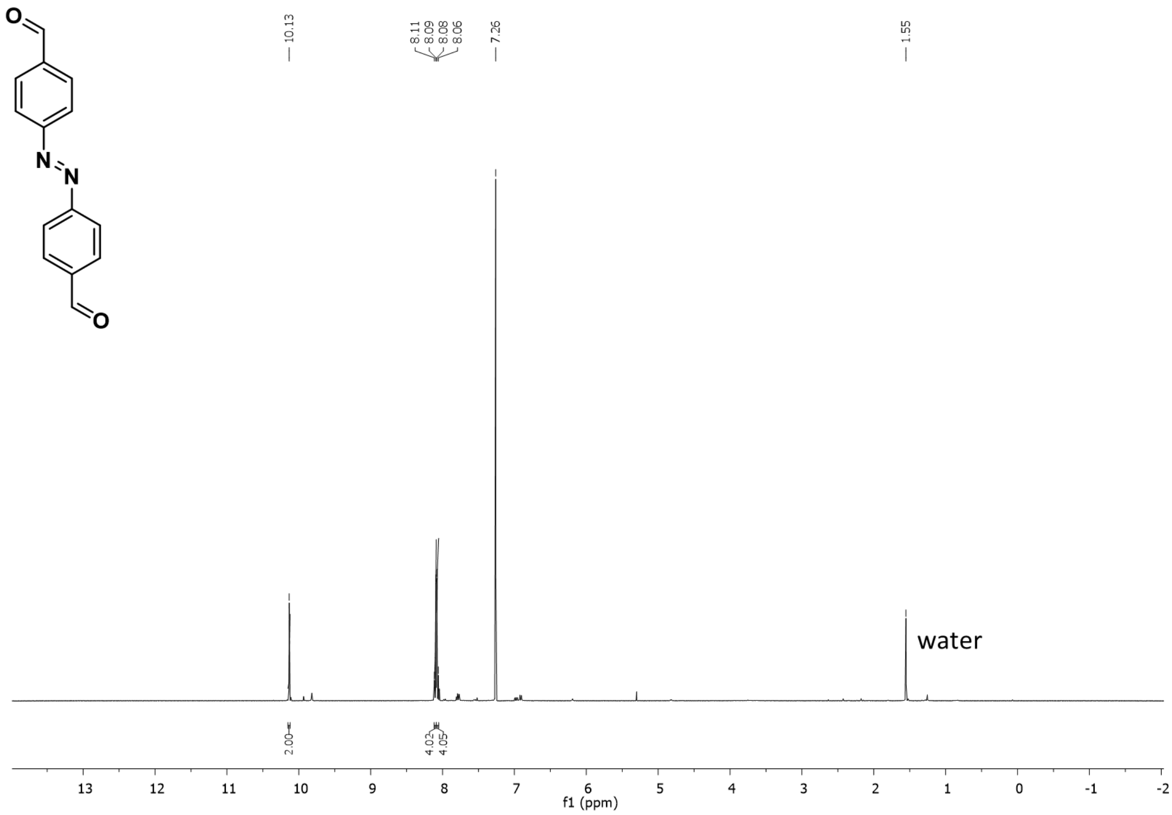

Supplementary Figure 55 ^{1}H NMR of **7** in $CDCl_3$

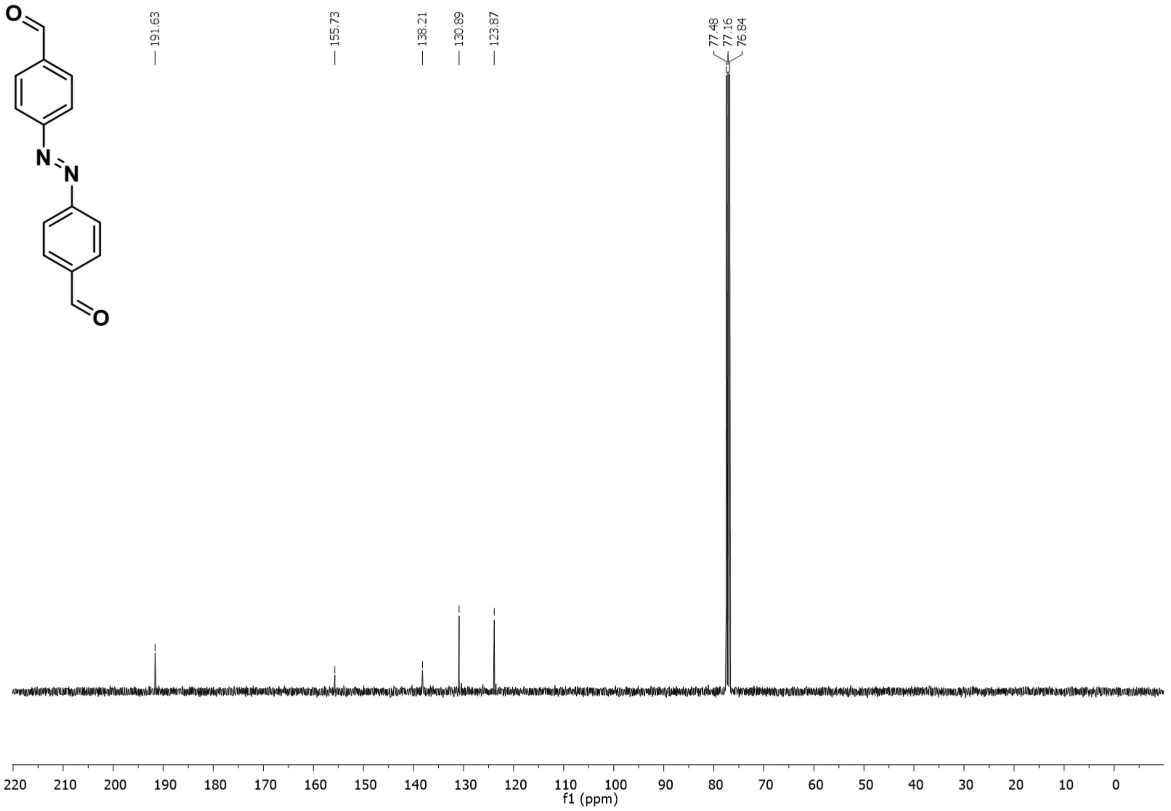

Supplementary Figure 56 ^{13}C NMR of **7** in $CDCl_3$

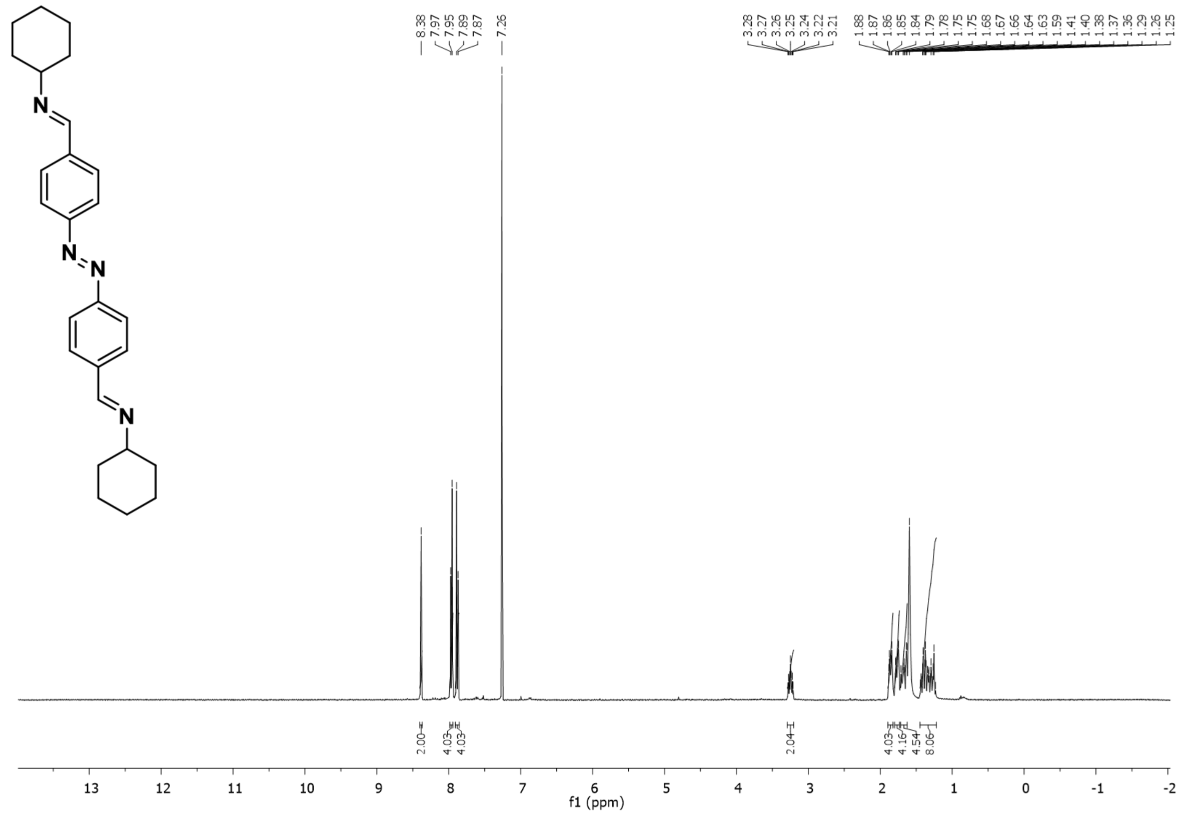

Supplementary Figure 57 ^{13}C NMR of **A2** in $CDCl_3$

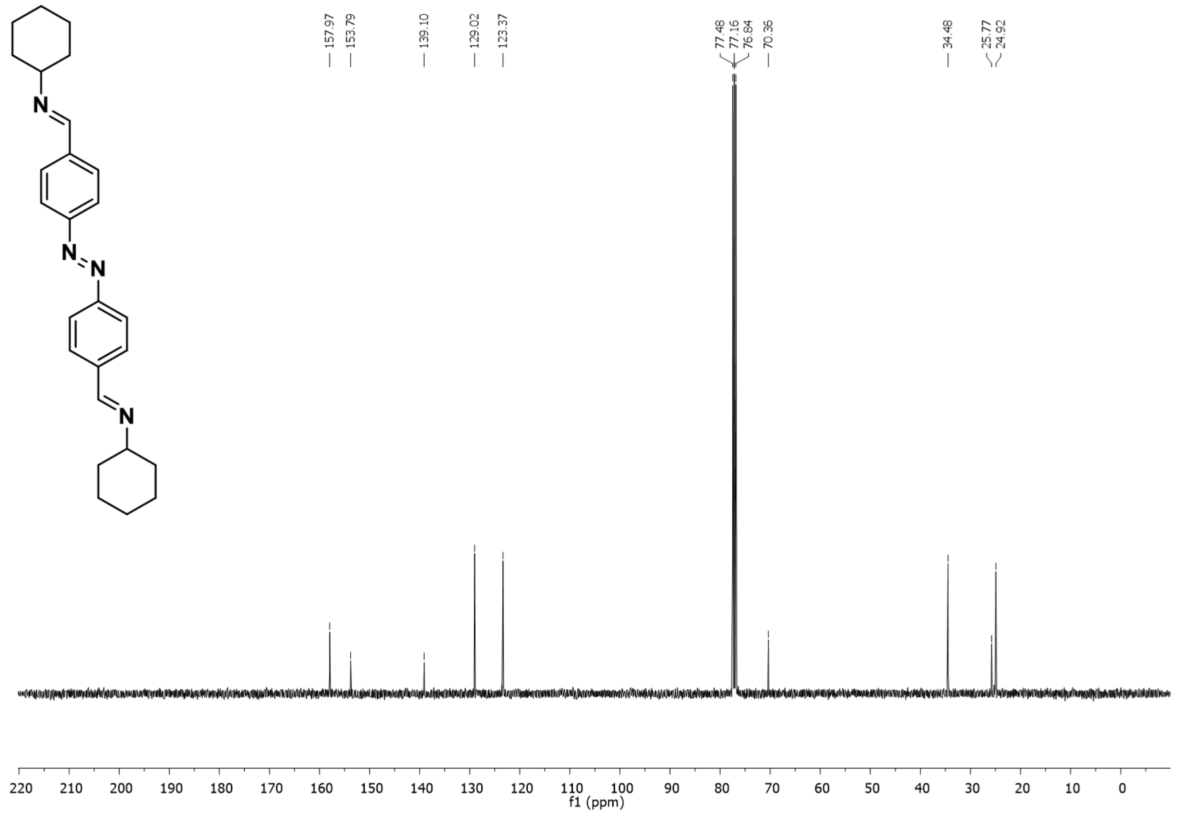

Supplementary Figure 58 ^{13}C NMR of **A2** in $CDCl_3$

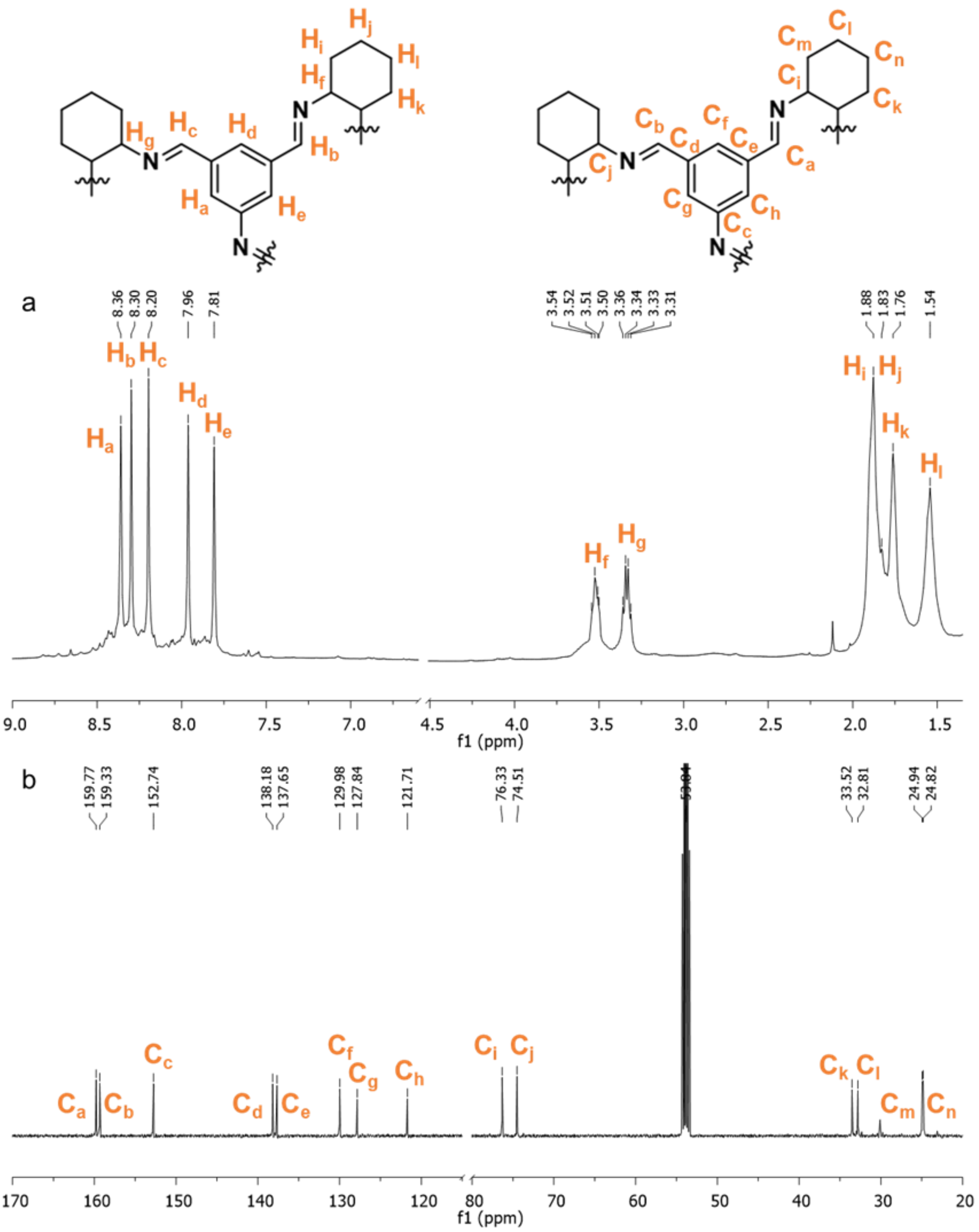

Supplementary Figure 59 Proton and carbon assignments of **ACC-1** from 1D and 2D NMR interpretation, where a) assigned ^{1}H NMR spectrum in CD_2Cl_2; b) assigned ^{13}C NMR spectrum in CD_2Cl_2.

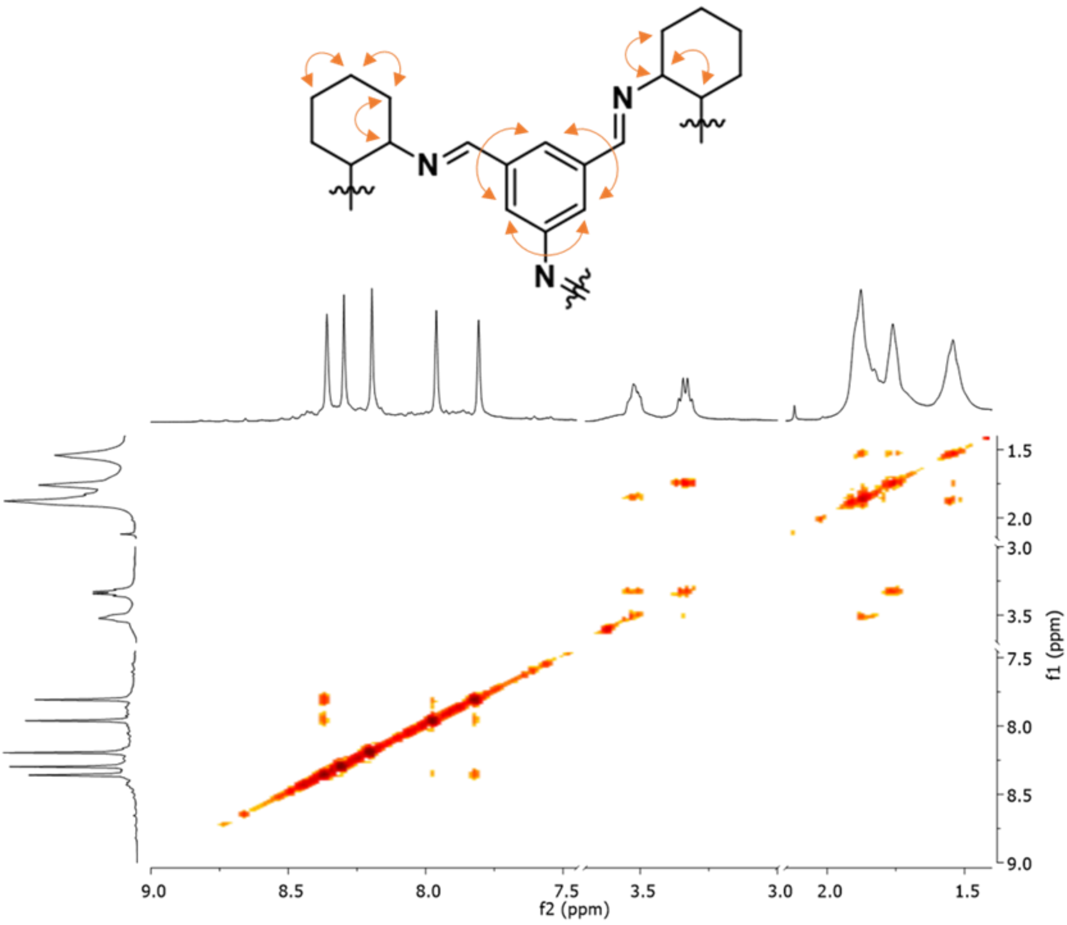

Supplementary Figure 60 2D COSY NMR (^{1}H – ^{1}H) spectrum of **ACC-1** in CD_2Cl_2 with coupling interactions labelled as orange arrows.

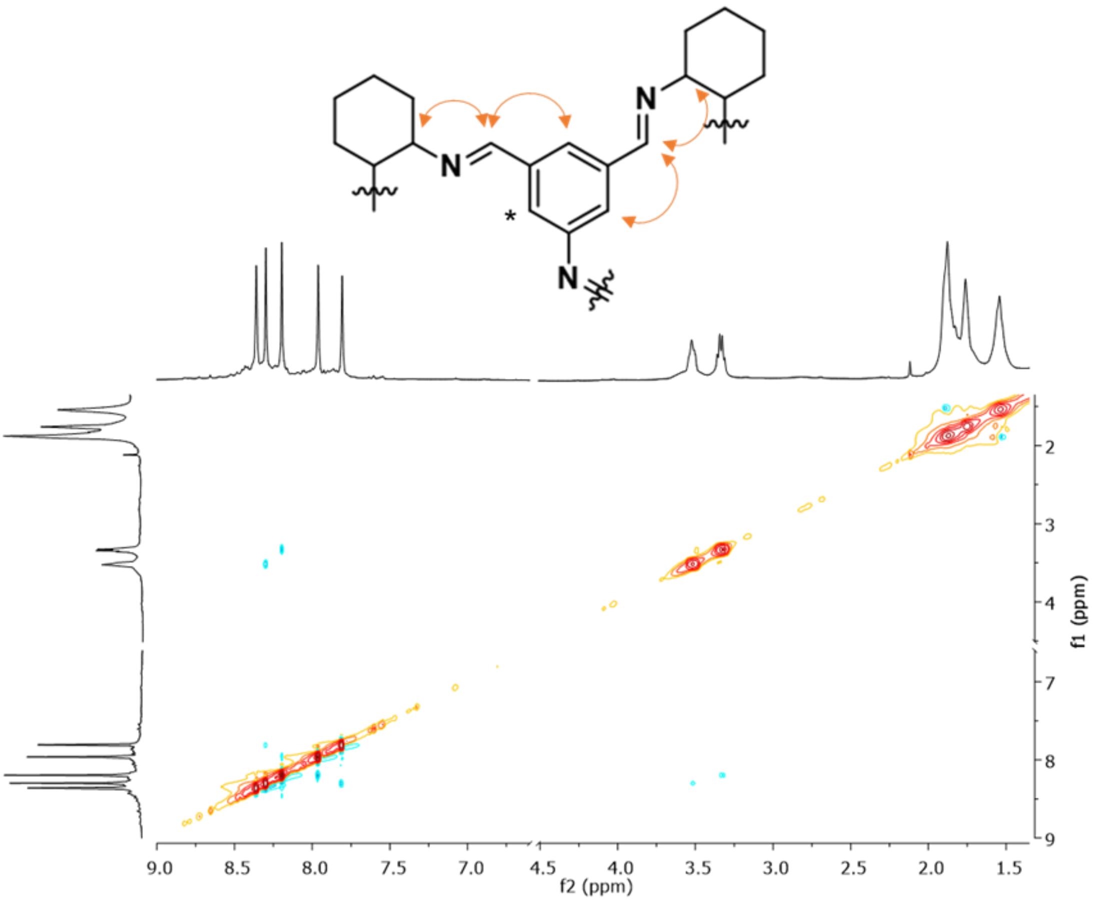

Supplementary Figure 61 2D NOESY NMR (^{1}H – ^{1}H) spectrum of **ACC-1** in CD_2Cl_2 with coupling interactions labelled as orange arrows. The proton marked with an asterisk (*) shows no imine interaction.

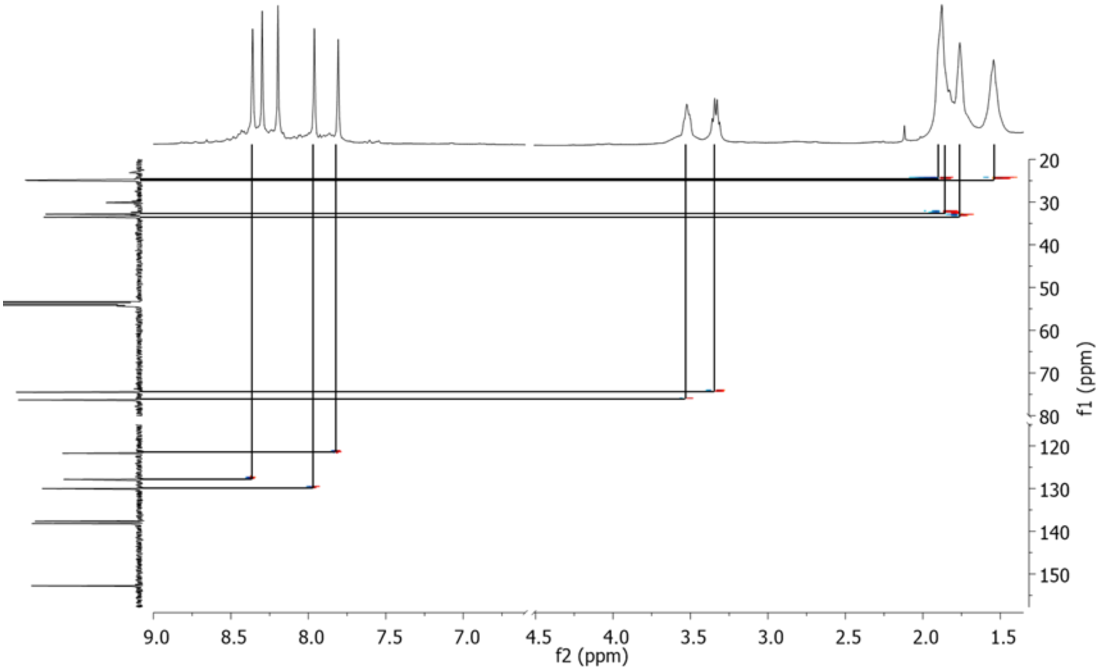

Supplementary Figure 62 2D HSQC NMR (^{1}H – ^{13}C) spectrum of **ACC-1** in CD_2Cl_2 with the $^1J_{CH}$ couplings displayed with a black line.

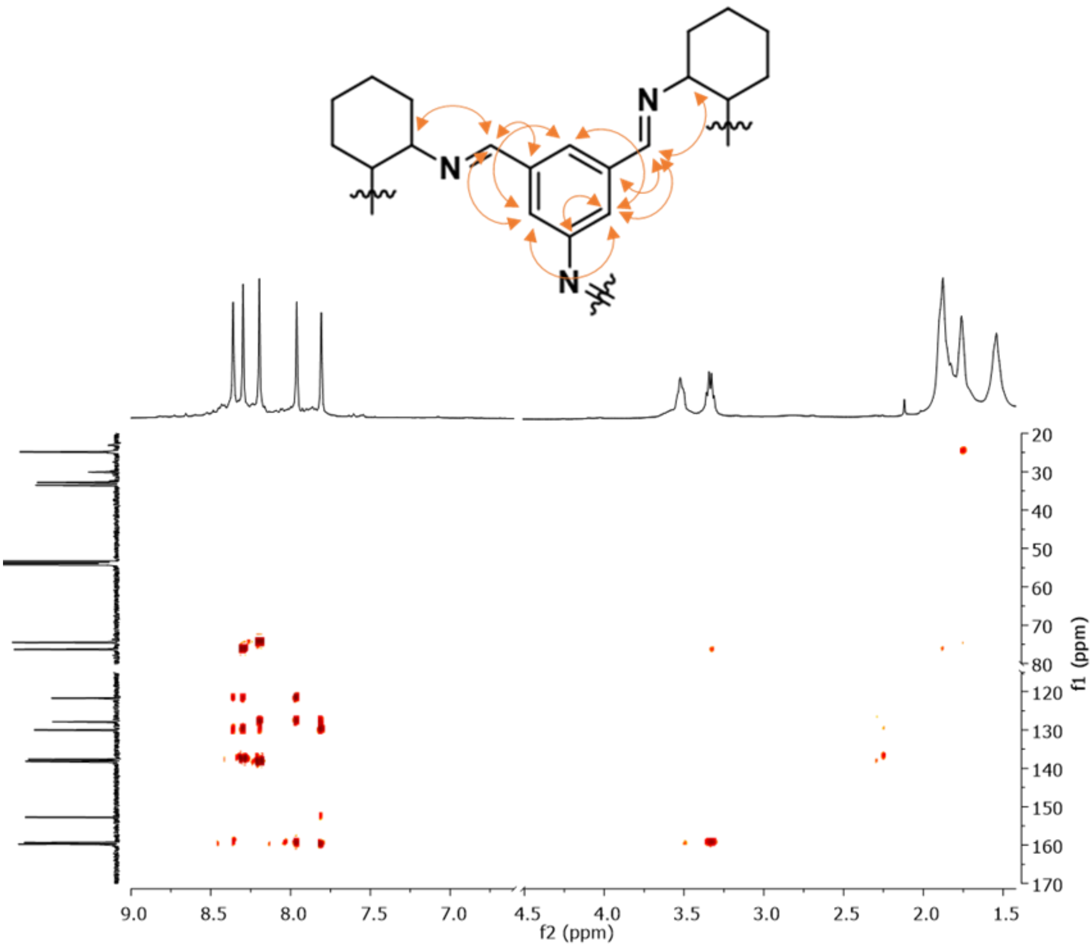

Supplementary Figure 63 2D HMBC NMR (^{1}H – ^{13}C) spectrum of **ACC-1** in CD_2Cl_2 with key interactions labelled as orange arrows.

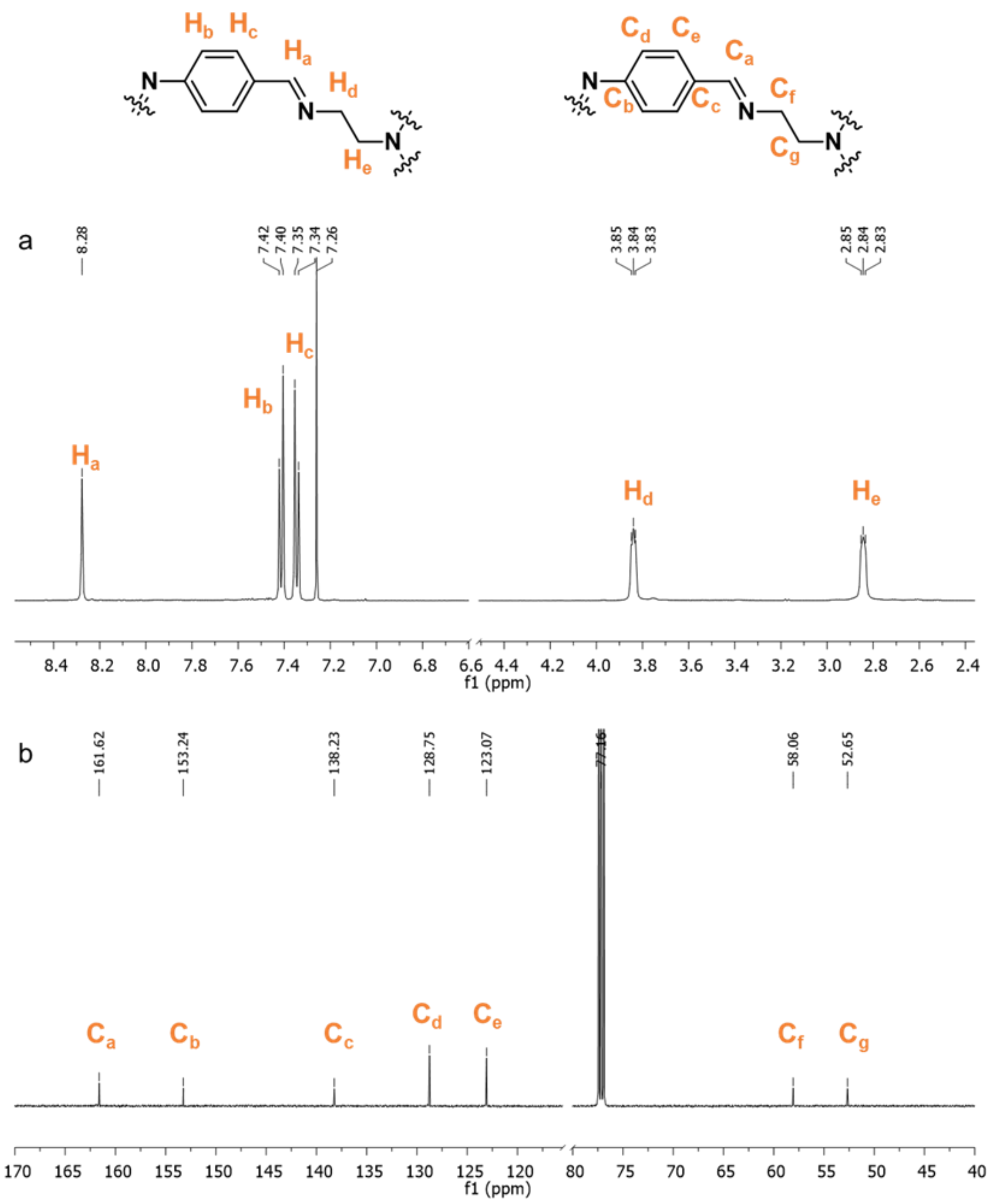

Supplementary Figure 64 Proton and carbon assignments of **ACC-2** from 1D and 2D NMR interpretation, where: a) assigned ^{1}H NMR spectrum in $CDCl_3$; b) assigned ^{13}C NMR spectrum in $CDCl_3$.

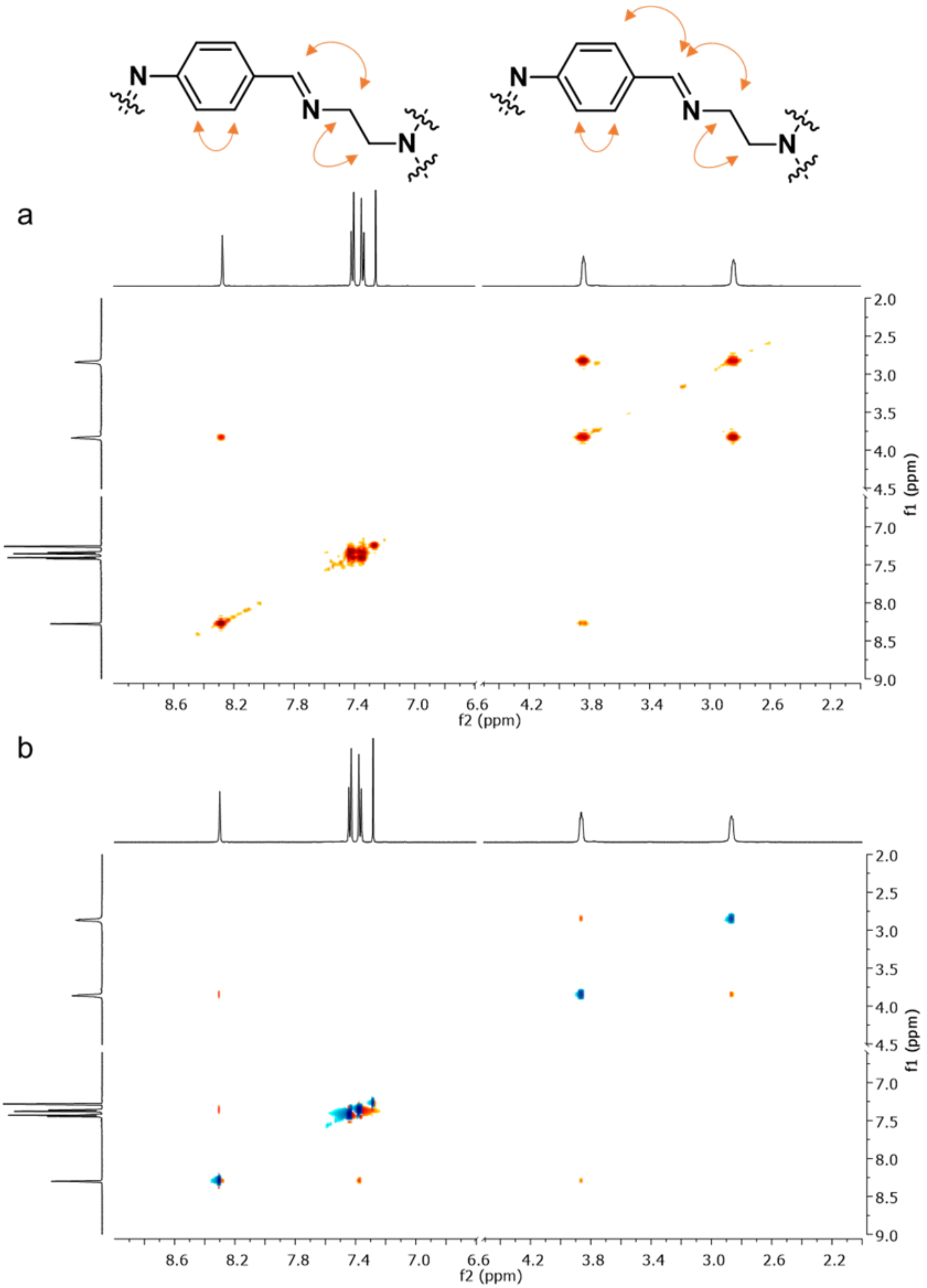

Supplementary Figure 65 Key couplings observed in the ^{1}H –^{1}H 2D NMR spectra of **ACC-2**, where: a) 2D COSY NMR spectrum in $CDCl_3$; b) 2D NOESY NMR spectrum in $CDCl_3$.

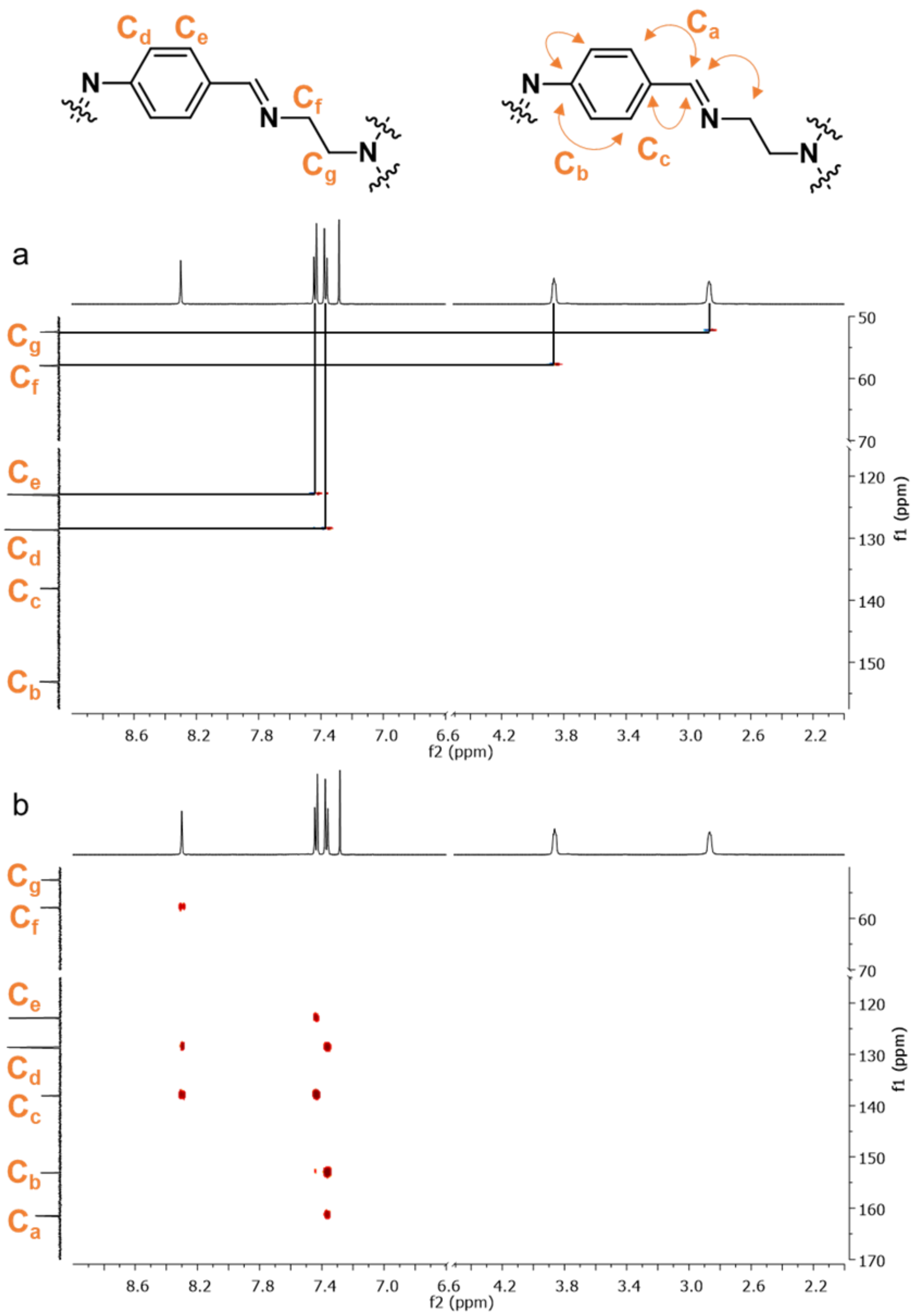

Supplementary Figure 66 Carbon NMR assignments of **ACC-2**, where: a) HSQC (^{1}H – ^{13}C) spectrum of **ACC-2**; b) HMBC (^{1}H – ^{13}C) spectrum of **ACC-2**.

10. References

(1) Liu, M.; Mao, X.; Ye, C.; Huang, H.; Nicholson, J. K.; Lindon, J. C. Improved WATERGATE Pulse Sequences for Solvent Suppression in NMR Spectroscopy. *J. Magn. Reson.* **1998**, *132* (1), 125–129. https://doi.org/10.1006/jmre.1998.1405.

(2) Sheldrick, G. M. *SHELXT* – Integrated Space-Group and Crystal-Structure Determination. *Acta Crystallogr. Sect. Found. Adv.* **2015**, *71* (1), 3–8. https://doi.org/10.1107/S2053273314026370.

(3) Dolomanov, O. V.; Bourhis, L. J.; Gildea, R. J.; Howard, J. A. K.; Puschmann, H. *OLEX2* : A Complete Structure Solution, Refinement and Analysis Program. *J. Appl. Crystallogr.* **2009**, *42* (2), 339–341. https://doi.org/10.1107/S0021889808042726.

(4) Spek, A. L. PLATON, An Integrated Tool for the Analysis of the Results of a Single Crystal Structure Determination. *Acta Crystallogr Sect A* **1990**, *46* ((s1)), c34.

(5) Van Der Sluis, P.; Spek, A. L. BYPASS: An Effective Method for the Refinement of Crystal Structures Containing Disordered Solvent Regions. *Acta Crystallogr. A* **1990**, *46* (3), 194–201. https://doi.org/10.1107/S0108767389011189.

(6) Fischer, E. Calculation of Photostationary States in Systems A .Dblarw. B When Only A Is Known. *J. Phys. Chem.* **1967**, *71* (11), 3704–3706. https://doi.org/10.1021/j100870a063.

(7) Berdnikova, D. V. Aurones: Unexplored Visible-Light Photoswitches for Aqueous Medium. *Chem. – Eur. J.* **2024**, *30* (20), e202304237. https://doi.org/10.1002/chem.202304237.

(8) Koeppe, B.; Schröder, V. R. F. Effects of Polar Substituents and Media on the Performance of *N* , *N'* -Di- *Tert* -Butoxycarbonyl-Indigos as Photoswitches. *ChemPhotoChem* **2019**, *3* (8), 613–618. https://doi.org/10.1002/cptc.201900032.

(9) Blanc, J.; Ross, D. L. Procedure for Determining the Absorption Spectra of Mixed Photochromic Isomers Not Requiring Their Separation. *J. Phys. Chem.* **1968**, *72* (8), 2817–2824. https://doi.org/10.1021/j100854a022.

(10) Griffiths, R.-R.; Greenfield, J. L.; Thawani, A. R.; Jamasb, A. R.; Moss, H. B.; Bourached, A.; Jones, P.; McCorkindale, W.; Aldrick, A. A.; Fuchter, M. J.; Lee, A. A. Data-Driven Discovery of Molecular Photoswitches with Multioutput Gaussian Processes. *Chem. Sci.* **2022**, *13* (45), 13541–13551. https://doi.org/10.1039/D2SC04306H.

(11) Berdnikova, D. V. Design, Synthesis and Investigation of Water-Soluble Hemi-Indigo Photoswitches for Bioapplications. *Beilstein J. Org. Chem.* **2019**, *15*, 2822–2829. https://doi.org/10.3762/bjoc.15.275.

(12) Rau, Hermann.; Greiner, Gerhard.; Gauglitz, Guenter.; Meier, Herbert. Photochemical Quantum Yields in the A (+h.Nu.) .Dblarw. B (+h.Nu.,.DELTA.) System When Only the Spectrum of A Is Known. *J. Phys. Chem.* **1990**, *94* (17), 6523–6524. https://doi.org/10.1021/j100380a003.

(13) Turcani, L.; Berardo, E.; Jelfs, K. E. *Stk* : A Python Toolkit for Supramolecular Assembly. *J. Comput. Chem.* **2018**, *39* (23), 1931–1942. https://doi.org/10.1002/jcc.25377.

(14) Roos, K.; Wu, C.; Damm, W.; Reboul, M.; Stevenson, J. M.; Lu, C.; Dahlgren, M. K.; Mondal, S.; Chen, W.; Wang, L.; Abel, R.; Friesner, R. A.; Harder, E. D. OPLS3e: Extending Force Field Coverage for Drug-Like Small Molecules. *J. Chem. Theory Comput.* **2019**, *15* (3), 1863–1874. https://doi.org/10.1021/acs.jctc.8b01026.

(15) VandeVondele, J.; Krack, M.; Mohamed, F.; Parrinello, M.; Chassaing, T.; Hutter, J. Quickstep: Fast and Accurate Density Functional Calculations Using a Mixed Gaussian and Plane Waves Approach. *Comput. Phys. Commun.* **2005**, *167* (2), 103–128. https://doi.org/10.1016/j.cpc.2004.12.014.

(16) Perdew, J. P.; Burke, K.; Ernzerhof, M. Generalized Gradient Approximation Made Simple. *Phys. Rev. Lett.* **1996**, *77* (18), 3865–3868. https://doi.org/10.1103/PhysRevLett.77.3865.

(17) Khaliullin, R. Z.; VandeVondele, J.; Hutter, J. Efficient Linear-Scaling Density Functional Theory for Molecular Systems. *J. Chem. Theory Comput.* **2013**, *9* (10), 4421–4427. https://doi.org/10.1021/ct400595k.

(18) Grimme, S.; Antony, J.; Ehrlich, S.; Krieg, H. A Consistent and Accurate *Ab Initio* Parametrization of Density Functional Dispersion Correction (DFT-D) for the 94 Elements H-Pu. *J. Chem. Phys.* **2010**, *132* (15), 154104. https://doi.org/10.1063/1.3382344.
(19) Greenaway, R. L.; Santolini, V.; Bennison, M. J.; Alston, B. M.; Pugh, C. J.; Little, M. A.; Miklitz, M.; Eden-Rump, E. G. B.; Clowes, R.; Shakil, A.; Cuthbertson, H. J.; Armstrong, H.; Briggs, M. E.; Jelfs, K. E.; Cooper, A. I. High-Throughput Discovery of Organic Cages and Catenanes Using Computational Screening Fused with Robotic Synthesis. *Nat. Commun.* **2018**, *9* (1), 2849. https://doi.org/10.1038/s41467-018-05271-9.
(20) Berardo, E.; Greenaway, R. L.; Turcani, L.; Alston, B. M.; Bennison, M. J.; Miklitz, M.; Clowes, R.; Briggs, M. E.; Cooper, A. I.; Jelfs, K. E. Computationally-Inspired Discovery of an Unsymmetrical Porous Organic Cage. *Nanoscale* **2018**, *10* (47), 22381–22388. https://doi.org/10.1039/C8NR06868B.
(21) Miklitz, M.; Jelfs, K. E. Pywindow : Automated Structural Analysis of Molecular Pores. *J. Chem. Inf. Model.* **2018**, *58* (12), 2387–2391. https://doi.org/10.1021/acs.jcim.8b00490.
(22) Willems, T. F.; Rycroft, C. H.; Kazi, M.; Meza, J. C.; Haranczyk, M. Algorithms and Tools for High-Throughput Geometry-Based Analysis of Crystalline Porous Materials. *Microporous Mesoporous Mater.* **2012**, *149* (1), 134–141. https://doi.org/10.1016/j.micromeso.2011.08.020.
(23) Macrae, C. F.; Sovago, I.; Cottrell, S. J.; Galek, P. T. A.; McCabe, P.; Pidcock, E.; Platings, M.; Shields, G. P.; Stevens, J. S.; Towler, M.; Wood, P. A. *Mercury 4.0* : From Visualization to Analysis, Design and Prediction. *J. Appl. Crystallogr.* **2020**, *53* (1), 226–235. https://doi.org/10.1107/S1600576719014092.
(24) Yu, X.-H.; Peng, C.-C.; Sun, X.-X.; Chen, W.-H. Synthesis, Anionophoric Activity and Apoptosis-Inducing Bioactivity of Benzimidazolyl-Based Transmembrane Anion Transporters. *Eur. J. Med. Chem.* **2018**, *152*, 115–125. https://doi.org/10.1016/j.ejmech.2018.04.036.
(25) Reddy, N. R.; Kumar, R.; Baskaran, S. A Direct Method for the Efficient Synthesis of Benzylidene Acetal at Room Temperature. *Eur. J. Org. Chem.* **2019**, *2019* (7), 1548–1552. https://doi.org/10.1002/ejoc.201801731.
(26) Eyring, H. The Activated Complex in Chemical Reactions. *J. Chem. Phys.* **1935**, *3* (2), 107–115. https://doi.org/10.1063/1.1749604.
(27) Lange, N. A.; Forker, G. M. *Handbook of Chemistry*, 10TH ED.; McGraw-Hill Book Company Inc.
(28) *CRC Handbook of Chemistry and Physics*; CRC Press, Taylor & Francis Group, 2014.
(29) Ohligschläger, A.; Menzel, K.; Ten Kate, A.; Martinez, J. R.; Frömbgen, C.; Arts, J.; McCulloch, A.; Rossberg, M.; Lendle, W.; Pfleiderer, G. *Chloromethanes In Ullmann's Encyclopedia of Industrial Chemistry*; 2019.
